# Iron(III)-Mediated Rapid Radical-Type Three-Component Deuteration of Quinoxalinones With Olefins and NaBD_4_

**DOI:** 10.3389/fchem.2020.00606

**Published:** 2020-08-04

**Authors:** Wanmei Li, Heng Cai, Lin Huang, Lei He, Yilan Zhang, Jun Xu, Pengfei Zhang

**Affiliations:** College of Material, Chemistry and Chemical Engineering, Hangzhou Normal University, Hangzhou, China

**Keywords:** deuteration, quinoxalinones, alkenes, three-component, radical pathway

## Abstract

Iron(III)-promoted rapid three-component deuteration of quinoxalinones with olefins and NaBD_4_ is reported for the first time, which provides a novel, economic, and efficient method for the rapid synthesis of deuterated quinoxalinones. In this transformation, a radical pathway is involved according to the results of control experiments.

## Introduction

**Scheme 1:**
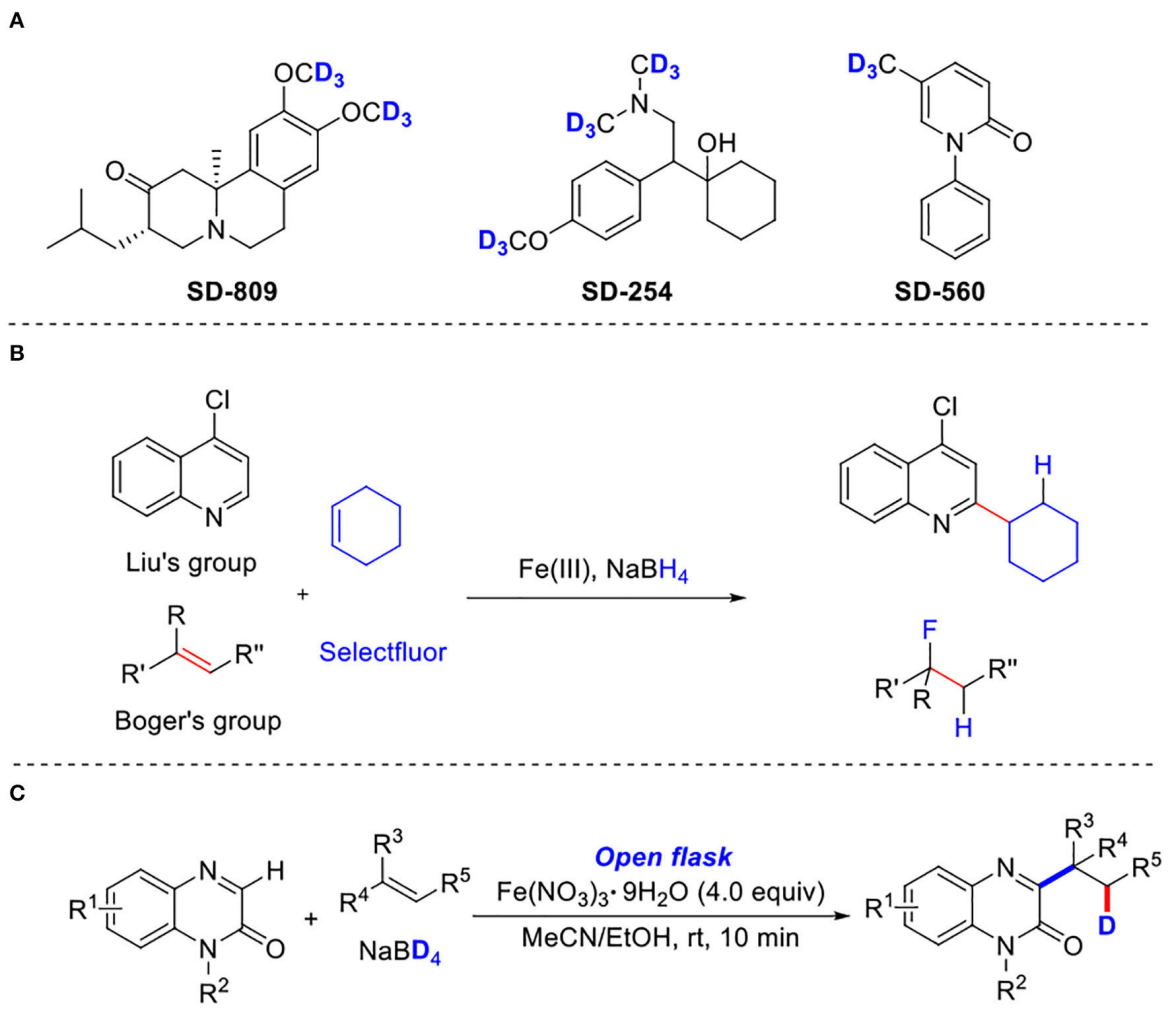
Three-component deuteration of quinoxalinones. **(A)** Representative bioactive compounds that contain deuterium. **(B)** Previous work Fe(III)-promoted three-component deuteration. **(C)** Present Work: Iron (III)-promoted three-component deuteration.

In recent years, deuterium-labeled compounds have received much attention because they play an important role in studying chemical and biological processes (Mutlib, 2008; Gómez-Gallego and Sierra, 2011; Konermann et al., 2011; Simmons and Hartwig, 2012; Atzrodt et al., 2018; Pirali et al., 2019). The incorporation of deuterium is a very efficient strategy not only to measure the kinetic isotope effect and track the reaction path in synthetic chemistry but also to change the absorption, distribution, metabolism, and excretion (ADME) properties of drug candidates in pharmaceutical chemistry (Atzrodt et al., 2007; Meanwell, 2011; Guengerich, 2012; Katsnelson, 2013; Gant, 2014). Since the first deuterated drug, deutetrabenazine, for the treatment of chorea associated with Huntington's disease was approved by the Food and Drug Administration in 2017 (Schmidt, 2017), which clearly proved a route for the development of deuterated drugs in clinical medicine (Scheme 1A) (Junk and Catallo, 1997; Gowrisankar et al., 2012; Tolnai et al., 2014; Ray et al., 2018), considerable interests have been devoted to developing novel and efficient methods for the synthesis of such compounds (Yu et al., 2016; Kerr et al., 2017; Liang et al., 2017; Li et al., 2017; Liu et al., 2018; Yang et al., 2018; Han et al., 2019; Shen et al., 2019; Xu et al., 2019; Zhao et al., 2019; Chang et al., 2020; Dong et al., 2020). For instances, in 2016, Chirik and coworkers reported an iron-catalyzed transformation for the deuteration and tritiation of pharmaceuticals (Yu et al., 2016). Kerr's group developed an iridium-catalyzed hydrogen isotope exchange method for the site-selective deuteration of *N*-heterocycles (Kerr et al., 2017). In 2012, Fe(III)/NaBH_4_-mediated free radical hydrofluorination of unactivated alkenes was reported by Boger's group (Barker and Boger, 2012) (Scheme 1B). Subsequently, Liu and coworkers reported a similar method with Fe(III)-promoted free-radical hydroheteroarylation of alkenes (Liang et al., 2017) (Scheme 1B). Dai and Yan, respectively developed novel methods for the synthesis of deuterated arenes by a palladium-catalyzed, pyridine-directed remote *meta*-C–H bond deuteration of arenes (Xu et al., 2019) or ruthenium catalysis (Zhao et al., 2019). In 2019, Wasa and coworkers demonstrated a B(C_6_F_5_)_3_-catalyzed α-deuteration of carbonyl compounds with D_2_O, providing an efficient protocol for the synthesis of deuterium labeling carbonyl-based pharmaceuticals (Chang et al., 2020). Despite their utilities, there is still a substantial interest in developing novel and efficient methods for the synthesis of such organic compounds.

Multicomponent reactions have become a hot field in modern organic chemistry in recent years because multicomponent reaction can form multiple chemical bonds in one step in comparison with the traditional synthesis method, thus realizing the simple, efficient, and atomic economic synthesis of structural diversity compounds. Quinoxalines and their derivatives are one of the important organic compounds because they have been widely applied in organic synthesis, material chemistry, agrochemical industries, and pharmaceutical chemistry (TenBrink et al., 1994; Monge et al., 1995; Badran et al., 2003; Refaat et al., 2004; Hoogewijs et al., 2013; Nakane et al., 2015; Renault et al., 2017). Although a plenty of two-component reactions for the synthesis of quinoxalinones were achieved (Hong et al., 2019; Jin et al., 2019; Ke et al., 2019; Liu et al., 2019; Wang et al., 2019, 2020; Wei et al., 2019; Xie et al., 2019; Xue et al., 2019; Yan et al., 2019; Zhang H. et al., 2019; Zhang W. et al., 2019; Bao et al., 2020).

Multicomponent transformations were rarely reported. In 2019, Studer and coworkers demonstrated a visible-light-initiated three-component reaction of quinoxalinones, olefins, and perfluoroalkyl iodides (Zheng and Studer, 2019). In the same year, Koley's group disclosed a metal-free domino three-component radical cascde reaction of quinoxalinones, olefins, and sulfinic acids (Dutta et al., 2019).

We also achieved a useful method for the rapid synthesis of quinoxalinone-containing organoazides using three-component cascade reaction of quinoxalinones with olefins and TMSN_3_ (Shen et al., 2020). Keeping on our interests in developing simple and efficient methods for the synthesis of quinoxalinones (Xu et al., 2019; Zhang H. et al., 2019; Shen et al., 2020), herein, we demonstrated a radical-type three-component deuteration of quinoxalinones with olefins and NaBD_4_ mediated by Fe(NO_3_)_3_∙9H_2_O for the first time (Scheme 1C).

## Results and Discussion

Initially, we commenced three-component deuteration of quinoxalinones by the reaction of 1-methylquinoxalin-2(1*H*)-one (**1a**), 2.0 equiv of styrene (**2a**), 1.0 equiv of NaBD_4_ (**3**) 4.0 equiv of Fe(NO_3_)_3_•9H_2_O in ethanol at room temperature for 10 min, providing the desired product **4a** in 55% yield (Table 1, entry 1). This reaction could not take place if other solvents (MeCN, dichloromethane (DCM), dioxane, dimethylformamide (DMF), dimethyl sulfoxide (DMSO)] (Table 1, entries 2–6) or catalysts (Table 1, entries 12–17) [FeBr_3_, CuCl_2_, CuO, (NH_4_)_2_Ce(NO_3_)_6_, Fe_2_(ox)_3_, FeF_3_], or no catalyst (Table 1, entry 18) were used. However, it surprised us that using the mixed solvent of ethanol and acetonitrile (*v*/*v* = 1:1) could improve the reaction yield to 65% (Table 1, entry 7 among entries 7–11). Subsequently, the dosage of styrene **2a** and NaBD_4_ **3** were screened (Table 1, entries 19–23). The yield was decreased to 40% when amount of **2a** was reduced from 2.0 to 1.0 equiv (Table 1, entry 19). By increasing the amount of NaBD_4_ **3** from 1.0 to 2.0 equiv, a highest yield (73%) was observed (Table 1, entry 23). Furthermore, the product yield could also not be further improved no matter changing the amount of Fe(NO_3_)_3_•9H_2_O (Table 1, entries 24–25) or reaction time (Table 1, entries 26–27). Thus, the highest yield could be obtained when the mixture of 1-methylquinoxalin-2(1*H*)-one (**1a**), 2.0 equiv of styrene (**2a**), 2.0 equiv of NaBD_4_ (**3**) in EtOH/CH_3_CN (4.0 ml, *v*/*v* = 1:1) were reacted at 4.0 equiv of Fe(NO_3_)_3_•9H_2_O as oxidant at room temperature for 10 min.

**Table 1:** Optimization of reaction conditions^a^.

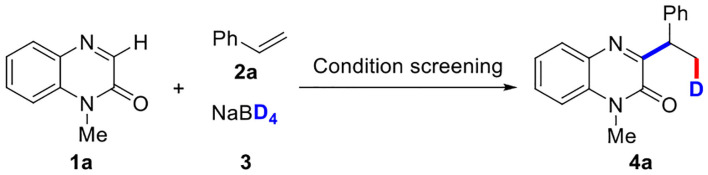					
**Entry**	**2a (*x*)**	**3(*y*)**	**Oxidant**	**Solvent**	**Yield (%)^**b**^**
1	2	1	Fe(NO_3_)_3_•9H_2_O	EtOH	55
2	2	1	Fe(NO_3_)_3_•9H_2_O	MeCN	0
3	2	1	Fe(NO_3_)_3_•9H_2_O	DCM	0
4	2	1	Fe(NO_3_)_3_•9H_2_O	Dioxane	0
5	2	1	Fe(NO_3_)_3_•9H_2_O	DMF	0
6	2	1	Fe(NO_3_)_3_•9H_2_O	DMSO	0
7	2	1	Fe(NO_3_)_3_•9H_2_O	MeCN/EtOH	65
8	2	1	Fe(NO_3_)_3_•9H_2_O	DCM/EtOH	60
9	2	1	Fe(NO_3_)_3_•9H_2_O	Dioxane/EtOH	30
10	2	1	Fe(NO_3_)_3_•9H_2_O	DMF/EtOH	Trace
11	2	1	Fe(NO_3_)_3_•9H_2_O	DMSO/EtOH	Trace
12	2	1	FeBr_3_	MeCN/EtOH	0
13	2	1	CuCl_2_	MeCN/EtOH	0
14	2	1	CuO	MeCN/EtOH	0
15	2	1	(NH_4_)_2_Ce(NO_3_)_6_	MeCN/EtOH	0
16	2	1	Fe_2_(ox)_3_	MeCN/EtOH	0
17	2	1	FeF_3_	MeCN/EtOH	0
18	2	1	–	MeCN/EtOH	0
19	1	1	Fe(NO_3_)_3_•9H_2_O	MeCN/EtOH	40
20	3	1	Fe(NO_3_)_3_•9H_2_O	MeCN/EtOH	65
21	3	2	Fe(NO_3_)_3_•9H_2_O	MeCN/EtOH	71
22	3	3	Fe(NO_3_)_3_•9H_2_O	MeCN/EtOH	72
23	2	2	Fe(NO_3_)_3_•9H_2_O	MeCN/EtOH	73
24^c^	2	2	Fe(NO_3_)_3_•9H_2_O	MeCN/EtOH	55
25^d^	2	2	Fe(NO_3_)_3_•9H_2_O	MeCN/EtOH	73
26^e^	2	2	Fe(NO_3_)_3_•9H_2_O	MeCN/EtOH	59
27^f^	2	2	Fe(NO_3_)_3_•9H_2_O	MeCN/EtOH	72

a *Reaction conditions: **1a** (0.2 mmol), **2a** (x equiv), **3** (y equiv), oxidant (4.0 equiv), solvent (4.0 ml, v/v = 1:1), room temperature, open flask, 10 min*.

b *Isolated yields*.

c *Fe(NO_3_)_3_•9H_2_O (3.0 equiv)*.

d *Fe(NO_3_)_3_•9H_2_O (5.0 equiv)*.

e *The reaction was performed in 5 min*.

f *The reaction was performed in 20 min*.

With the optimized reaction conditions in hand, the substrate scope of the three-component deuteration was subsequently explored by using various quinoxalinones (1) with styrene (2a) and NaBD_4_ (3) (Table 2). To our delight, a wide range of *N*-protecting groups including *N*-methyl, *N*-ethyl, *N*-butyl, *N*-cyclopropylmethyl, and *N*-esteryl groups could work well under standard conditions, affording the target products (4a−4e) in 70–77% yields. Quinoxalinones with various *N*-benzyl groups or the methoxyl, chloro, bromo, and methyl groups on the benzene ring were also tolerated in this reaction, as demonstrated with products 4f**−4**q, or 4r**−4**u in good yields. It was noteworthy that the *N*-free protecting quinoxalinone was also suitable for the transformation; the product (4v) was obtained in 66% yield. Unfortunately, other *N*-heterocycles, such as theophylline and 4-hydroxyquinazoline, could not undergo the reaction (see **SI**).

**Table 2:** Substrate scope of quinoxalinones^a, b^.

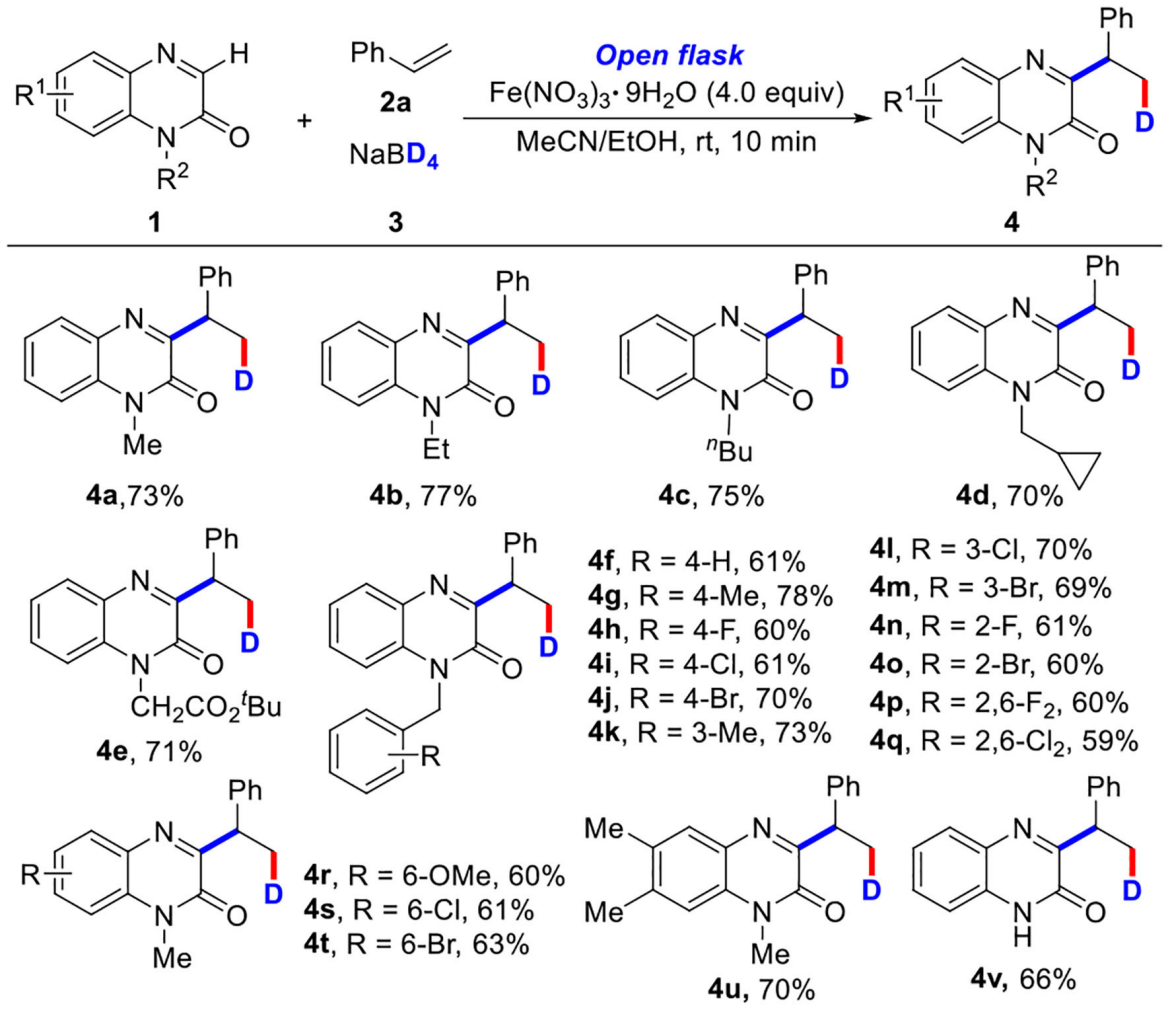

a *Reaction conditions: **1** (0.2 mmol), **2a** (2.0 equiv), **3** (2.0 equiv), Fe(NO_3_)_3_•9H_2_O (4.0 equiv), MeCN/EtOH (4.0 ml, v/v = 1:1), room temperature, open flask, 10 min*.

b *Isolated yields*.

Some other olefins were then tested by the reaction with 1-methylquinoxalin-2(1*H*)-one (1a) and NaBD_4_ (3) (Table 3). It was found that aromatic olefins bearing electron-rich or electron-poor substituents (4aa−4ai) could react smoothly, affording the desired products in good yields. The transformation with nonfunctionalized olefin was also successful, giving the corresponding product 4aj in 75% yield. The multiple substituted olefin (4ak) and cyclic olefin (4al) were also compatible, providing the target products in 60 and 76% yields, respectively (Tang et al., 2015; Yi et al., 2017). In addition, olefins with various ester substituents were also well tolerated, affording the target products (4am−4aq) in good yields. More interestingly, olefins with high-activity functional groups including halo (4ar) and alcohol substituents (4as−4av) also could be converted into corresponding products in good yields (65–79%). However, other olefins containing heteroaromatic ring, such as 2-vinylpyridine, 4-vinylpyridine, and 1-vinyl-2-pyrrolidone could not be transformed into corresponding products (see **SI**).

**Table 3:** Substrate scope of olefins^a, b^.

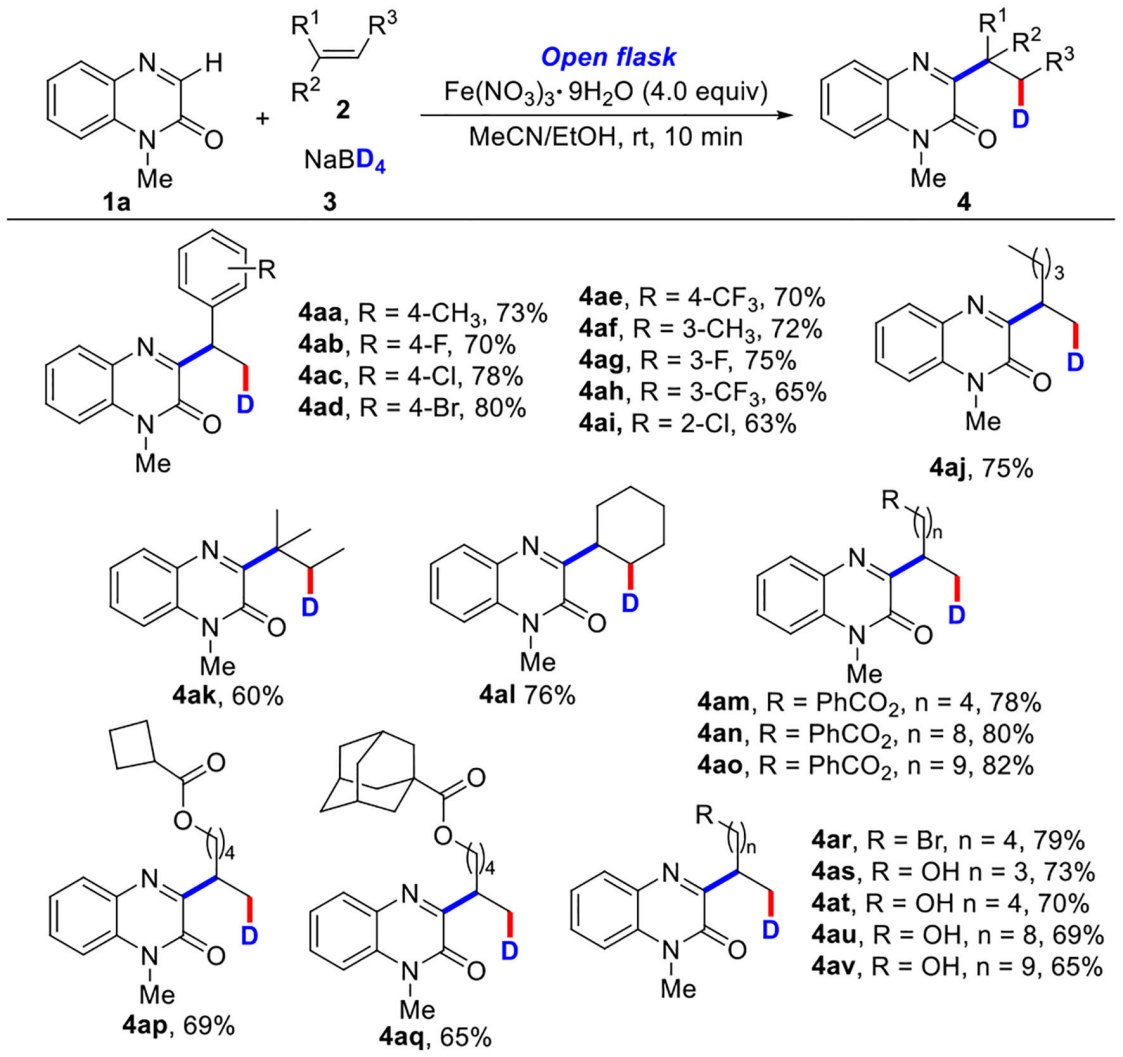

a *Reaction conditions: **1** (0.2 mmol), **2** (2.0 equiv), **3** (2.0 equiv), Fe(NO_3_)_3_•9H_2_O (4.0 equiv), MeCN/EtOH (4.0 ml, v/v = 1:1), room temperature, open flask, 10 min*.

b *Isolated yields*.

**Scheme 2:**
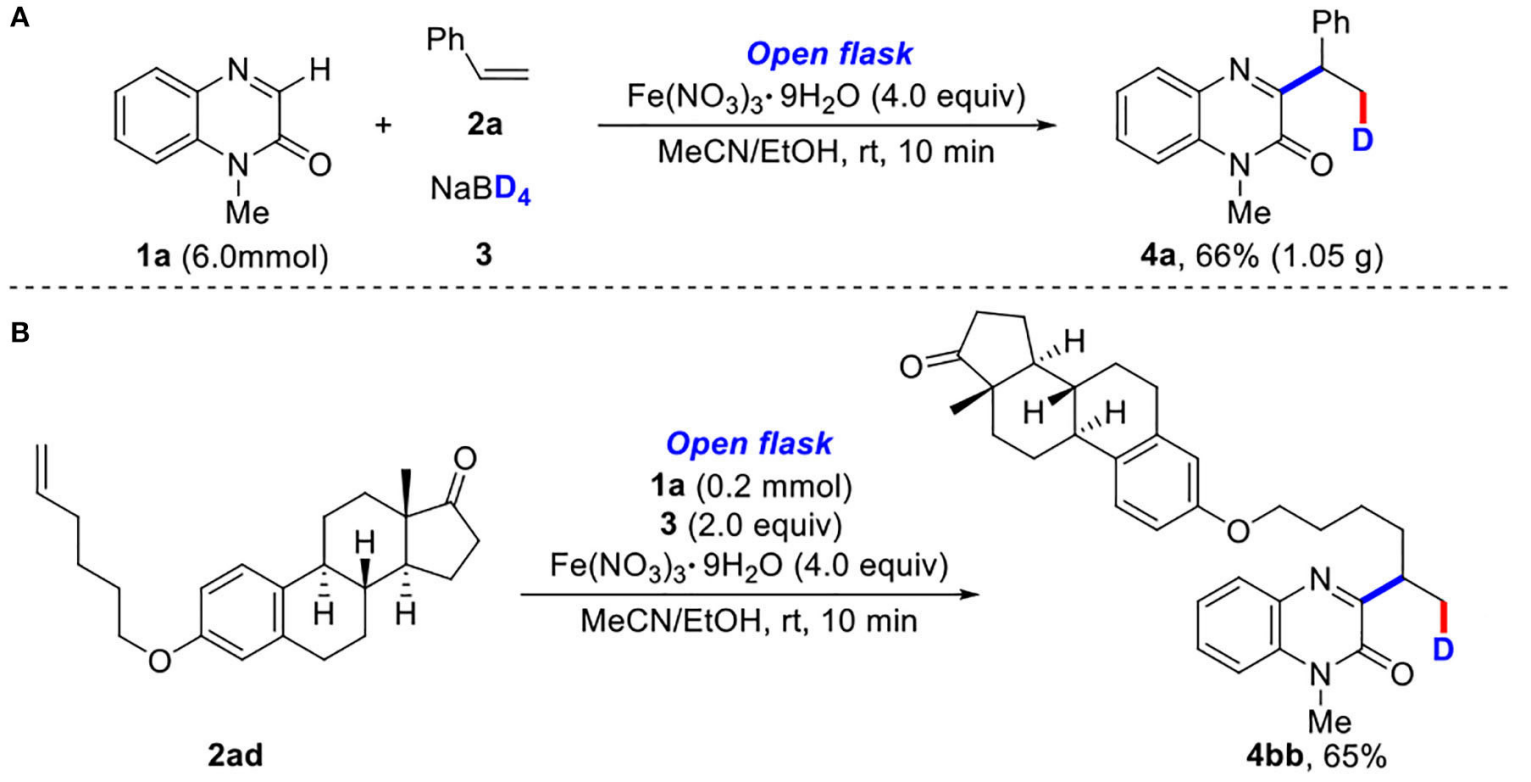
**(A,B)** Gram-scale synthesis and modification of estrone derivative.

To demonstrate the synthetic utility of our method, a gram-scale experiment was performed to synthesize 1-methyl-3-(1-phenylethyl-2-d)quinoxalin-2(1*H*)-one (**4a**) in 66% yield (Scheme 2A). It was worth mentioning that the modification of estrone derivative further demonstrated its synthetic utility (Scheme 2B).

**Scheme 3:**
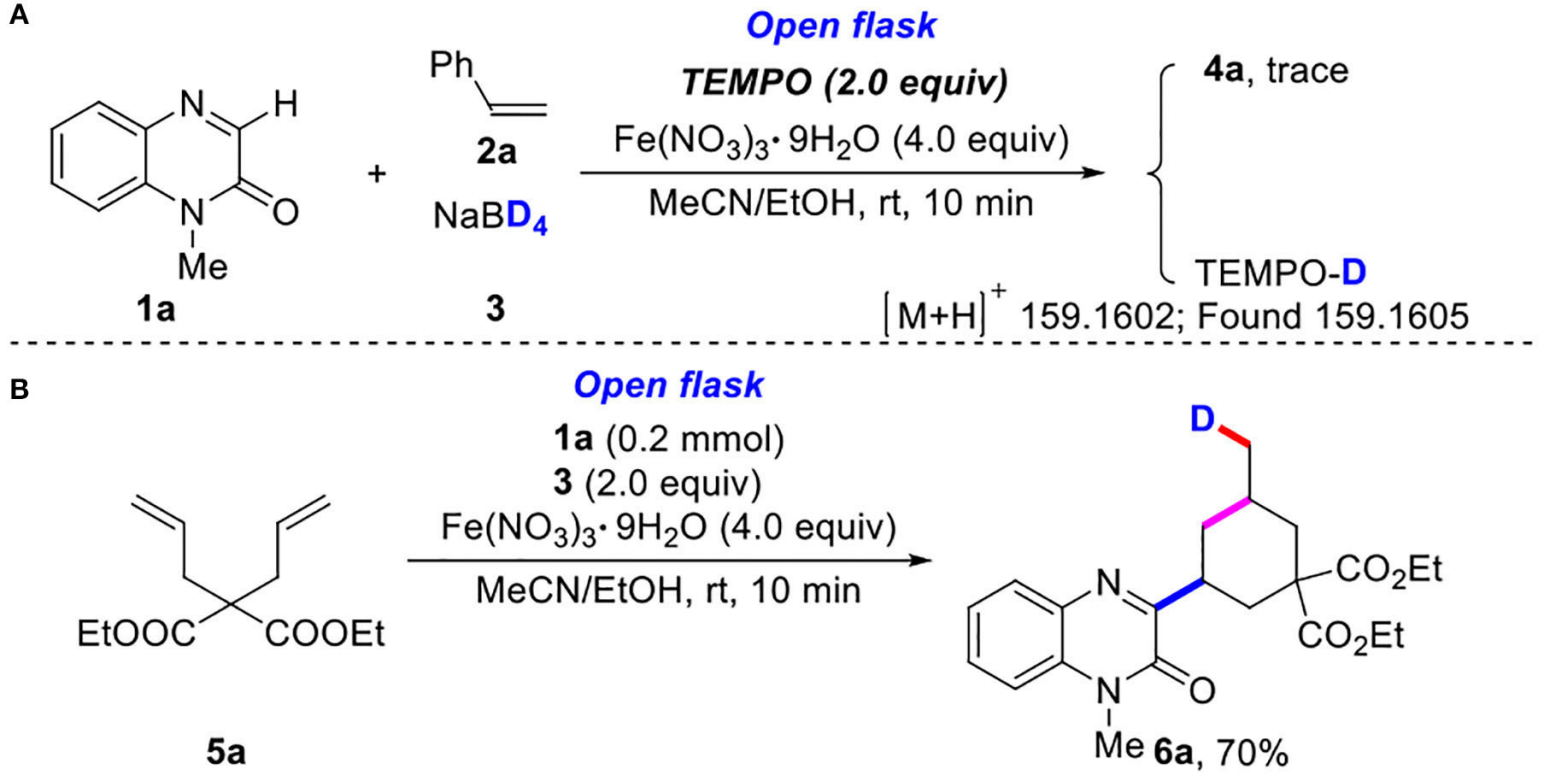
Control experiments. **(A)** The experiment of deuterium radical inhibition. **(B)** The experiment of deuterium radical addition reaction.

To understand the reaction mechanism, the preliminary mechanistic studies was proceeded (Scheme 3). When 2.0 equiv of TEMPO (2,2,6,6-tetramethyl-piperidin-1-oxyl) were used as radical inhibitor, the reaction was completely inhibited (Scheme 3A). In addition, the transformation of 1-methylquinoxalin-2(1*H*)-one (**1a**) and diethyl 2,2-diallylmalonate (**5a**) with NaBD_4_ (**3**) performed to give product **6a** in 70% yield (Scheme 3B). All these results clearly implied that a radical pathway was responsible for the three-component reaction.

**Scheme 4:**
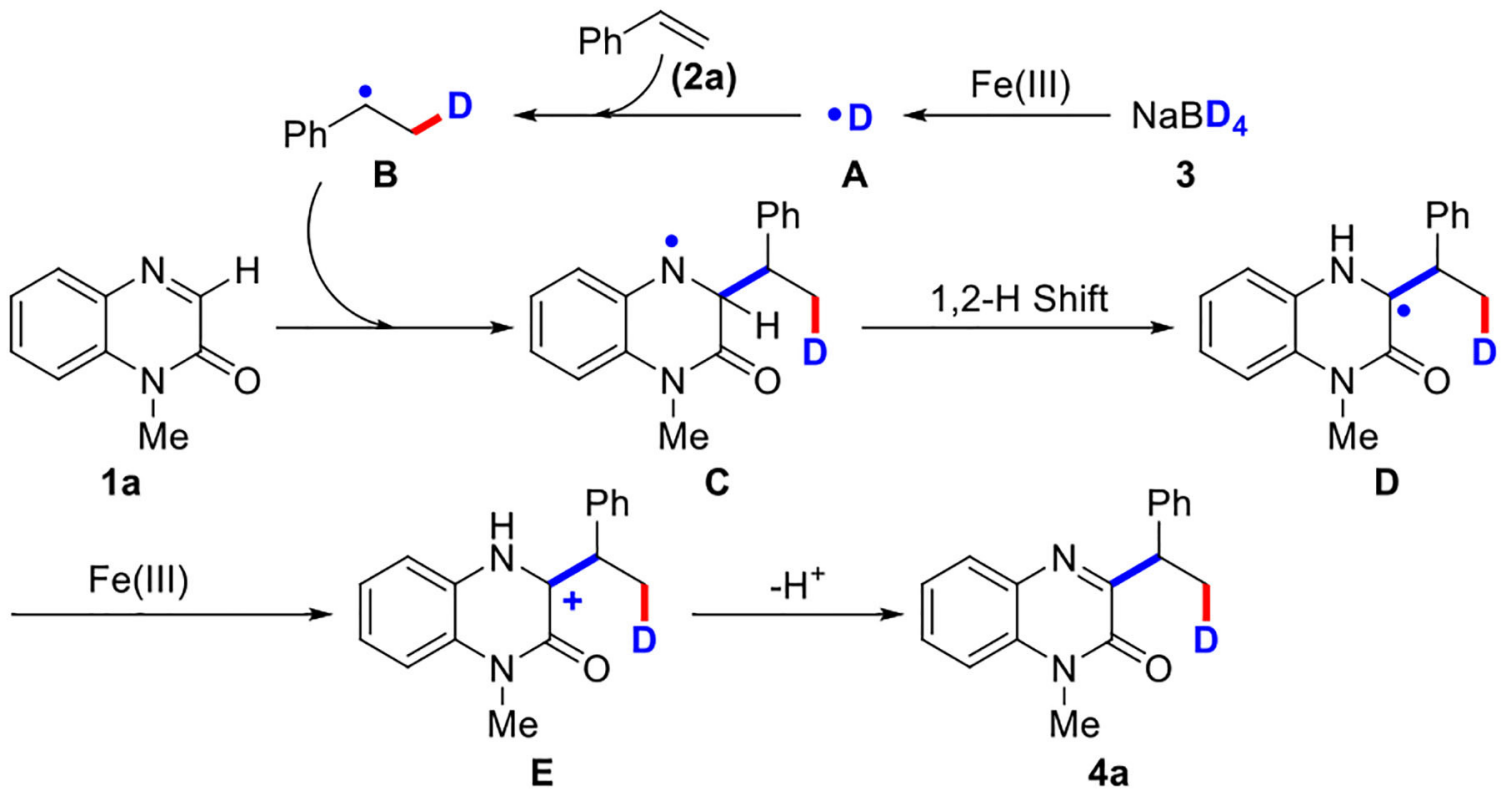
Plausible mechanism.

Based on the above experimental results and previous reports (Yi et al., 2017; Yan et al., 2019; Shen et al., 2020), a probable radical mechanism for the three-component reaction was proposed (Scheme 4). First, deuterium radical (**A**) was generated from NaBD_4_ in the presence of Fe(III). Second, the generated deuterium radical (**A**) attacked olefin **2a** to afford alkyl radical (**B**). Third, alkyl radical (**B**) then attacked quinoxalinone **1a** to give nitrogen radical (**C**), which underwent a 1,2-hydrogen shift process to produce carbon radical (**D**). After the generation of carbon cation (**E**) from carbon radical (**D**) by the oxidation of Fe(III), the final product **4a** was obtained through a deprotonation process.

## Experimental section

### General Information

All reagents and deuterated solvents were commercially available and used without further purification. All products were separated by silica gel (200–300 mesh) column chromatography with petroleum ether (PE) (60–90°C) and ethyl acetate (EA). ^1^H, ^13^C, and ^19^F NMR spectra were recorded on a Bruker Advance 500 spectrometer at ambient temperature with CDCl_3_ as solvent and tetramethylsilane (TMS) as the internal standard. Melting points were determined on an X-5 Data microscopic melting point apparatus. Analytical thin layer chromatography (TLC) was performed on Merk precoated TLC (silica gel 60 F254) plates. Compounds for high-resolution mass spectrometry (HRMS) were analyzed by positive mode electrospray ionization (ESI) using Agilent 6530 QTOF mass spectrometer.

### Typical Reaction Procedure for the Cascade Reaction of Quinoxalinones With Unactivated Alkenes and NaBD_4_

A mixture of quinoxalinones (**1**) (0.2 mmol), olefins (**2**) (2.0 equiv), Fe(NO_3_)_3_•9H_2_O (4.0 equiv), and MeCN/EtOH (4.0 ml, *v*/*v* = 1:1) in a 15-ml tube was stirred at room temperature for 5 min to make all the components dissolved. Then, NaBD_4_ (2.0 equiv) was slowly added. The resulting mixture was stirred for another 5 min. After the completion (as indicated by TLC), the reaction mixture was quenched with aqueous NH_3_•H_2_O (2 ml) and extracted with EtOAc (5 ml × 3). The collected organic layer was washed with brine and dried with MgSO_4_. Finally, the organic solvent was removed under reduced pressure, and the obtained residue was purified by silica gel column chromatography (200–300 mesh silica gel, PE/EA = 3:1).

### Gram-Scale Synthesis of 1-methyl-3-(1-phenylethyl-2-*d*)quinoxalin-2(1*H*)-one

A mixture of quinoxalinones (**1**) (6.0 mmol), olefins (**2**) (2.0 equiv), Fe(NO_3_)_3_•9H_2_O (4.0 equiv), and MeCN/EtOH (100 ml, *v*/*v* = 1:1) in a 250-ml flask was stirred at room temperature for 5 min to make all the components dissolved. Then, NaBD_4_ (2.0 equiv) was slowly added. The resulting mixture was stirred for another 5 min. After the completion (as indicated by TLC), the reaction mixture was quenched with aqueous NH_3_•H_2_O (50 ml) and extracted with EtOAc (50 ml × 3). The collected organic layer was washed with brine and dried with MgSO_4_. Finally, the organic solvent was removed under reduced pressure, and the obtained residue was purified by silica gel column chromatography (200–300 mesh silica gel, PE/EA = 3:1) to provide product **4a** in 66% yield (1.05 g).

## Conclusion

In conclusion, a rapid three-component deuteration of quinoxalinones with olefins and NaBD_4_ was reported for the first time. Quinoxalinones or olefins bearing various functional groups could undergo the reaction smoothly, producing the target products in moderate to good yields. This transformation gave a novel and efficient method for the synthesis of previously unknown deuterated quinoxalinones.

## Data Availability Statement

All datasets presented in this study are included in the article/Supplementary Material.

## Author Contributions

WL and PZ contributed conception and design of the study. HC wrote sections of the manuscript. All authors contributed to manuscript revision, read, and approved the submitted version.

## Conflict of Interest

The authors declare that the research was conducted in the absence of any commercial or financial relationships that could be construed as a potential conflict of interest.
