# Supplementary material for: Iron(III)-Mediated Rapid Radical-Type Three-Component Deuteration of Quinoxalinones With Olefins and NaBD4
Source: Front Chem. 2020 Aug 4;8:606. doi: 10.3389/fchem.2020.00606 (PMC7417774; doi:10.3389/fchem.2020.00606)

Supplementary Material

**Iron (III)-Mediated Rapid Radical-Type Three-Component Deuteration of Quinoxalinones with Olefins and NaBD_4_**

Wanmei Li,* Heng Cai, Lin Huang, Lei He, Yilan Zhang, Jun Xu, and Pengfei Zhang

College of Material Chemistry and Chemical Engineering, Hangzhou Normal University, Hangzhou 311121, China.

General Information 2

1. Experimental Section 2

2. Characterization of Products 4

3. X-ray Crystal Data for **4al** 19

4. Copies of ^1^H, ^13^C and ^19^F NMR Spectra 20

**General Information**

All reagents and deuterated solvents were commercially available and used without further purification. All products were separated by silica gel (200-300 mesh) column chromatography with petroleum ether (PE) (60-90°C) and ethyl acetate (EA). ^1^H, ^13^C and ^19^F NMR spectra were recorded on a Bruker Advance 500 spectrometer at ambient temperature with CDCl_3_ as solvent and tetramethylsilane (TMS) as the internal standard. Melting points were determined on an X-5 Data microscopic melting point apparatus. Analytical thin layer chromatography (TLC) was performed on Merk precoated TLC (silica gel 60 F254) plates. Compounds for HRMS were analyzed by positive mode electrospray ionization (ESI) using Agilent 6530 QTOF mass spectrometer.

1. **Experimental Section**

**1.1 General procedure for the synthesis of products 4**

A mixture of quinoxalinones (1) (0.2 mmol), olefins (2) (2.0 equiv), Fe(NO3)3•9H_2_O (4.0 equiv) and MeCN/EtOH (4.0 mL, v/v = 1:1) in a 15 mL tube was stirred at room temperature for 5 min to make all the components dissolved. Then, NaBD_4_ (2.0 equiv) was slowly added. The resulting mixture was stirred for another 5 min. After the completion (as indicated by TLC), the reaction mixture was quenched with aqueous NH_3_•H_2_O (2 mL), and extracted with EtOAc (5 mL x 3). The collected organic layer was washed with brine and dried with MgSO_4_. Finally, the organic solvent was removed under reduced pressure, and the obtained residue was purified by silica gel column chromatography (200-300 mesh silica gel, PE/EA = 3:1).

**1.2 General procedure for gram-scale synthesis of product 4a**

A mixture of quinoxalinones (**1a**) (6.0 mmol), olefins (**2a**) (2.0 equiv), Fe(NO_3_)_3_•9H_2_O (4.0 equiv) and MeCN/EtOH (100 mL, v/v = 1:1) in a 250 mL flask was stirred at room temperature for 5 min to make all the components dissolved. Then, NaBD_4_ (2.0 equiv) was slowly added. The resulting mixture was stirred for another 5 min. After the completion (as indicated by TLC), the reaction mixture was quenched with aqueous NH_3_•H_2_O (50 mL), and extracted with EtOAc (50 mL x 3). The collected organic layer was washed with brine and dried with MgSO_4_. Finally, the organic solvent was removed under reduced pressure, and the obtained residue was purified by silica gel column chromatography (200-300 mesh silica gel, PE/EA = 3:1) to provide product **4a** in 66% yield (1.05 g).

**1.3 A series of substrates besides quinoxalinones.**

1. **Characterization of Products**

**1-Methyl-3-(1-phenylethyl-2-d)quinoxalin-2(1H)-one (4a)**

Obtained as a white solid (39 mg, 73% yield); M.p. 173-174 ^o^C. ^1^H NMR (500 MHz, CDCl_3_) *δ* 7.92 (dd, *J* = 8.0, 1.4 Hz, 1H), 7.50 (ddd, *J* = 8.5, 7.4, 1.5 Hz, 1H), 7.45 – 7.42 (m, 2H), 7.33 (t, *J* = 7.1 Hz, 1H), 7.26 (d, *J* = 9.9 Hz, 3H), 7.19 – 7.15 (m, 1H), 4.82 (t, *J* = 7.1 Hz, 1H), 3.61 (s, 3H), 1.67 (d, *J* = 7.1 Hz, 2H); ^13^C NMR (126 MHz, CDCl_3_) *δ* 161.89, 154.45, 143.13, 133.09, 132.72, 130.13, 129.73, 128.38, 128.15, 126.53, 123.46, 113.50, 41.81, 29.11, 19.40, (t, *J* = 20.1 Hz); HRMS (ESI+): Calculated for C_17_H_15_DN_2_O: [M+H]^+^ 266.1398, Found 266.1398.

**1-Ethyl-3-(1-phenylethyl-2-d)quinoxalin-2(1H)-one (4b)**

Obtained as a colourless liquid (43 mg, 77% yield); ^1^H NMR (500 MHz, CDCl_3_) *δ* 7.93 (d, *J* = 8.0 Hz, 1H), 7.50 (t, *J* = 7.8 Hz, 1H), 7.45 (d, *J* = 7.6 Hz, 2H), 7.32 (t, *J* = 7.6 Hz, 1H), 7.26 (dd, *J* = 11.9, 4.3 Hz, 3H), 7.17 (t, *J* = 7.3 Hz, 1H), 4.84 (t, *J* = 7.1 Hz, 1H), 4.24 (ddd, *J* = 63.1, 13.9, 7.0 Hz, 2H), 1.67 (d, *J* = 7.1 Hz, 2H), 1.31 (t, *J* = 7.2 Hz, 3H); ^13^C NMR (126 MHz, CDCl_3_) *δ* 161.95, 153.93, 143.21, 133.02, 132.00, 130.39, 129.70, 128.37, 128.14, 126.47, 123.25, 113.35, 41.60, 37.37, 19.47, (t, *J* = 20.1 Hz), 12.39; HRMS (ESI+): Calculated for C_18_H_17_DN_2_O: [M+H]^+^ 280.1555, Found 280.1559.

**1-Butyl-3-(1-phenylethyl-2-d)quinoxalin-2(1H)-one (4c)**

Obtained as a colourless liquid (46 mg, 75% yield); ^1^H NMR (500 MHz, CDCl_3_) *δ* 7.92 (dd, *J* = 8.0, 1.4 Hz, 1H), 7.49 (ddd, *J* = 8.6, 7.4, 1.5 Hz, 1H), 7.44 (dd, *J* = 8.1, 1.0 Hz, 2H), 7.33 – 7.29 (m, 1H), 7.28 – 7.24 (m, 3H), 7.17 (ddd, *J* = 8.5, 2.3, 1.2 Hz, 1H), 4.83 (t, *J* = 7.1 Hz, 1H), 4.28 – 4.02 (m, 2H), 1.67 (dd, *J* = 8.7, 4.7 Hz, 4H), 1.42 (dd, *J* = 15.1, 7.5 Hz, 2H), 0.95 (t, *J* = 7.4 Hz, 3H); ^13^C NMR (126 MHz, CDCl_3_) *δ* 161.91, 154.15, 143.24, 133.00, 132.27, 130.36, 129.64, 128.37, 128.12, 126.46, 123.23, 113.51, 42.23, 41.67, 29.26, 20.30, 19.47, (t, *J* = 20.1 Hz), 13.78; HRMS (ESI+): Calculated for C_20_H_21_DN_2_O: [M+H]^+^ 308.1868, Found 308.1874.

**1-(Cyclopropylmethyl)-3-(1-phenylethyl-2-d)quinoxalin-2(1H)-one (4d)**

Obtained as a colourless liquid (43 mg, 70% yield); ^1^H NMR (500 MHz, CDCl3) δ 7.93 (dd, J = 8.0, 1.1 Hz, 1H), 7.52 – 7.48 (m, 1H), 7.43 (d, J = 7.6 Hz, 2H), 7.38 (d, J = 8.3 Hz, 1H), 7.33 (t, J = 7.6 Hz, 1H), 7.29 – 7.25 (m, 2H), 7.17 (t, J = 7.3 Hz, 1H), 4.83 (t, J = 7.1 Hz, 1H), 4.12 (ddd, J = 20.5, 14.3, 7.0 Hz, 2H), 1.67 (d, J = 7.1 Hz, 2H), 1.25 – 1.19 (m, 1H), 0.57 – 0.41 (m, 4H); ^13^C NMR (126 MHz, CDCl3) δ 162.11, 154.44, 143.25, 132.93, 132.50, 130.34, 129.59, 128.35, 128.10, 126.45, 123.24, 113.79, 46.13, 41.72, 19.43 (t, *J* = 20.1 Hz), 9.61, 4.30, 3.91; HRMS (ESI+): Calculated for C_20_H_19_DN_2_O: [M+H^]+^ 306.1711, Found 306.1717.

**tert-Butyl 2-(2-oxo-3-(1-phenylethyl-2-d)quinoxalin-1(2H)-yl)acetate (4e)**

Obtained as a colourless liquid (45 mg, 71% yield); ^1^H NMR (500 MHz, CDCl_3_) *δ* 7.93 (dd, *J* = 8.0, 1.1 Hz, 1H), 7.50 – 7.46 (m, 1H), 7.41 (d, *J* = 7.3 Hz, 2H), 7.36 – 7.32 (m, 1H), 7.27 – 7.24 (m, 2H), 7.17 (t, *J* = 7.4 Hz, 1H), 7.02 (d, *J* = 8.3 Hz, 1H), 4.98 (d, *J* = 17.2 Hz, 1H), 4.81 (t, *J* = 7.1 Hz, 1H), 4.73 (d, *J* = 17.2 Hz, 1H), 1.67 (d, *J* = 7.1 Hz, 2H), 1.39 (s, 9H); ^13^C NMR (126 MHz, CDCl_3_) *δ* 166.17, 161.72, 153.98, 143.02, 132.73, 132.28, 130.41, 129.78, 128.37, 128.07, 126.49, 123.64, 112.97, 83.01, 44.27, 41.85, 27.90, 19.38 (t, *J* = 20.1 Hz); HRMS (ESI+): Calculated for C_22_H_23_DN_2_O_3_: [M+H]^+^ 366.1923, Found 366.1925.

**1-Benzyl-3-(1-phenylethyl-2-d)quinoxalin-2(1H)-one (4f)**

Obtained as a colourless liquid (42 mg, 61% yield); ^1^H NMR (500 MHz, CDCl_3_) *δ* 7.93 (dd, *J* = 7.9, 1.1 Hz, 1H), 7.46 (d, *J* = 7.3 Hz, 2H), 7.40 – 7.36 (m, 1H), 7.34 – 7.25 (m, 5H), 7.23 – 7.18 (m, 3H), 7.15 (d, *J* = 7.1 Hz, 2H), 5.42 (dd, *J* = 117.0, 15.7 Hz, 2H), 4.89 (t, *J* = 7.0 Hz, 1H), 1.70 (d, *J* = 7.1 Hz, 2H); ^13^C NMR (126 MHz, CDCl_3_) *δ* 162.05, 154.53, 143.16, 135.32, 132.98, 132.40, 130.24, 129.69, 128.86, 128.40, 128.11, 127.58, 126.82, 126.52, 123.50, 114.29, 45.93, 41.82, 19.46 (t, *J* = 20.1 Hz); HRMS (ESI+): Calculated for C_23_H_19_DN_2_O: [M+H]^+^ 342.1711, Found 342.1717.

**1-(4-Methylbenzyl)-3-(1-phenylethyl-2-d)quinoxalin-2(1H)-one (4g)**

Obtained as a colourless liquid (55 mg, 78% yield); ^1^H NMR (500 MHz, CDCl_3_) δ 7.91 (dd, *J* = 8.0, 1.4 Hz, 1H), 7.47 – 7.44 (m, 2H), 7.35 (dd, *J* = 8.3, 1.3 Hz, 1H), 7.30 – 7.25 (m, 3H), 7.22 – 7.17 (m, 2H), 7.05 (s, 4H), 5.36 (dd, *J* = 119.8, 16.3 Hz, 2H), 4.89 (t, *J* = 7.1 Hz, 1H), 2.26 (s, 3H), 1.69 (d, *J* = 7.1 Hz, 2H); ^13^C NMR (126 MHz, CDCl_3_) *δ* 162.08, 154.56, 143.22, 137.33, 132.98, 132.46, 132.35, 130.22, 129.70, 129.55, 128.43, 128.15, 126.90, 126.55, 123.47, 114.34, 45.72, 41.83, 21.09, 19.50 (t, *J* = 20.1 Hz); HRMS (ESI+): Calculated for C_24_H_21_DN_2_O: [M+H]^+^ 356.1868, Found 356.1868.

**1-(4-Fluorobenzyl)-3-(1-phenylethyl-2-d)quinoxalin-2(1H)-one (4h)**

Obtained as a colourless liquid (43 mg, 60% yield); ^1^H NMR (500 MHz, CDCl_3_) *δ* 7.93 (d, *J* = 7.8 Hz, 1H), 7.45 (d, *J* = 7.5 Hz, 2H), 7.40 (t, *J* = 7.5 Hz, 1H), 7.30 (dt, *J* = 11.8, 7.7 Hz, 3H), 7.19 (t, *J* = 8.4 Hz, 2H), 7.14 (dd, *J* = 8.3, 5.3 Hz, 2H), 6.95 (t, *J* = 8.5 Hz, 2H), 5.37 (dd, *J* = 104.5, 15.4 Hz, 2H), 4.87 (t, *J* = 7.0 Hz, 1H), 1.69 (d, *J* = 7.1 Hz, 2H); ^13^C NMR (126 MHz, CDCl_3_) *δ* 162.13 (t, *J* = 109.6 Hz), 154.47, 143.11, 133.01, 132.26, 131.09, (d, *J* = 2.5 Hz), 130.39, 129.76, 128.70, (d, *J* = 7.6 Hz) 128.44, 128.11, 126.58, 123.64, 115.92, 115.74, 114.06, 45.29, 41.86, 19.46, (t, *J* = 20.1 Hz); ^19^F NMR (471 MHz, CDCl_3_) δ -114.96; HRMS (ESI+): Calculated for C_23_H_18_DFN_2_O: [M+H]^+^ 360.1617, Found 360.1621.

**1-(4-Chlorobenzyl)-3-(1-phenylethyl-2-d)quinoxalin-2(1H)-one (4i)**

Obtained as a colourless liquid (45 mg, 61% yield); ^1^H NMR (500 MHz, CDCl_3_) *δ* 7.94 (dd, *J* = 8.0, 1.3 Hz, 1H), 7.44 (d, *J* = 7.3 Hz, 2H), 7.42 – 7.38 (m, 1H), 7.33 – 7.27 (m, 3H), 7.23 (d, *J* = 8.5 Hz, 2H), 7.19 (t, *J* = 7.4 Hz, 1H), 7.16 – 7.13 (m, 1H), 7.09 (d, *J* = 8.4 Hz, 2H), 5.37 (dd, *J* = 104.8, 15.7 Hz, 2H), 4.87 (t, *J* = 7.1 Hz, 1H), 1.70 (d, *J* = 7.1 Hz, 2H); ^13^C NMR (126 MHz, CDCl_3_) *δ* 162.01, 154.43, 143.06, 133.84, 133.49, 132.98, 132.18, 130.39, 129.77, 129.06, 128.42, 128.30, 128.09, 126.57, 123.67, 114.00, 45.30, 41.86, 19.43 (t, *J* = 20.1 Hz); HRMS (ESI+): Calculated for C_23_H_18_DClN_2_O: [M+H]^+^ 376.1322, Found 376.1329.

**1-(4-Bromobenzyl)-3-(1-phenylethyl-2-d)quinoxalin-2(1H)-one (4j)**

Obtained as a colourless liquid (59 mg, 70% yield); ^1^H NMR (500 MHz, CDCl_3_) *δ* 8.07 (dd, *J* = 8.1, 1.3 Hz, 1H), 7.77 (dd, *J* = 8.2, 1.2 Hz, 1H), 7.62 – 7.58 (m, 1H), 7.57 – 7.52 (m, 1H), 7.43 – 7.38 (m, 2H), 7.25 – 7.22 (m, 4H), 7.19 (dd, *J* = 8.0, 4.1 Hz, 1H), 7.02 (d, *J* = 8.4 Hz, 2H), 5.36 (dd, *J* = 61.4, 12.6 Hz, 2H), 4.64 (t, *J* = 7.1 Hz, 1H), 1.75 (d, *J* = 7.1 Hz, 2H); ^13^C NMR (126 MHz, CDCl_3_) *δ* 155.05, 152.19, 143.83, 139.58, 138.70, 135.58, 131.47, 129.68, 129.17, 128.82, 128.39, 128.05, 126.67, 126.51, 126.39, 121.82, 67.18, 42.13, 19.65 (t, *J* = 20.1 Hz); HRMS (ESI+): Calculated for C_23_H_18_DBrN_2_O: [M+H]^+^ 420.0817, Found 420.0811.

**1-(3-Methylbenzyl)-3-(1-phenylethyl-2-d)quinoxalin-2(1H)-one (4k)**

Obtained as a colourless liquid (52 mg, 73% yield); ^1^H NMR (500 MHz, CDCl_3_) *δ* 7.92 (d, *J* = 7.9 Hz, 1H), 7.45 (d, *J* = 7.8 Hz, 2H), 7.37 (dd, *J* = 8.2, 7.4 Hz, 1H), 7.28 (t, *J* = 7.6 Hz, 3H), 7.22 – 7.16 (m, 2H), 7.14 (t, *J* = 7.6 Hz, 1H), 7.02 (d, *J* = 7.5 Hz, 1H), 6.97 – 6.83 (m, 2H), 5.37 (dd, *J* = 108.0, 15.6 Hz, 2H), 4.90 (t, *J* = 7.1 Hz, 1H), 2.24 (s, 3H), 1.70 (d, *J* = 7.1 Hz, 2H); ^13^C NMR (126 MHz, CDCl_3_) *δ* 162.06, 154.57, 143.23, 138.66, 135.28, 132.99, 132.48, 130.22, 129.71, 128.73, 128.42, 128.40, 128.13, 127.43, 126.54, 123.90, 123.48, 114.36, 45.93, 41.86, 21.42, 19.44 (t, *J* = 20.1 Hz); HRMS (ESI+): Calculated for C_24_H_21_DN_2_O: [M+H]^+^ 356.1868, Found 356.1868.

**1-(3-Chlorobenzyl)-3-(1-phenylethyl-2-d)quinoxalin-2(1H)-one (4l)**

Obtained as a colourless liquid (52 mg, 70% yield); ^1^H NMR (500 MHz, CDCl_3_) *δ* 7.94 (dd, *J* = 8.0, 1.4 Hz, 1H), 7.50 – 7.43 (m, 2H), 7.42 – 7.38 (m, 1H), 7.34 – 7.27 (m, 3H), 7.19 (ddd, *J* = 12.0, 5.3, 2.6 Hz, 3H), 7.13 (d, *J* = 7.6 Hz, 2H), 7.01 (d, *J* = 6.5 Hz, 1H), 5.37 (dd, *J* = 125.5, 15.8 Hz, 2H), 4.87 (t, *J* = 7.1 Hz, 1H), 1.70 (d, *J* = 7.1 Hz, 2H); ^13^C NMR (126 MHz, CDCl_3_) *δ* 162.02, 154.43, 143.09, 137.41, 134.84, 132.99, 132.20, 130.42, 130.19, 129.85, 128.46, 128.08, 127.95, 126.96, 126.60, 125.00, 123.73, 114.01, 45.41, 41.91, 19.44 (t, *J* = 20.1 Hz); HRMS (ESI+): Calculated for C_23_H_18_DClN_2_O: [M+H]^+^ 376.1322, Found 376.1323.

**1-(3-Bromobenzyl)-3-(1-phenylethyl-2-d)quinoxalin-2(1H)-one (4m)**

Obtained as a colourless liquid (58 mg, 69% yield); ^1^H NMR (500 MHz, CDCl_3_) *δ* 8.02 – 7.89 (m, 1H), 7.44 (d, *J* = 7.4 Hz, 2H), 7.42 – 7.38 (m, 1H), 7.36 (d, *J* = 8.0 Hz, 1H), 7.34 – 7.26 (m, 4H), 7.19 (t, *J* = 7.4 Hz, 1H), 7.12 (t, *J* = 7.9 Hz, 2H), 7.05 (d, *J* = 7.7 Hz, 1H), 5.36 (dd, *J* = 125.1, 15.8 Hz, 2H), 4.87 (t, *J* = 7.1 Hz, 1H), 1.70 (d, *J* = 7.1 Hz, 2H); ^13^C NMR (126 MHz, CDCl_3_) *δ* 162.02, 154.43, 143.09, 137.67, 132.98, 132.19, 130.89, 130.46, 130.42, 129.86, 128.47, 128.08, 126.60, 125.46, 123.73, 123.01, 114.00, 45.35, 41.91, 19.44 (t, *J* = 20.1 Hz); HRMS (ESI+): Calculated for C_23_H_18_DBrN_2_O: [M+H]^+^ 420.0817, Found 420.0811.

**1-(2-Fluorobenzyl)-3-(1-phenylethyl-2-d)quinoxalin-2(1H)-one (4n)**

Obtained as a colourless liquid (44 mg, 61% yield); ^1^H NMR (500 MHz, CDCl_3_) δ 7.92 (dd, *J* = 7.9, 1.2 Hz, 1H), 7.45 (d, *J* = 7.3 Hz, 2H), 7.41 – 7.36 (m, 1H), 7.28 (td, *J* = 7.5, 4.0 Hz, 3H), 7.21 – 7.15 (m, 3H), 7.08 – 7.03 (m, 1H), 6.93 (t, *J* = 7.5 Hz, 1H), 6.86 (t, *J* = 7.2 Hz, 1H), 5.46 (dd, *J* = 85.1, 16.1 Hz, 2H), 4.88 (t, *J* = 7.0 Hz, 1H), 1.70 (d, *J* = 7.1 Hz, 2H); ^13^C NMR (126 MHz, CDCl_3_) *δ* 161.95, 161.25, 159.30, 154.66, 137.5 (d, *J* = 13.9 Hz), 132.11, 130.29, 129.89, 129.31 (d, *J* = 8.8 Hz), 128.41, 128.37, 128.09, 126.55, 124.63 (d, *J* = 3.8 Hz), 123.67, 122.37 (d, *J* = 13.9 Hz), 115.54, 115.37, 113.88 (d, *J* = 1.3 Hz), 41.86, 39.32 (d, *J* = 5.0 Hz), 19.42 (t, *J* = 20.1 Hz); ^19^F NMR (471 MHz, CDCl_3_) *δ* -118.48; HRMS (ESI+): Calculated for C_23_H_18_DFN_2_O: [M+H]^+^ 360.1617, Found 360.1620.

**1-(2-Bromobenzyl)-3-(1-phenylethyl-2-d)quinoxalin-2(1H)-one (4o)**

Obtained as a white solid (50 mg, 60% yield); M.p. 120-121 ^o^C. ^1^H NMR (500 MHz, CDCl_3_) *δ* 8.08 (dd, *J* = 8.1, 1.2 Hz, 1H), 7.81 (dd, *J* = 8.2, 1.0 Hz, 1H), 7.63 – 7.59 (m, 1H), 7.59 – 7.54 (m, 2H), 7.28 (d, *J* = 7.2 Hz, 2H), 7.21 (t, *J* = 7.3 Hz, 2H), 7.19 – 7.11 (m, 3H), 7.07 (dd, *J* = 7.2, 1.9 Hz, 1H), 5.54 (dd, *J* = 45.5, 13.3 Hz, 2H), 4.72 (t, *J* = 7.1 Hz, 1H), 1.77 (d, *J* = 7.1 Hz, 2H); ^13^C NMR (126 MHz, CDCl_3_) *δ* 154.95, 152.20, 143.59, 139.61, 138.60, 135.95, 132.64, 129.66, 129.32, 129.17, 128.66, 128.35, 128.12, 127.34, 126.82, 126.53, 126.38, 123.19, 67.39, 41.95, 19.45 (t, *J* = 20.1 Hz); HRMS (ESI+): Calculated for C_23_H_18_DBrN_2_O: [M+H]^+^ 420.0817, Found 420.0810.

**1-(2,6-Difluorobenzyl)-3-(1-phenylethyl-2-d)quinoxalin-2(1H)-one (4p)**

Obtained as a white solid (45 mg, 60% yield); M.p. 122-123 ^o^C. ^1^H NMR (500 MHz, CDCl_3_) δ 7.89 (dd, *J* = 7.9, 1.1 Hz, 1H), 7.44 (d, *J* = 7.3 Hz, 2H), 7.41 – 7.36 (m, 1H), 7.31 – 7.25 (m, 3H), 7.19 (dd, *J* = 15.4, 7.8 Hz, 3H), 6.81 (t, *J* = 8.2 Hz, 2H), 5.55 (dd, *J* = 92.9, 15.7 Hz, 2H), 4.87 (t, *J* = 7.1 Hz, 1H), 1.68 (d, *J* = 7.1 Hz, 2H); ^13^C NMR (126 MHz, CDCl_3_) *δ* 162.24 (d, *J* = 7.56 Hz), 161.83, 160.26 (d, *J* = 7.6 Hz), 154.35, 143.13, 133.06, 132.02, 130.35, 129.65, 128.35, 128.07, 126.47, 123.43, 113.43, 111.85 (d, *J* = 5.0 Hz), 111.66 (t, *J* = 6.3 Hz), 41.89, 34.90, 19.38 (t, *J* = 20.1 Hz); ^19^F NMR (471 MHz, CDCl_3_) *δ* -113.84; HRMS (ESI+): Calculated for C_23_H_17_DF_2_N_2_O: [M+H]^+^ 378.1523, Found 378.1523.

**1-(2,6-Dichlorobenzyl)-3-(1-phenylethyl-2-d)quinoxalin-2(1H)-one (4q)**

Obtained as a colourless liquid (48 mg, 59% yield); ^1^H NMR (500 MHz, CDCl_3_) δ 7.89 (dd, *J* = 7.9, 1.5 Hz, 1H), 7.42 (d, *J* = 7.2 Hz, 2H), 7.33 – 7.27 (m, 2H), 7.27 – 7.23 (m, 2H), 7.21 (dd, *J* = 7.4, 4.6 Hz, 2H), 7.18 (t, *J* = 7.4 Hz, 1H), 7.11 – 7.07 (m, 1H), 7.04 (dd, *J* = 8.3, 1.0 Hz, 1H), 5.74 (dd, *J* = 109.3, 16.0 Hz, 2H), 4.86 (t, *J* = 7.1 Hz, 1H), 1.69 (d, *J* = 7.1 Hz, 2H); ^13^C NMR (126 MHz, CDCl_3_) *δ* 161.42, 155.14, 142.70, 135.46, 133.21, 131.98, 130.98, 130.28, 129.39, 129.26, 128.30, 128.22, 126.49, 123.35, 114.22, 42.10, 41.73, 19.08 (t, *J* = 20.1 Hz); HRMS (ESI+): Calculated for C_23_H_17_DCl_2_N_2_O: [M+H]^+^ 410.0932, Found 410.0932.

**6-Methoxy-1-methyl-3-(1-phenylethyl-2-d)quinoxalin-2(1H)-one (4r)**

Obtained as a white solid (35 mg, 60% yield); M.p. 137-138 ^o^C. ^1^H NMR (500 MHz, CDCl_3_) *δ* 7.83 (d, *J* = 8.8 Hz, 1H), 7.42 (d, *J* = 7.3 Hz, 2H), 7.29 – 7.26 (m, 2H), 7.17 (t, *J* = 7.3 Hz, 1H), 6.92 (dd, *J* = 8.7, 2.2 Hz, 1H), 6.67 (d, *J* = 2.4 Hz, 1H), 4.77 (t, *J* = 7.0 Hz, 1H), 3.91 (s, 3H), 3.59 (s, 3H), 1.65 (d, *J* = 7.1 Hz, 2H); ^13^C NMR (126 MHz, CDCl_3_) *δ* 160.82, 158.47, 154.73, 143.53, 134.52, 131.38, 128.33, 128.07, 127.67, 126.40, 110.37, 97.89, 55.79, 41.60, 29.14, 19.42 (t, *J* = 20.1 Hz); HRMS (ESI+): Calculated for C_18_H_17_DN_2_O_2_: [M+H]^+^ 296.1504, Found 296.1501.

**6-Chloro-1-methyl-3-(1-phenylethyl-2-d)quinoxalin-2(1H)-one (4s)**

Obtained as a white solid (36 mg, 61% yield); M.p. 156-157 ^o^C. ^1^H NMR (500 MHz, CDCl_3_) *δ* 7.84 (d, *J* = 8.5 Hz, 1H), 7.41 (d, *J* = 7.6 Hz, 2H), 7.32 – 7.26 (m, 3H), 7.24 (d, *J* = 2.1 Hz, 1H), 7.18 (t, *J* = 7.3 Hz, 1H), 4.79 (t, *J* = 7.1 Hz, 1H), 3.58 (s, 3H), 1.65 (d, *J* = 7.2 Hz, 2H); ^13^C NMR (126 MHz, CDCl_3_) *δ* 162.03, 154.15, 142.81, 135.62, 133.97, 131.22, 131.18, 128.42, 128.12, 126.63, 123.82, 113.55, 41.82, 29.23, 19.30 (t, *J* = 20.1 Hz); HRMS (ESI+): Calculated for C_17_H_14_DClN_2_O: [M+H]^+^ 300.1009, Found 300.1004.

**6-Bromo-1-methyl-3-(1-phenylethyl-2-d)quinoxalin-2(1H)-one (4t)**

Obtained as a white solid (43 mg, 63% yield); M.p. 176-177 ^o^C. ^1^H NMR (500 MHz, CDCl_3_) *δ* 7.76 (d, *J* = 8.5 Hz, 1H), 7.44 (dd, *J* = 8.5, 1.9 Hz, 1H), 7.41 (dd, *J* = 6.8, 1.5 Hz, 3H), 7.28 (dd, *J* = 10.3, 4.5 Hz, 2H), 7.18 (t, *J* = 7.3 Hz, 1H), 4.78 (t, *J* = 7.1 Hz, 1H), 3.58 (s, 3H), 1.64 (d, *J* = 7.1 Hz, 2H); ^13^C NMR (126 MHz, CDCl_3_) *δ* 162.28, 154.09, 142.76, 134.14, 131.56, 131.35, 128.43, 128.12, 126.70, 126.63, 123.70, 116.55, 77.28, 77.03, 76.78, 41.86, 29.22, 19.30 (t, *J* = 20.1 Hz); HRMS (ESI+): Calculated for C_17_H_14_DBrN_2_O: [M+H]^+^ 344.0504, Found 344.0504.

**1,6,7-Trimethyl-3-(1-phenylethyl-2-d)quinoxalin-2(1H)-one (4u)**

Obtained as a colourless liquid (41 mg, 70% yield); ^1^H NMR (500 MHz, CDCl_3_) δ 7.69 (s, 1H), 7.42 (d, *J* = 7.3 Hz, 2H), 7.25 (d, *J* = 6.7 Hz, 2H), 7.16 (t, *J* = 7.4 Hz, 1H), 7.02 (s, 1H), 4.80 (t, *J* = 7.1 Hz, 1H), 3.60 (s, 3H), 2.40 (s, 3H), 2.36 (s, 3H), 1.65 (d, *J* = 7.1 Hz, 2H); ^13^C NMR (126 MHz, CDCl_3_) *δ* 160.65, 154.53, 143.43, 139.42, 132.31, 131.11, 131.09, 130.22, 128.33, 128.11, 126.40, 114.09, 41.69, 29.01, 20.51, 19.60, 19.29 (t, *J* = 20.1 Hz); HRMS (ESI+): Calculated for C_19_H_19_DN_2_O: [M+H]^+^ 294.1711, Found 294.1713.

**3-(1-Phenylethyl-2-d)quinoxalin-2(1H)-one (4v)**

Obtained as a white solid (33 mg, 66% yield); M.p. 190-191 ^o^C. ^1^H NMR (500 MHz, CDCl_3_) *δ* 12.03 (s, 1H), 7.89 (d, *J* = 8.1 Hz, 1H), 7.45 (t, *J* = 7.3 Hz, 3H), 7.32 (t, *J* = 7.6 Hz, 1H), 7.27 (d, *J* = 7.5 Hz, 2H), 7.16 (dd, *J* = 18.2, 7.8 Hz, 2H), 4.82 (t, *J* = 7.0 Hz, 1H), 1.69 (d, *J* = 7.1 Hz, 2H); ^13^C NMR (126 MHz, CDCl_3_) *δ* 162.38, 156.07, 143.01, 132.82, 130.85, 129.74, 129.15, 128.34, 128.17, 126.53, 123.98, 115.54, 41.30, 19.21 (t, *J* = 20.1 Hz); HRMS (ESI+): Calculated for C_16_H_13_DN_2_O: [M+H]^+^ 252.1242, Found 252.1250

**1-Methyl-3-(1-(p-tolyl)ethyl-2-d)quinoxalin-2(1H)-one (4aa)**

Obtained as a white solid (41 mg, 73% yield); M.p. 150-151 ^o^C. ^1^H NMR (500 MHz, CDCl_3_) *δ* 7.92 (dd, *J* = 8.0, 1.3 Hz, 1H), 7.52 – 7.48 (m, 1H), 7.32 (d, *J* = 8.0 Hz, 3H), 7.23 (s, 1H), 7.08 (d, *J* = 7.9 Hz, 2H), 4.78 (t, *J* = 7.1 Hz, 1H), 3.61 (s, 3H), 2.28 (s, 3H), 1.65 (d, *J* = 7.1 Hz, 2H); ^13^C NMR (126 MHz, CDCl_3_) *δ* 162.03, 154.44, 140.11, 136.04, 133.09, 132.74, 130.11, 129.65, 129.11, 128.01, 123.42, 113.46, 41.46, 29.08, 21.06, 19.40 (t, *J* = 20.1 Hz); HRMS (ESI+): Calculated for C_18_H_17_DN_2_O: [M+H]^+^ 280.1555, Found 280.1560.

**3-(1-(4-Fluorophenyl)ethyl-2-d)-1-methylquinoxalin-2(1H)-one (4ab)**

Obtained as a colourless liquid (39 mg, 70% yield); ^1^H NMR (500 MHz, CDCl_3_) δ 7.90 (dd, *J* = 8.0, 1.4 Hz, 1H), 7.50 (ddd, *J* = 8.6, 7.4, 1.5 Hz, 1H), 7.42 – 7.37 (m, 2H), 7.35 – 7.31 (m, 1H), 7.24 (dd, *J* = 8.4, 0.8 Hz, 1H), 6.97 – 6.92 (m, 2H), 4.80 (t, *J* = 7.1 Hz, 1H), 3.62 (s, 3H), 1.64 (d, *J* = 7.2 Hz, 2H); ^13^C NMR (126 MHz, CDCl_3_) *δ* 161.65, 161.63 (d, *J* = 245.7 Hz), 154.40, 138.76 (d, *J* = 3.8 Hz), 133.07, 132.68, 130.13, 129.84, 129.59, (d, *J* = 7.6 Hz) 123.53, 115.13 (d, *J* = 21.4 Hz), 113.54, 41.06, 29.12, 19.44 (t, *J* = 20.1 Hz); ^19^F NMR (471 MHz, CDCl_3_) *δ* -116.76; HRMS (ESI+): Calculated for C_17_H_14_DFN_2_O: [M+H]^+^ 284.1304, Found 284.1311.

**3-(1-(4-Chlorophenyl)ethyl-2-d)-1-methylquinoxalin-2(1H)-one (4ac)**

Obtained as a white solid (46 mg, 78% yield); M.p. 157-158 ^o^C. ^1^H NMR (500 MHz, CDCl_3_) *δ* 7.91 (dd, *J* = 8.0, 1.3 Hz, 1H), 7.54 – 7.50 (m, 1H), 7.36 (dd, *J* = 7.7, 5.8 Hz, 3H), 7.27 – 7.22 (m, 3H), 4.78 (t, *J* = 7.1 Hz, 1H), 3.63 (s, 3H), 1.64 (d, *J* = 7.2 Hz, 2H); ^13^C NMR (126 MHz, CDCl_3_) *δ* 161.38, 154.38, 141.62, 133.07, 132.67, 132.29, 130.16, 129.92, 129.51, 128.50, 123.56, 113.56, 41.27, 29.12, 19.27 (t, *J* = 20.1 Hz); HRMS (ESI+): Calculated for C_17_H_14_DClN_2_O: [M+H]^+^ 300.1009, Found 300.1022.

**3-(1-(4-Bromophenyl)ethyl-2-d)-1-methylquinoxalin-2(1H)-one (4ad)**

Obtained as a white solid (55 mg, 80% yield); M.p. 185-186 ^o^C. ^1^H NMR (500 MHz, CDCl_3_) *δ* 7.91 (dd, *J* = 8.0, 1.1 Hz, 1H), 7.54 – 7.49 (m, 1H), 7.39 – 7.29 (m, 5H), 7.25 (d, *J* = 8.3 Hz, 1H), 4.76 (t, *J* = 7.1 Hz, 1H), 3.62 (s, 3H), 1.64 (d, *J* = 7.2 Hz, 2H); ^13^C NMR (126 MHz, CDCl_3_) *δ* 161.29, 154.37, 142.16, 133.07, 132.66, 131.45, 130.16, 129.94, 129.92, 123.58, 120.44, 113.57, 41.35, 29.13, 19.22 (t, *J* = 20.1 Hz); HRMS (ESI+): Calculated for C_17_H_14_DBrN_2_O: [M+H]^+^ 344.0504, Found 344.0501.

**1-Methyl-3-(1-(4-(trifluoromethyl)phenyl)ethyl-2-d)quinoxalin-2(1H)-one (4ae)**

Obtained as a white solid (47 mg, 70% yield); M.p. 115-116 ^o^C. ^1^H NMR (500 MHz, CDCl_3_) *δ* 7.93 (dd, *J* = 8.0, 1.3 Hz, 1H), 7.56 – 7.51 (m, 5H), 7.37 (dd, *J* = 11.2, 4.0 Hz, 1H), 7.28 (d, *J* = 8.3 Hz, 1H), 4.87 (t, *J* = 7.1 Hz, 1H), 3.64 (s, 3H), 1.68 (d, *J* = 7.1 Hz, 2H); ^13^C NMR (126 MHz, CDCl_3_) *δ* 161.00, 154.39, 147.24, 133.08, 132.66, 130.22, 130.07, 128.45, 125.32, (q, *J* = 3.8 Hz), 123.64, 113.59, 41.76, 29.15, 19.20 (t, *J* = 20.1 Hz); ^19^F NMR (471 MHz, CDCl_3_) *δ* -62.38; HRMS (ESI+): Calculated for C_18_H_14_DF_3_N_2_O: [M+H]^+^ 334.1272, Found 334.1265.

**1-Methyl-3-(1-(m-tolyl)ethyl-2-d)quinoxalin-2(1H)-one (4af)**

Obtained as a colourless liquid (40 mg, 72% yield); ^1^H NMR (500 MHz, CDCl_3_) δ 7.93 (dd, *J* = 8.0, 1.4 Hz, 1H), 7.53 – 7.49 (m, 1H), 7.36 – 7.32 (m, 1H), 7.27 – 7.22 (m, 3H), 7.16 (t, *J* = 7.8 Hz, 1H), 6.99 (d, *J* = 7.4 Hz, 1H), 4.78 (t, *J* = 7.1 Hz, 1H), 3.62 (s, 3H), 2.30 (s, 3H), 1.65 (d, *J* = 7.1 Hz, 2H); ^13^C NMR (126 MHz, CDCl_3_) δ 161.95, 154.45, 143.09, 137.89, 133.10, 132.75, 130.14, 129.66, 128.81, 128.25, 127.32, 125.11, 123.40, 113.46, 41.75, 29.09, 21.48, 19.48 (t, *J* = 20.1 Hz); HRMS (ESI+): Calculated for C_18_H_17_DN_2_O: [M+H]^+^ 280.1555, Found 280.1557.

**3-(1-(3-Fluorophenyl)ethyl-2-d)-1-methylquinoxalin-2(1H)-one (4ag)**

Obtained as a colourless liquid (42 mg, 75% yield); ^1^H NMR (500 MHz, CDCl_3_) *δ* 7.91 (dd, *J* = 8.0, 1.4 Hz, 1H), 7.53 – 7.49 (m, 1H), 7.36 – 7.33 (m, 1H), 7.25 (d, *J* = 8.3 Hz, 1H), 7.22 (dd, *J* = 5.7, 4.7 Hz, 2H), 7.14 – 7.11 (m, 1H), 6.89 – 6.83 (m, 1H), 4.82 (t, *J* = 7.1 Hz, 1H), 3.63 (s, 3H), 1.65 (d, *J* = 7.1 Hz, 2H); ^13^C NMR (126 MHz, CDCl_3_) *δ* 163.86, 162.88 (d, *J* = 245.7 Hz), 154.40, 145.77 (d, *J* = 7.6 Hz), 133.09, 132.67, 130.20, 129.94, 129.71 (d, *J* = 8.8 Hz), 123.92 (d, *J* = 2.5 Hz), 123.55, 114.87 (d, *J* = 21.4 Hz), 113.54, 113.41 (d, *J* = 20.2 Hz), 41.51, 29.13, 19.31 (t, *J* = 20.1 Hz); ^19^F NMR (471 MHz, CDCl_3_) *δ* -113.36; HRMS (ESI+): Calculated for C_17_H_14_DFN_2_O: [M+H]^+^ 284.1304, Found 284.1302.

**1-Methyl-3-(1-(3-(trifluoromethyl)phenyl)ethyl-2-d)quinoxalin-2(1H)-one (4ah)**

Obtained as a colourless liquid (43 mg, 65% yield); ^1^H NMR (500 MHz, CDCl_3_) *δ* 7.93 (dd, *J* = 8.0, 1.4 Hz, 1H), 7.69 (s, 1H), 7.64 (d, *J* = 7.7 Hz, 1H), 7.56 – 7.52 (m, 1H), 7.44 (d, *J* = 7.8 Hz, 1H), 7.41 – 7.35 (m, 2H), 7.28 (d, *J* = 8.4 Hz, 1H), 4.88 (t, *J* = 7.1 Hz, 1H), 3.64 (s, 3H), 1.68 (d, *J* = 7.2 Hz, 2H); ^13^C NMR (126 MHz, CDCl_3_) *δ* 161.05, 154.39, 144.10, 133.09, 132.66, 131.73, 130.73, 130.48, 130.25, 130.04, 128.74, 124.79 (q, *J* = 3.8 Hz), 123.62, 123.44 (q, *J* = 3.8 Hz), 113.57, 41.61, 29.17, 19.38 (t, *J* = 20.1 Hz). ^19^F NMR (471 MHz, CDCl_3_) *δ* -62.39; HRMS (ESI+): Calculated for C_18_H_14_DF_3_N_2_O: [M+H]^+^ 334.1272, Found 334.1267.

**3-(1-(2-Chlorophenyl)ethyl-2-d)-1-methylquinoxalin-2(1H)-one (4ai)**

Obtained as a colourless liquid (38 mg, 63% yield); ^1^H NMR (500 MHz, CDCl_3_) *δ* 7.90 (dd, *J* = 8.0, 1.0 Hz, 1H), 7.57 – 7.52 (m, 1H), 7.39 (dd, *J* = 5.5, 3.8 Hz, 1H), 7.35 (t, *J* = 7.5 Hz, 1H), 7.29 (d, *J* = 8.3 Hz, 1H), 7.20 (dd, *J* = 6.1, 3.4 Hz, 1H), 7.13 (dd, *J* = 5.9, 3.5 Hz, 2H), 5.24 (t, *J* = 7.0 Hz, 1H), 3.65 (s, 3H), 1.61 (d, *J* = 7.1 Hz, 2H); ^13^C NMR (126 MHz, CDCl_3_) *δ* 161.67, 154.28, 141.17, 134.09, 133.21, 132.63, 130.20, 129.90, 129.72, 128.08, 127.63, 126.72, 123.49, 113.58, 38.78, 29.13, 18.32 (t, *J* = 20.1 Hz); HRMS (ESI+): Calculated for C_17_H_14_DClN_2_O: [M+H]^+^ 300.1009, Found 300.1007.

**1-Methyl-3-(pentan-2-yl-1-d)quinoxalin-2(1H)-one (4aj)**

Obtained as a colourless liquid (35 mg, 75% yield); ^1^H NMR (500 MHz, CDCl_3_) *δ* 7.85 (dd, *J* = 8.0, 1.2 Hz, 1H), 7.53 – 7.48 (m, 1H), 7.36 – 7.27 (m, 2H), 3.70 (s, 3H), 3.55 (p, *J* = 6.9 Hz, 1H), 1.91 – 1.84 (m, 1H), 1.58 – 1.51 (m, 1H), 1.46 – 1.38 (m, 1H), 1.35 (ddd, *J* = 8.4, 7.3, 4.8 Hz, 1H), 1.29 – 1.26 (m, 2H), 0.92 (t, *J* = 7.3 Hz, 3H); ^13^C NMR (126 MHz, CDCl_3_) *δ* 164.74, 154.68, 132.89, 132.75, 129.74, 129.43, 123.39, 113.46, 36.88, 35.87, 29.08, 20.73, 17.93 (t, *J* = 20.1 Hz), 14.23; HRMS (ESI+): Calculated for C_14_H_17_DN_2_O: [M+H]^+^ 232.1555, Found 232.1554.

**1-Methyl-3-(2-methylbutan-2-yl-3-d)quinoxalin-2(1H)-one (4ak)**

Obtained as a colourless liquid (28 mg, 60% yield); ^1^H NMR (500 MHz, CDCl_3_) *δ* 7.84 (dd, *J* = 8.0, 1.3 Hz, 1H), 7.53 – 7.48 (m, 1H), 7.31 (dd, *J* = 11.2, 4.0 Hz, 1H), 7.28 (s, 1H), 3.67 (s, 3H), 2.05 – 2.00 (m, 1H), 1.43 (s, 6H), 0.75 (d, *J* = 7.6 Hz, 3H); ^13^C NMR (126 MHz, CDCl_3_) *δ* 164.80, 153.79, 133.26, 132.25, 130.16, 129.48, 123.14, 113.26, 43.02, 32.33, 31.98 (t, *J* = 20.1 Hz), 28.77, 25.84, 25.81, 9.41; HRMS (ESI+): Calculated for C_14_H_17_DN_2_O: [M+H]^+^ 232.1555, Found 232.1550.

**3-Cyclohexyl-2-d)-1-methylquinoxalin-2(1H)-one (4al)**

**3-Cyclohexyl-2-d)-1-methylquinoxalin-2(1*H*)-one (4al):** Obtained as a white solid (37 mg, 76% yield); M.p. 108-109 ^o^C. ^1^H NMR (500 MHz, CDCl_3_) *δ* 7.83 (d, *J* = 8.0 Hz, 1H), 7.51 – 7.46 (m, 1H), 7.31 (t, *J* = 7.6 Hz, 1H), 7.26 (d, *J* = 8.4 Hz, 1H), 3.68 (s, 3H), 3.33 (td, *J* = 11.4, 3.3 Hz, 1H), 2.05 – 1.88 (m, 2H), 1.86 (dd, *J* = 12.9, 2.8 Hz, 2H), 1.80 – 1.74 (m, 1H), 1.61 – 1.56 (m, 1H), 1.52 – 1.43 (m, 2H), 1.32 (tt, *J* = 12.7, 3.6 Hz, 1H); ^13^C NMR (126 MHz, CDCl_3_) *δ* 164.25, 154.52, 132.85, 129.72, 129.37, 123.36, 113.44, 40.71, 30.52, 30.47, 30.25, 30.16 (t, *J* = 20.1 Hz), 30.09, 29.94, 29.04, 26.32, 26.22, 26.14; HRMS (ESI+): Calculated for C_15_H_17_DN_2_O: [M+H]^+^ 244.1555, Found 244.1548.

**5-(4-Methyl-3-oxo-3,4-dihydroquinoxalin-2-yl)hexyl-6-d benzoate (4am)**

Obtained as a colourless liquid (57 mg, 78% yield); ^1^H NMR (500 MHz, CDCl_3_) *δ* 8.03 – 7.96 (m, 2H), 7.84 (dd, *J* = 8.0, 1.4 Hz, 1H), 7.54 – 7.49 (m, 2H), 7.39 (t, *J* = 7.8 Hz, 2H), 7.34 – 7.30 (m, 1H), 7.28 (d, *J* = 8.4 Hz, 1H), 4.31 (t, *J* = 6.6 Hz, 2H), 3.69 (s, 3H), 3.60 – 3.54 (m, 1H), 2.04 – 1.97 (m, 1H), 1.84 – 1.78 (m, 2H), 1.69 – 1.61 (m, 1H), 1.58 – 1.46 (m, 2H), 1.29 (d, *J* = 6.9 Hz, 2H); ^13^C NMR (126 MHz, CDCl_3_) *δ* 166.64, 164.24, 154.66, 132.88, 132.72, 130.48, 129.77, 129.55, 129.51, 128.26, 123.46, 113.50, 64.92, 35.98, 34.08, 29.10, 28.79, 23.98, 18.10 (t, *J* = 20.1 Hz); HRMS (ESI+): Calculated for C_22_H_23_DN_2_O_3_: [M+H]^+^ 366.1923, Found 366.1928.

**9-(4-Methyl-3-oxo-3,4-dihydroquinoxalin-2-yl)decyl-10-d benzoate (4an)**

Obtained as a yellow liquid (67 mg, 80% yield); ^1^H NMR (500 MHz, CDCl_3_) *δ* 8.03 (d, *J* = 7.7 Hz, 2H), 7.85 (d, *J* = 7.9 Hz, 1H), 7.55 – 7.47 (m, 2H), 7.42 (t, *J* = 7.7 Hz, 2H), 7.31 (t, *J* = 7.6 Hz, 1H), 7.28 – 7.25 (m, 1H), 4.29 (t, *J* = 6.7 Hz, 2H), 3.69 (s, 3H), 3.57 – 3.50 (m, 1H), 1.95 – 1.87 (m, 1H), 1.76 – 1.71 (m, 2H), 1.56 (dd, *J* = 13.4, 6.2 Hz, 1H), 1.45 – 1.38 (m, 3H), 1.32 (s, 9H); ^13^C NMR (126 MHz, CDCl_3_) *δ* 166.68, 164.68, 154.68, 132.87, 132.77, 130.52, 130.07, 129.73, 129.52, 129.46, 128.39, 128.30, 123.42, 113.50, 65.15, 36.13, 34.62, 29.67, 29.42, 29.25, 29.10, 28.71, 27.54, 26.04, 18.00 (t, *J* = 20.1 Hz); HRMS (ESI+): Calculated for C_26_H_31_DN_2_O_3_: [M+H]^+^ 422.2549, Found 422.2541.

**10-(4-Methyl-3-oxo-3,4-dihydroquinoxalin-2-yl)undecyl-11-d benzoate (4ao)**

Obtained as a colourless liquid (71 mg, 82% yield); ^1^H NMR (500 MHz, CDCl_3_) *δ* 8.04 (d, *J* = 7.3 Hz, 2H), 7.84 (dd, *J* = 7.9, 1.1 Hz, 1H), 7.53 (dd, *J* = 11.2, 3.8 Hz, 1H), 7.51 – 7.47 (m, 1H), 7.42 (t, *J* = 7.8 Hz, 2H), 7.31 (t, *J* = 7.6 Hz, 1H), 7.28 – 7.26 (m, 1H), 4.30 (t, *J* = 6.7 Hz, 2H), 3.69 (s, 3H), 3.56 – 3.49 (m, 1H), 1.93 – 1.85 (m, 1H), 1.77 – 1.71 (m, 2H), 1.58 – 1.53 (m, 1H), 1.44 – 1.28 (m, 14H); ^13^C NMR (126 MHz, CDCl_3_) *δ* 166.67, 164.73, 154.67, 132.90, 132.76, 130.54, 129.75, 129.53, 129.44, 128.31, 123.38, 113.48, 65.15, 36.12, 34.64, 29.72, 29.48, 29.46, 29.28, 29.08, 28.73, 27.56, 26.05, 18.00 (t, *J* = 20.1 Hz); HRMS (ESI+): Calculated for C_27_H_33_DN_2_O_3_: [M+H]^+^ 436.2705, Found 436.2691.

**5-(4-Methyl-3-oxo-3,4-dihydroquinoxalin-2-yl)hexyl-6-d cyclobutanecarboxylate (4ap)**

Obtained as a colourless liquid (47 mg, 69% yield); ^1^H NMR (500 MHz, CDCl_3_) *δ* 7.84 (dd, *J* = 8.0, 1.3 Hz, 1H), 7.54 – 7.50 (m, 1H), 7.35 – 7.31 (m, 1H), 7.30 (d, *J* = 8.3 Hz, 1H), 4.06 (t, *J* = 6.7 Hz, 2H), 3.71 (s, 3H), 3.56 – 3.49 (m, 1H), 3.13 – 3.05 (m, 1H), 2.27 – 2.20 (m, 2H), 2.18 – 2.12 (m, 2H), 1.96 – 1.90 (m, 2H), 1.89 – 1.84 (m, 1H), 1.66 (ddd, *J* = 8.4, 6.7, 1.8 Hz, 2H), 1.60 (ddd, *J* = 9.3, 6.5, 1.8 Hz, 1H), 1.45 – 1.37 (m, 2H), 1.27 (d, *J* = 6.9 Hz, 2H); ^13^C NMR (126 MHz, CDCl_3_) *δ* 175.58, 164.28, 154.66, 132.90, 132.79, 129.82, 129.53, 123.44, 113.49, 64.25, 38.16, 35.95, 34.11, 29.10, 28.76, 25.24, 23.86, 18.39, 18.03 (t, *J* = 20.1 Hz); HRMS (ESI+): Calculated for C_20_H_25_DN_2_O_3_: [M+H]^+^ 344.2079, Found 344.2076.

**5-(4-Methyl-3-oxo-3,4-dihydroquinoxalin-2-yl)hexyl-6-d-adamantane-1-carboxylate (4aq)**

Obtained as a colourless liquid (55 mg, 65% yield); ^1^H NMR (500 MHz, CDCl_3_) *δ* 7.85 (d, *J* = 7.9 Hz, 1H), 7.52 (dd, *J* = 11.8, 4.4 Hz, 1H), 7.33 (t, *J* = 7.6 Hz, 1H), 7.30 (d, *J* = 8.4 Hz, 1H), 4.03 (t, *J* = 6.5 Hz, 2H), 3.71 (d, *J* = 6.0 Hz, 3H), 3.57 – 3.51 (m, 1H), 1.96 (s, 4H), 1.83 (d, *J* = 2.3 Hz, 6H), 1.67 (dd, *J* = 25.2, 12.1 Hz, 9H), 1.40 (ddd, *J* = 11.8, 10.3, 6.5 Hz, 2H), 1.27 (d, *J* = 6.9 Hz, 2H); ^13^C NMR (126 MHz, CDCl_3_) *δ* 177.77, 164.32, 154.67, 132.89, 132.70, 129.77, 129.57, 123.47, 113.49, 63.98, 40.67, 38.82, 38.66, 36.51, 36.43, 35.96, 34.07, 29.12, 28.75, 27.95, 27.84, 23.86, 18.09 (t, *J* = 20.1 Hz); HRMS (ESI+): Calculated for C_26_H_33_DN_2_O_3_: [M+H]^+^ 424.2705, Found 424.2694.

**3-(6-Bromohexan-2-yl-1-d)-1-methylquinoxalin-2(1H)-one (4ar)**

Obtained as a colourless liquid (51 mg, 79% yield); ^1^H NMR (500 MHz, CDCl_3_) *δ* 7.84 (dd, *J* = 8.0, 1.3 Hz, 1H), 7.55 – 7.49 (m, 1H), 7.32 (ddd, *J* = 23.1, 12.1, 4.8 Hz, 2H), 3.70 (s, 3H), 3.52 (dd, *J* = 13.7, 6.9 Hz, 1H), 3.40 (t, *J* = 6.9 Hz, 2H), 1.96 – 1.86 (m, 3H), 1.61 – 1.45 (m, 3H), 1.28 (d, *J* = 6.9 Hz, 2H); ^13^C NMR (126 MHz, CDCl_3_) *δ* 164.12, 154.63, 132.90, 132.74, 129.80, 129.58, 123.46, 113.52, 35.88, 33.83, 33.51, 32.84, 29.12, 26.09, 18.03 (t, *J* = 20.1 Hz); HRMS (ESI+): Calculated for C_15_H_18_DBrN_2_O: [M+H]^+^ 324.0817, Found 324.0815.

**3-(5-Hydroxypentan-2-yl-1-d)-1-methylquinoxalin-2(1H)-one (4as)**

Obtained as a colourless liquid (36 mg, 73% yield); ^1^H NMR (500 MHz, CDCl_3_) *δ* 7.86 (d, *J* = 7.7 Hz, 1H), 7.53 (dd, *J* = 11.4, 4.1 Hz, 1H), 7.34 (t, *J* = 7.6 Hz, 1H), 7.31 (d, *J* = 8.4 Hz, 1H), 3.71 (s, 3H), 3.70 – 3.66 (m, 2H), 3.58 – 3.52 (m, 1H), 2.28 (s, 1H), 2.03 – 1.97 (m, 1H), 1.70 (dd, *J* = 14.3, 5.9 Hz, 1H), 1.65 – 1.59 (m, 2H), 1.30 (d, *J* = 6.8 Hz, 2H); ^13^C NMR (126 MHz, CDCl_3_) *δ* 164.08, 154.76, 132.86, 132.61, 129.72, 129.69, 123.62, 113.58, 62.50, 35.49, 30.45, 30.35, 29.19, 17.84 (t, *J* = 20.1 Hz); HRMS (ESI+): Calculated for C_14_H_17_DN_2_O_2_: [M+H]^+^ 248.1504, Found 248.1488.

**3-(6-Hydroxyhexan-2-yl-1-d)-1-methylquinoxalin-2(1H)-one (4at)**

Obtained as a colourless liquid (37 mg, 70% yield); ^1^H NMR (500 MHz, CDCl_3_) *δ* 7.85 (dd, *J* = 8.0, 1.1 Hz, 1H), 7.56 – 7.50 (m, 1H), 7.36 – 7.28 (m, 2H), 3.71 (s, 3H), 3.69 – 3.61 (m, 2H), 3.59 – 3.50 (m, 1H), 2.01 – 1.92 (m, 1H), 1.74 (s, 1H), 1.61 (ddd, *J* = 6.3, 5.3, 2.2 Hz, 3H), 1.47 (ddd, *J* = 10.7, 9.8, 5.3 Hz, 1H), 1.42 – 1.36 (m, 1H), 1.28 (d, *J* = 6.9 Hz, 2H); ^13^C NMR (126 MHz, CDCl_3_) *δ* 164.43, 154.74, 132.90, 132.72, 129.74, 129.59, 123.52, 113.53, 62.80, 36.07, 34.06, 32.58, 29.14, 23.65, 18.17 (t, *J* = 20.1 Hz); HRMS (ESI+): Calculated for C_15_H_19_DN_2_O_2_: [M+H]^+^ 262.1661, Found 262.1670.

**3-(10-Hydroxydecan-2-yl-1-d)-1-methylquinoxalin-2(1H)-one (4au)**

Obtained as a colourless liquid (44 mg, 69% yield); ^1^H NMR (500 MHz, CDCl_3_) *δ* 7.86 (dd, *J* = 7.9, 0.9 Hz, 1H), 7.54 – 7.49 (m, 1H), 7.35 – 7.31 (m, 1H), 7.30 (d, *J* = 8.4 Hz, 1H), 3.71 (s, 3H), 3.62 (t, *J* = 6.7 Hz, 2H), 3.56 – 3.49 (m, 1H), 1.92 – 1.85 (m, 1H), 1.71 (s, 1H), 1.58 – 1.52 (m, 3H), 1.35 – 1.26 (m, 12H); ^13^C NMR (126 MHz, CDCl_3_) *δ* 164.76, 154.69, 132.90, 132.75, 129.74, 129.48, 123.43, 113.49, 63.04, 36.09, 34.61, 32.77, 29.60, 29.43, 29.32, 29.12, 27.51, 25.68, 17.97 (t, *J* = 20.1 Hz); HRMS (ESI+): Calculated for C_19_H_27_DN_2_O_2_: [M+H]^+^ 318.2287, Found 318.2287.

**3-(11-Hydroxyundecan-2-yl-1-d)-1-methylquinoxalin-2(1H)-one (4av)**

Obtained as a colourless liquid (43 mg, 65% yield); ^1^H NMR (500 MHz, CDCl_3_) *δ* 7.89 (d, *J* = 6.9 Hz, 1H), 7.53 (t, *J* = 7.2 Hz, 1H), 7.34 (t, *J* = 5.9 Hz, 1H), 7.30 (d, *J* = 8.1 Hz, 1H), 3.71 (s, 3H), 3.63 (t, *J* = 6.6 Hz, 2H), 3.54 (s, 1H), 2.00 (s, 1H), 1.90 (s, 1H), 1.63 – 1.49 (m, 3H), 1.28 (d, *J* = 6.3 Hz, 14H); ^13^C NMR (126 MHz, CDCl_3_) *δ* 164.83, 154.68, 132.95, 132.50, 129.65, 129.62, 123.53, 113.55, 63.08, 36.18, 34.64, 32.80, 29.68, 29.50, 29.41, 29.36, 29.25, 27.57, 25.70, 18.02 (t, *J* = 20.1 Hz); HRMS (ESI+): Calculated for C_20_H_29_DN_2_O_2_: [M+H]^+^ 332.2443, Found 332.2424.

**1-Methyl-3-(-6-((13-methyl-17-oxo-7,8,9,11,12,13,14,15,16,17-decahydro-6H-cyclopenta[a]phenanthren-3-yl)oxy)hexan-2-yl-1-d)quinoxalin-2(1H)-one (4bb)**

Obtained as a colourless liquid (67 mg, 65% yield); ^1^H NMR (500 MHz, CDCl_3_) *δ* 7.84 (d, *J* = 7.7 Hz, 1H), 7.54 – 7.49 (m, 1H), 7.33 (dd, *J* = 11.2, 4.1 Hz, 1H), 7.29 (d, *J* = 8.3 Hz, 1H), 7.16 (d, *J* = 8.6 Hz, 1H), 6.68 (dd, *J* = 8.6, 2.5 Hz, 1H), 6.61 (d, *J* = 2.4 Hz, 1H), 3.92 (t, *J* = 6.6 Hz, 2H), 3.70 (s, 3H), 3.55 (t, *J* = 6.9 Hz, 1H), 2.90 – 2.84 (m, 2H), 2.50 (dd, *J* = 19.0, 8.6 Hz, 1H), 2.38 (dd, *J* = 9.4, 4.0 Hz, 1H), 2.23 (dd, *J* = 13.6, 7.3 Hz, 1H), 2.14 (dd, *J* = 18.6, 9.4 Hz, 1H), 2.08 – 2.03 (m, 1H), 2.01 – 1.93 (m, 3H), 1.85 – 1.74 (m, 3H), 1.66 – 1.60 (m, 2H), 1.57 – 1.47 (m, 5H), 1.43 (dd, *J* = 12.0, 4.0 Hz, 1H), 1.28 (d, *J* = 6.8 Hz, 2H), 0.90 (s, 3H); ^13^C NMR (126 MHz, CDCl_3_) *δ* 164.41, 157.14, 154.68, 137.64, 132.92, 132.81, 131.78, 129.82, 129.53, 126.25, 123.44, 114.59, 113.51, 112.17, 67.78, 50.43, 48.05, 43.99, 38.40, 36.08, 35.90, 34.28, 31.60, 29.65, 29.43, 29.12, 26.59, 25.93, 24.05, 21.60, 18.02 (t, *J* = 20.1 Hz), 13.87; HRMS (ESI+): Calculated for C_33_H_39_DN_2_O_3_: [M+H]^+^ 514.3175, Found 514.3174.

**Diethyl 3-(methyl-d)-4-((4-methyl-3-oxo-3,4-dihydroquinoxalin-2-yl)methyl)cyclopentane-1,1-dicarboxylate (6a)**

Obtained as a colourless liquid (56 mg, 70% yield); ^1^H NMR (500 MHz, CDCl_3_) *δ* 7.82 (dd, *J* = 7.9, 1.2 Hz, 1H), 7.54 – 7.50 (m, 1H), 7.35 – 7.29 (m, 2H), 4.21 – 4.14 (m, 4H), 3.70 (s, 3H), 3.07 (dd, *J* = 15.2, 5.8 Hz, 1H), 2.82 (dd, *J* = 15.2, 8.8 Hz, 1H), 2.78 – 2.71 (m, 1H), 2.47 (ddd, *J* = 13.7, 9.8, 7.1 Hz, 2H), 2.36 (d, *J* = 5.9 Hz, 1H), 2.19 – 2.06 (m, 2H), 1.23 (td, *J* = 7.1, 4.7 Hz, 6H), 0.94 (d, *J* = 7.1 Hz, 2H). ^13^C NMR (126 MHz, CDCl_3_) *δ* 172.94, 172.80, 160.04, 154.88, 133.04, 132.71, 129.78, 129.59, 123.48, 113.54, 61.32, 61.31, 59.01, 41.35, 39.77, 38.29, 35.88, 34.22, 29.05, 14.95 (t, *J* = 18.9 Hz), 14.04.HRMS (ESI+): Calculated for C_22_H_27_DN_2_O_5_: [M+H]^+^ 402.2134, Found 402.2139.

1. **X-ray Crystal Data for 4al**


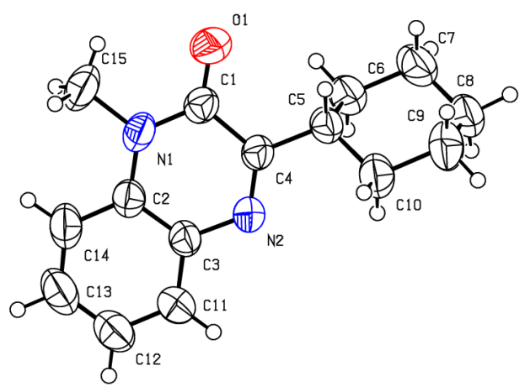


**Figure S1** Single-crystal X-ray structure of **4al**. Ellipsoids are represented at 30% probability.

**Table S1** Crystallographic data and structure refinement for **4al**

| CCDC | 1975822 |
| --- | --- |
| Empirical formula | C_15_H_18_N_2_O |
| Formula weight | 242.31 |
| Temperature, K | 296.15 |
| Wavelength, Å | 0.71073 |
| Crystal system | Triclinic |
| Space group | P-1 |
| *a*, *b*, *c*, Å | 10.391(2), 12.069(3), 12.450(3) |
| *α*, *β*, *γ*, ^o^ | 84.497(4), 69.931(4), 64.785(4) |
| Volume, Å^3^ | 1324.5(5) |
| *Z* | 4 |
| Calculated density, Mg/m^3 | 1.215 |
| *F* (000) | 520 |
| Theta range for data collection, ^o^ | 2.273 to 27.512 |
| Limiting indices | -13<=h<=13, -15<=k<=14, -13<=l<=16 |
| Reflections collected / unique | 7968 / 5724 [R(int) = 0.0253] |
| Absorption correction | Semi-empirical from equivalents |
| Refinement method | Full-matrix least-squares on F^2^ |
| Data / restraints / parameters | 5724 / 0 / 328 |
| Goodness of fit on *F*^2^ | 1.043 |

1. **Copies of ^1^H, ^13^C and ^19^F NMR Spectra**

**4a ^1^H NMR**


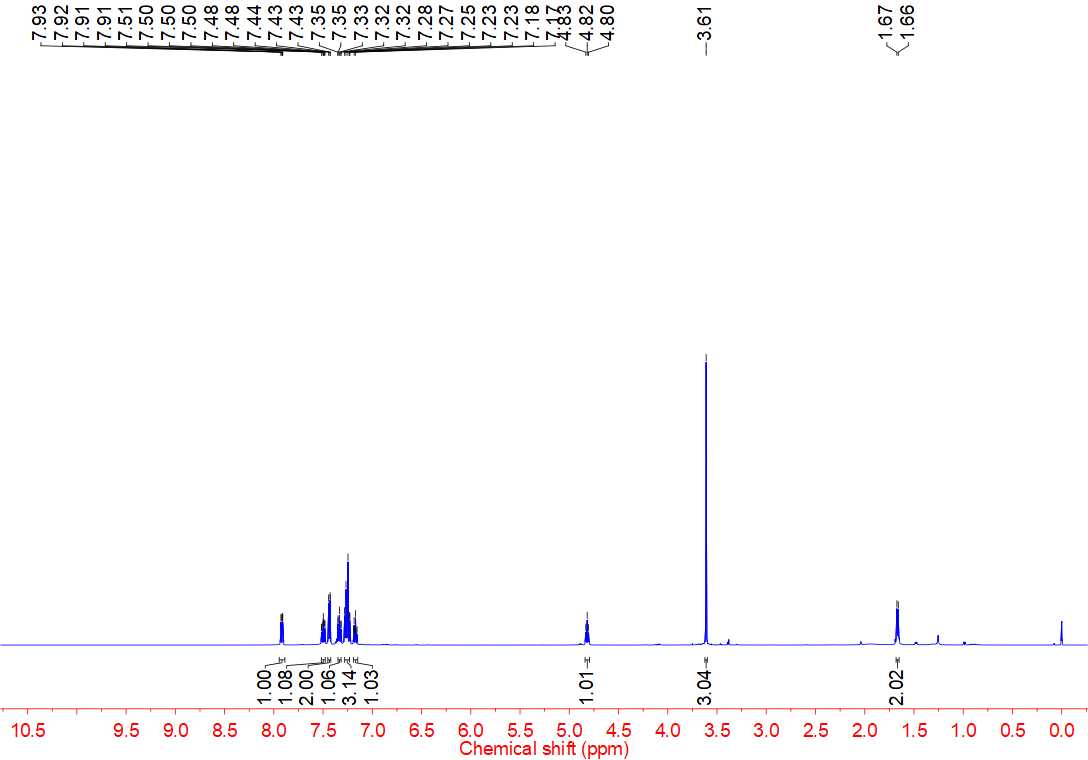

**4a ^13^C NMR**


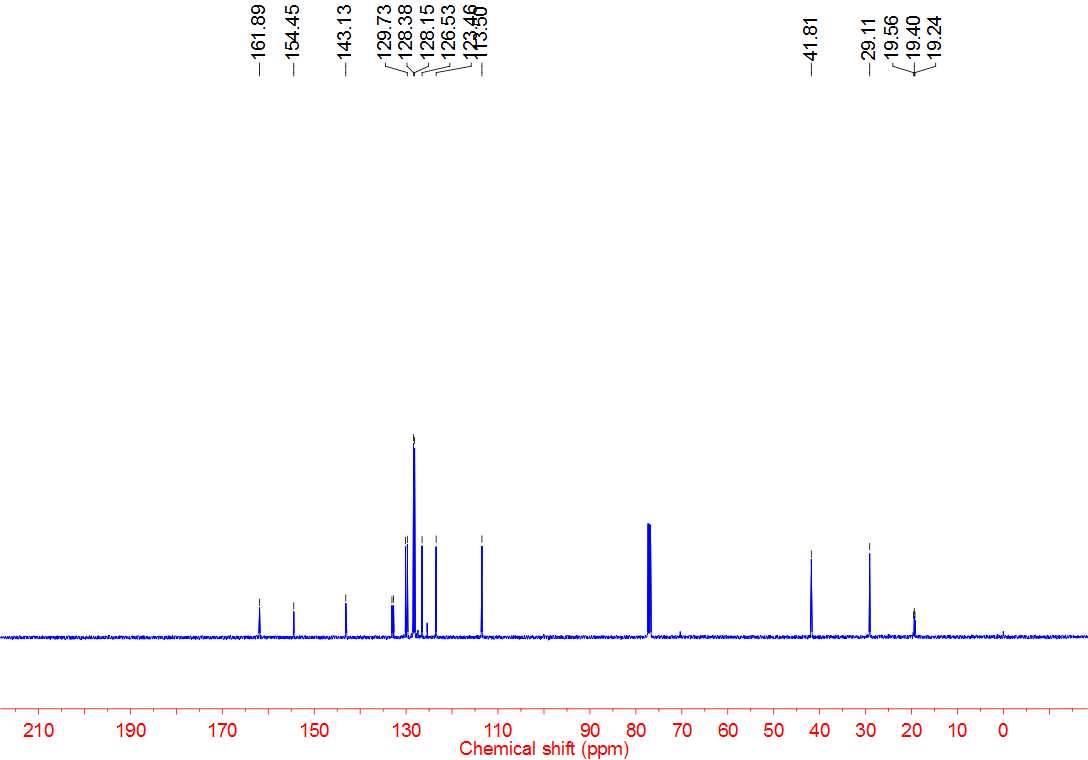

**4b ^1^H NMR**


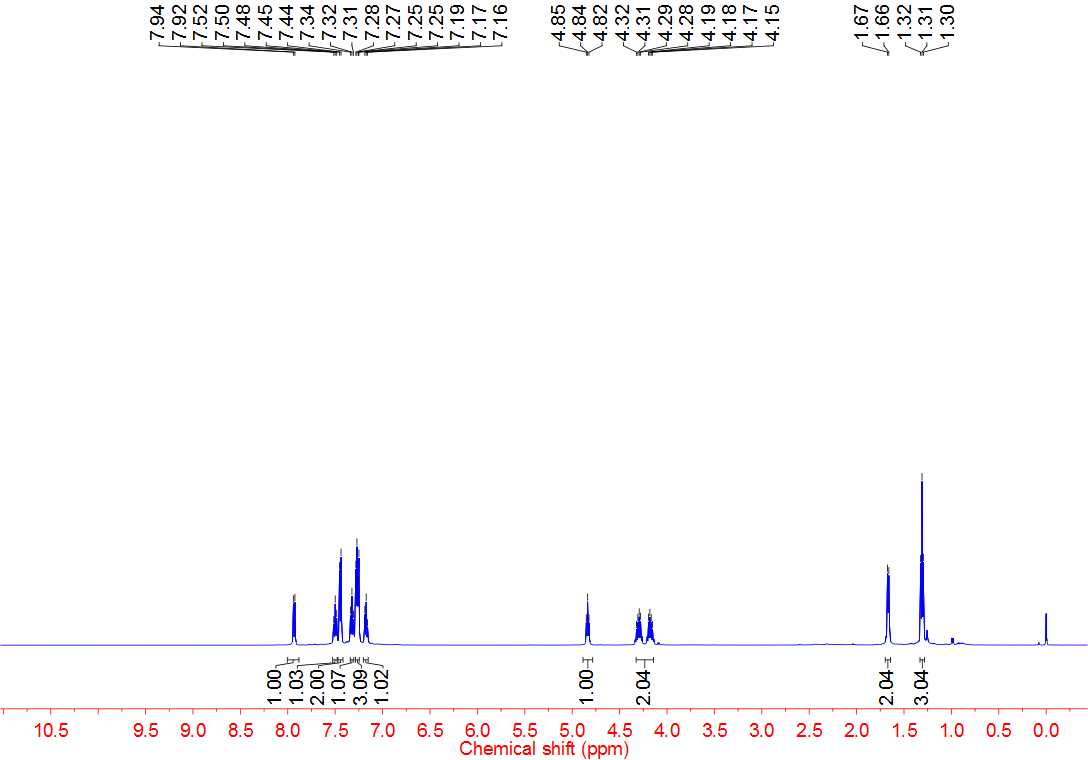

**4b ^13^C NMR**


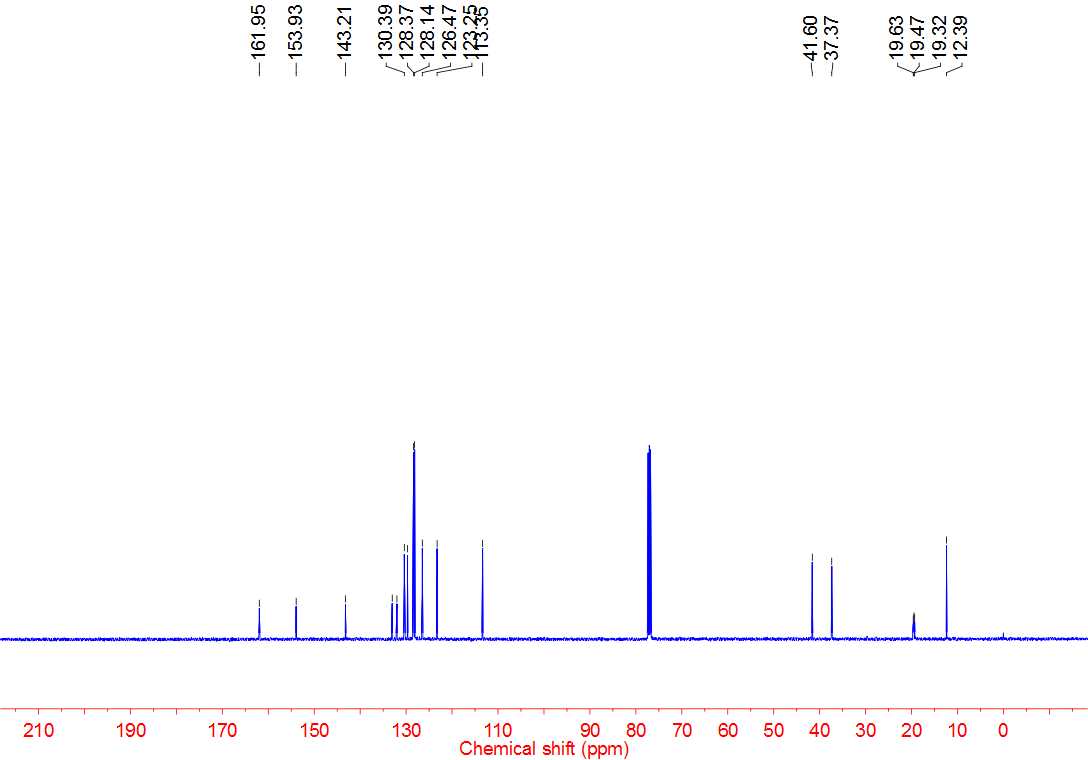

**4c ^1^H NMR**


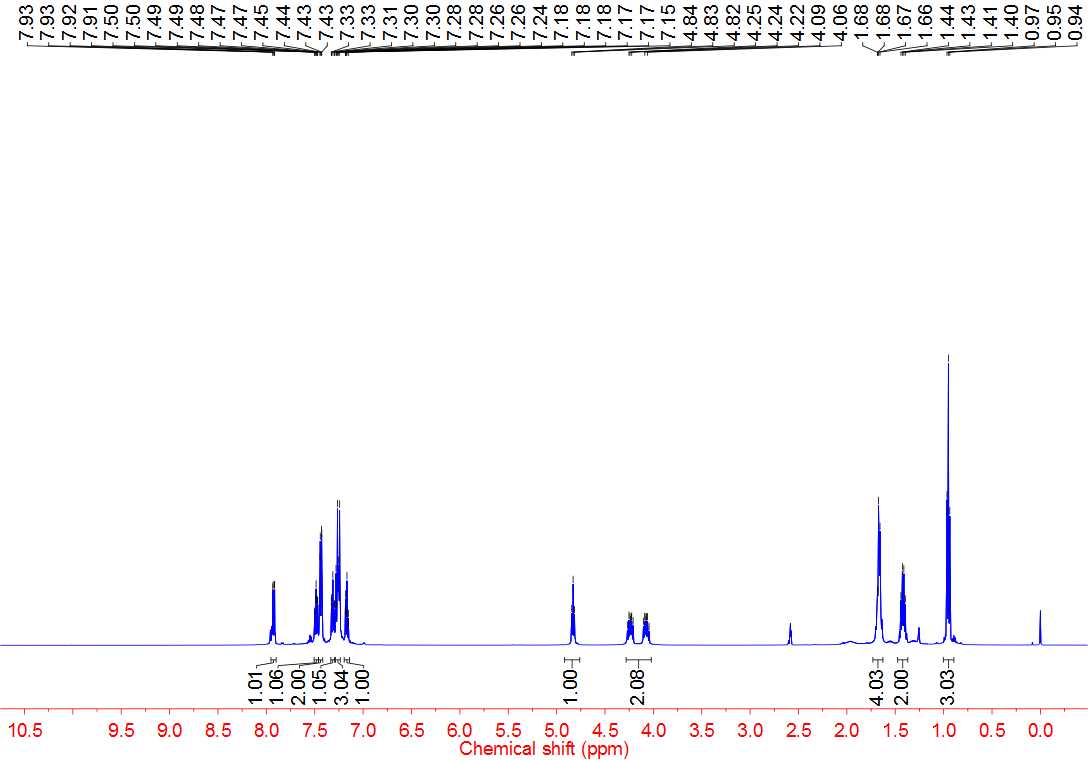

**4c ^13^C NMR**


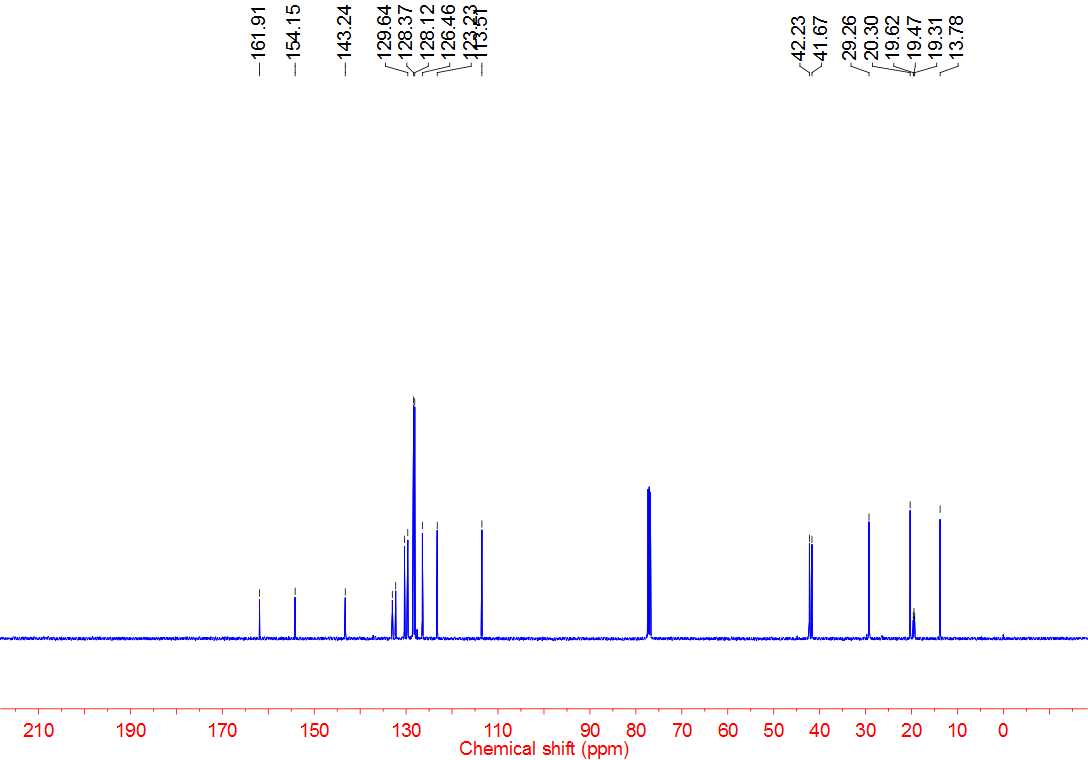

**4d ^1^H NMR**


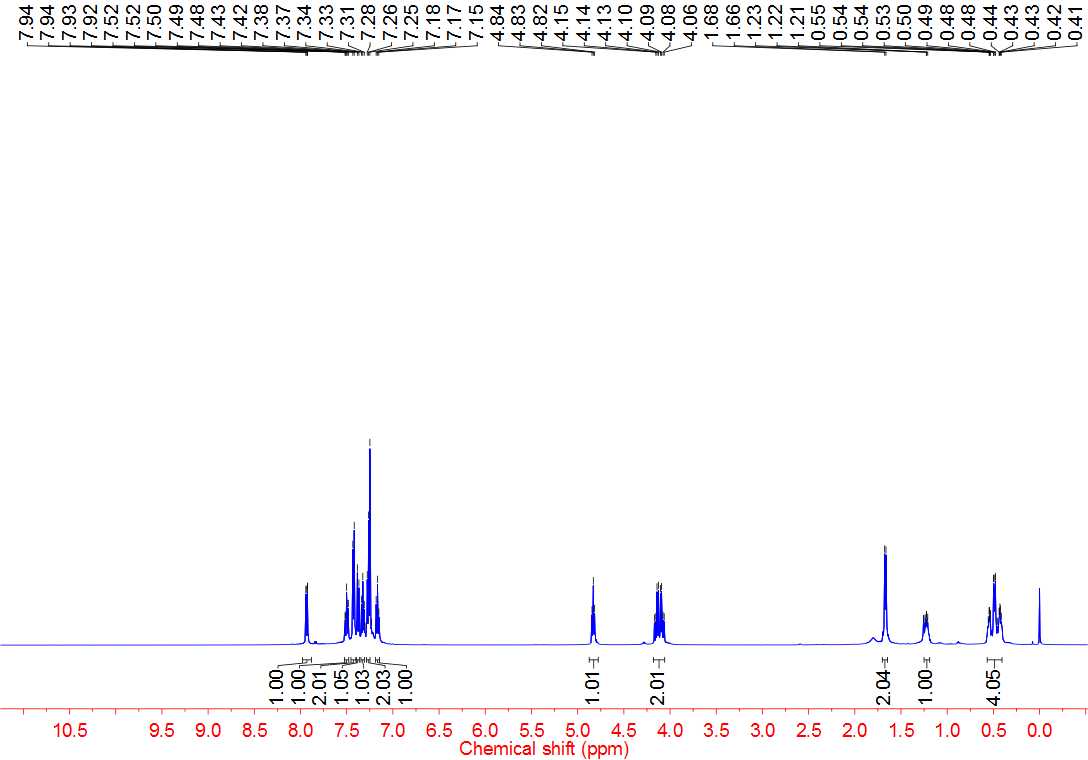

**4d ^13^C NMR**


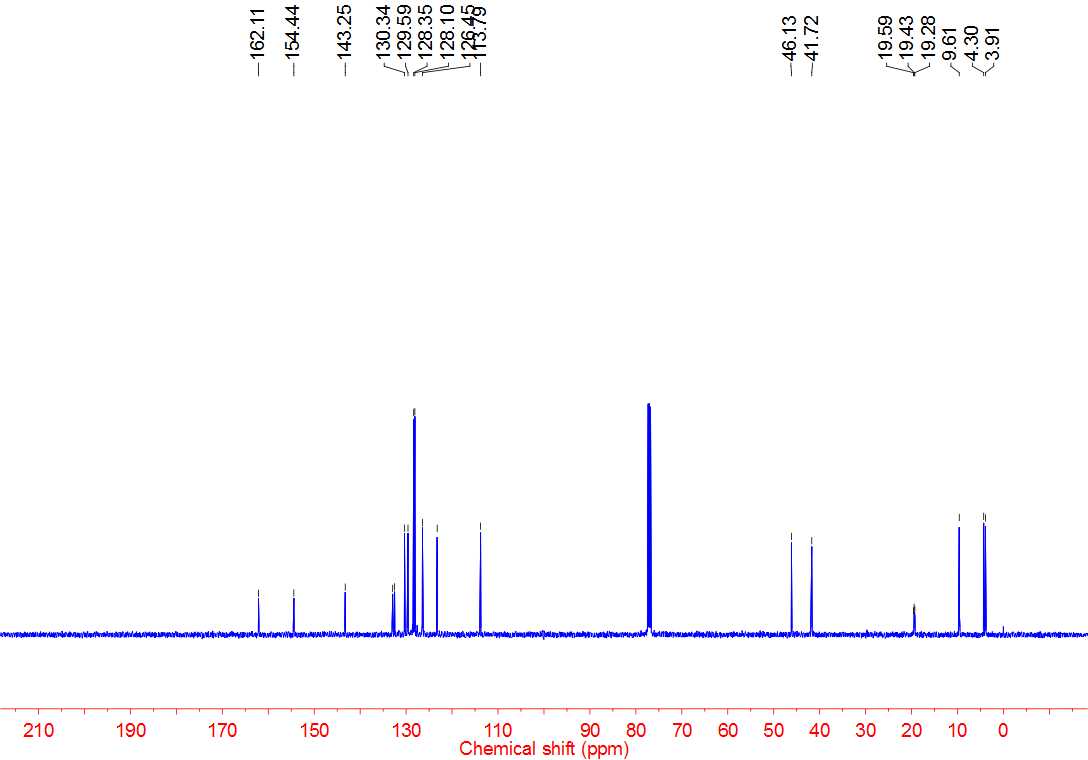

**4e ^1^H NMR**


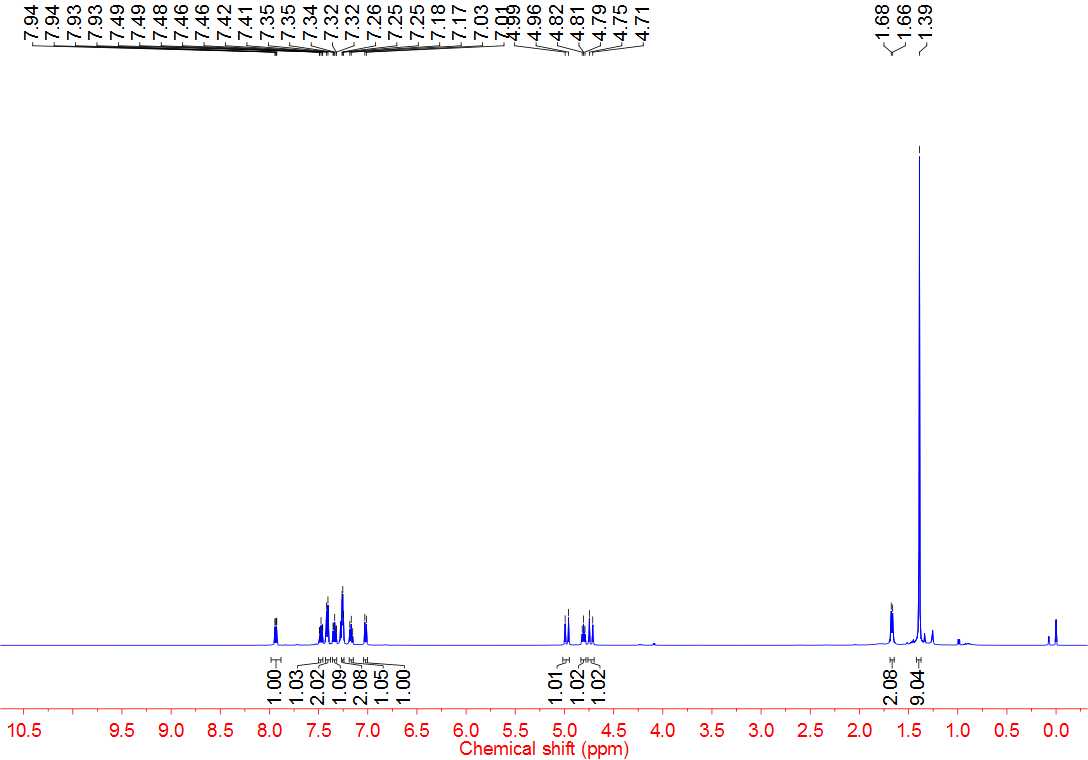

**4e ^13^C NMR**


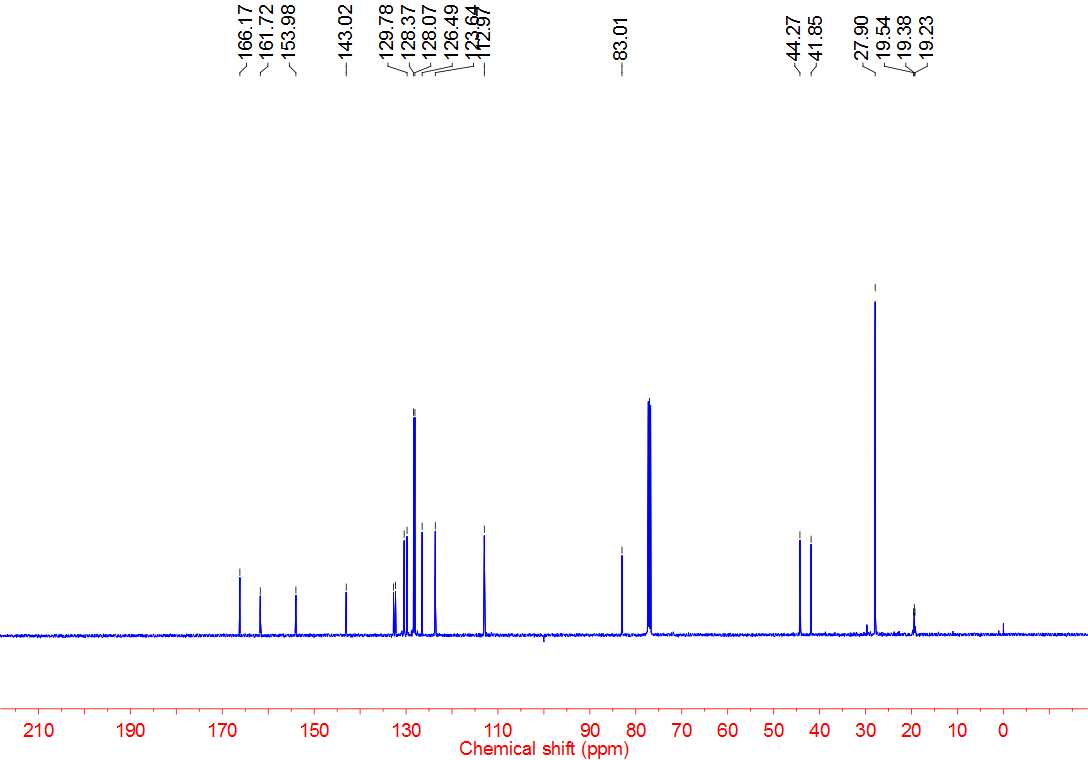

**4f ^1^H NMR**


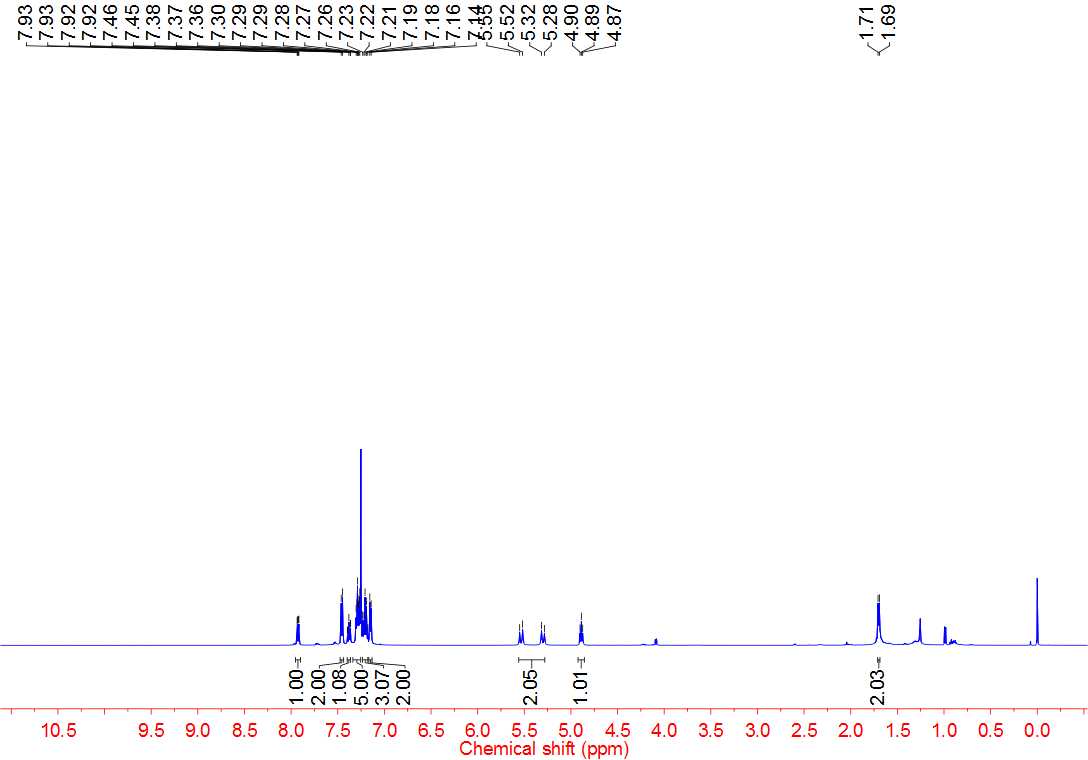

**4f ^13^C NMR**


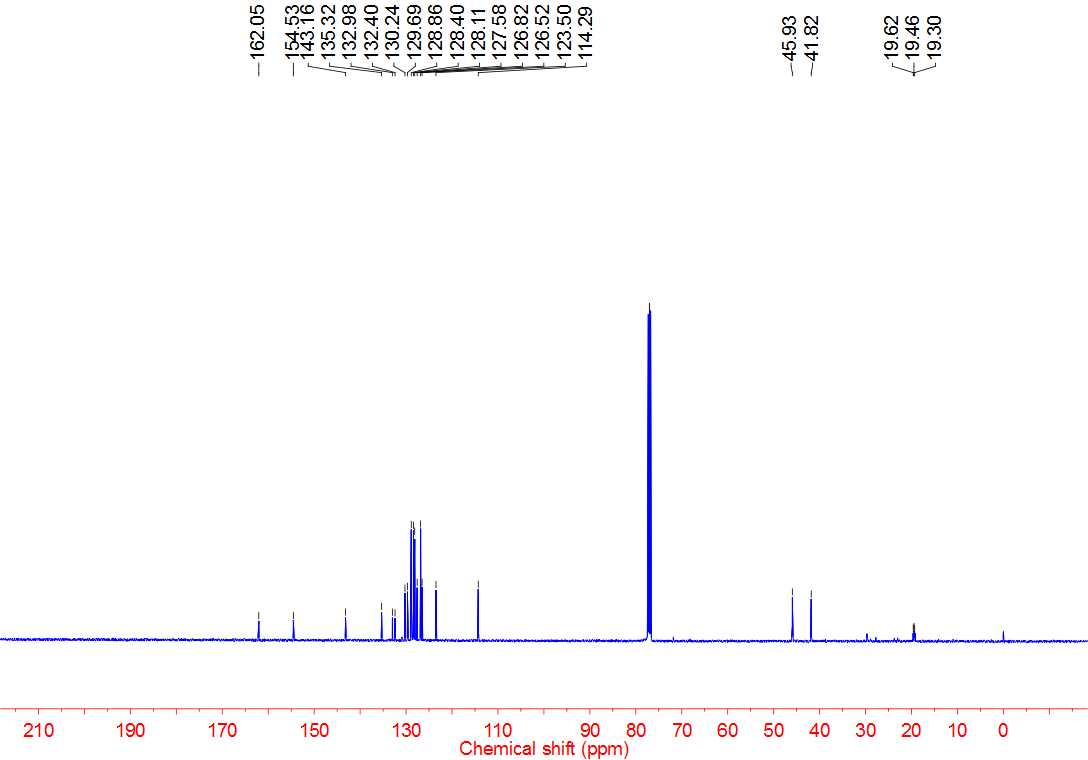

**4g ^1^H NMR**


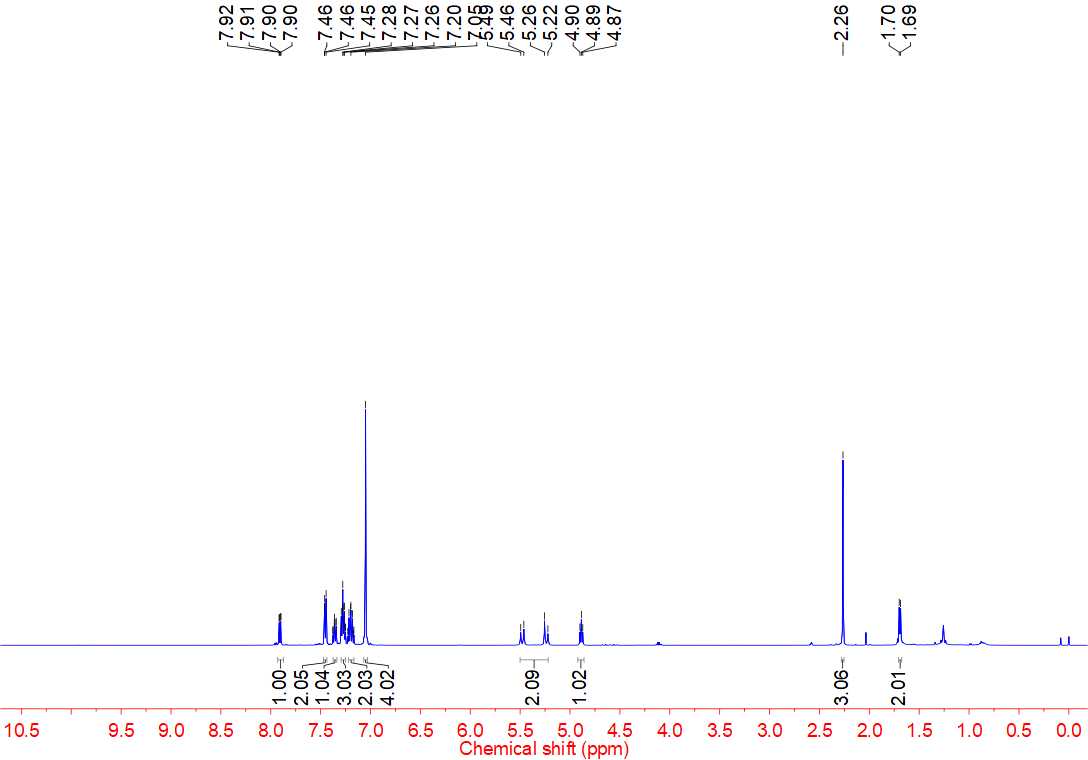

**4g ^13^C NMR**


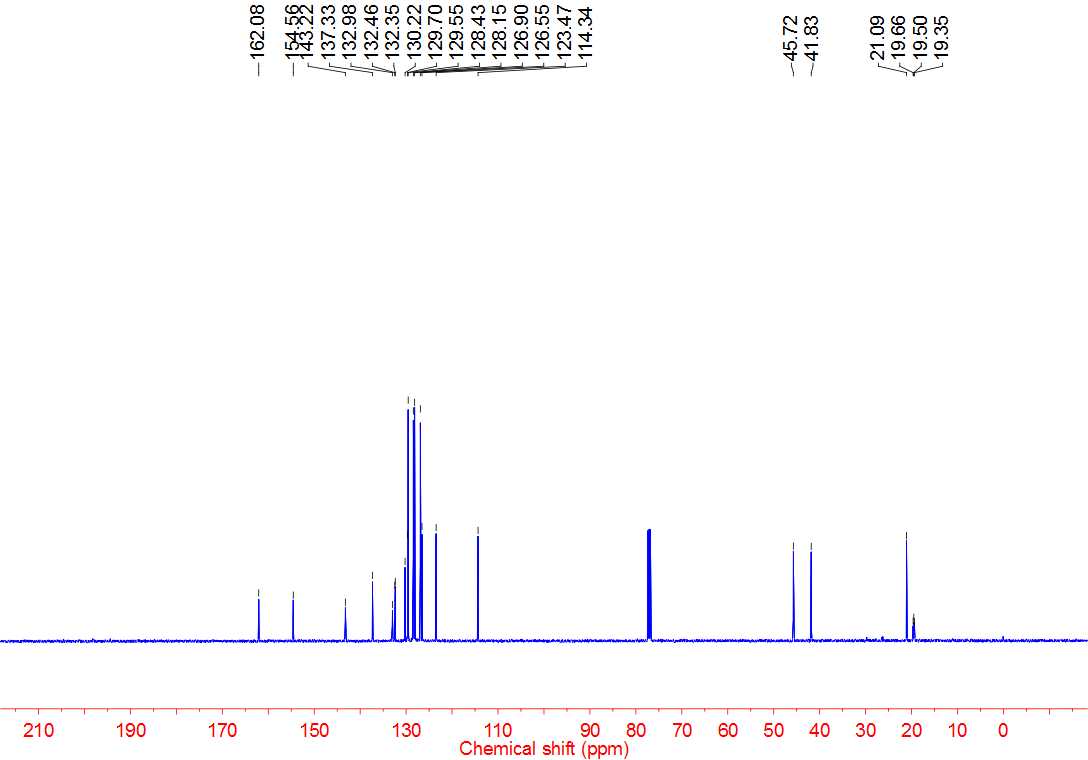

**4h ^1^H NMR**


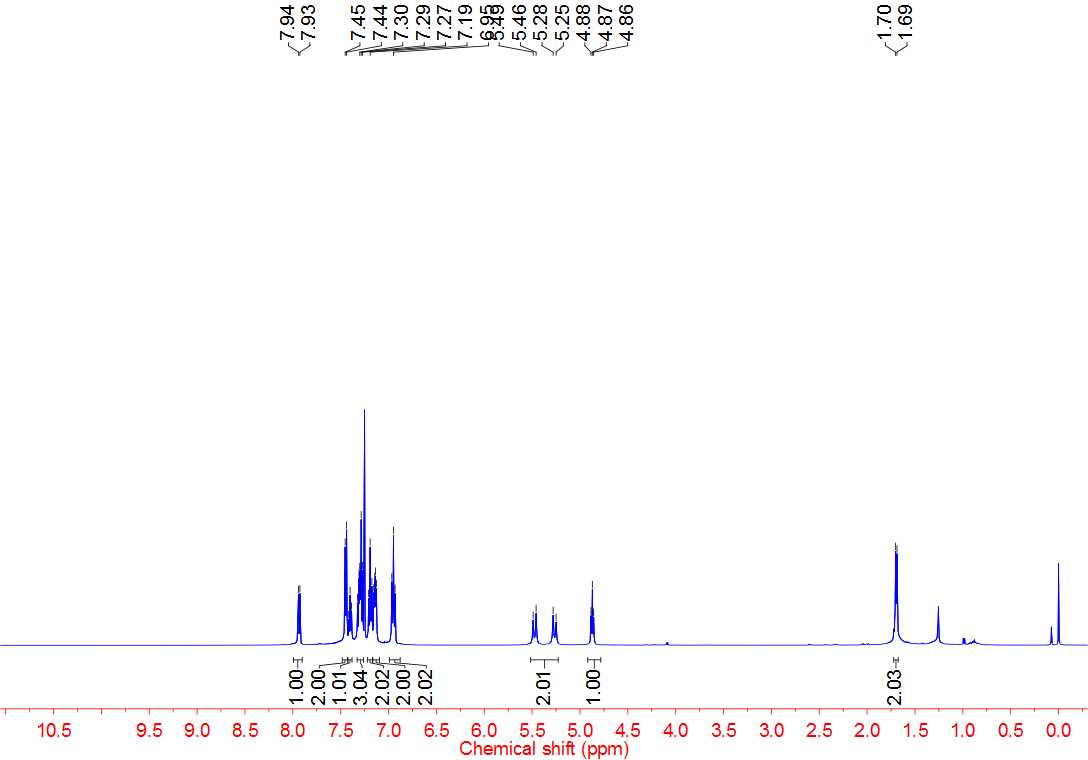

**4h ^13^C NMR**

**
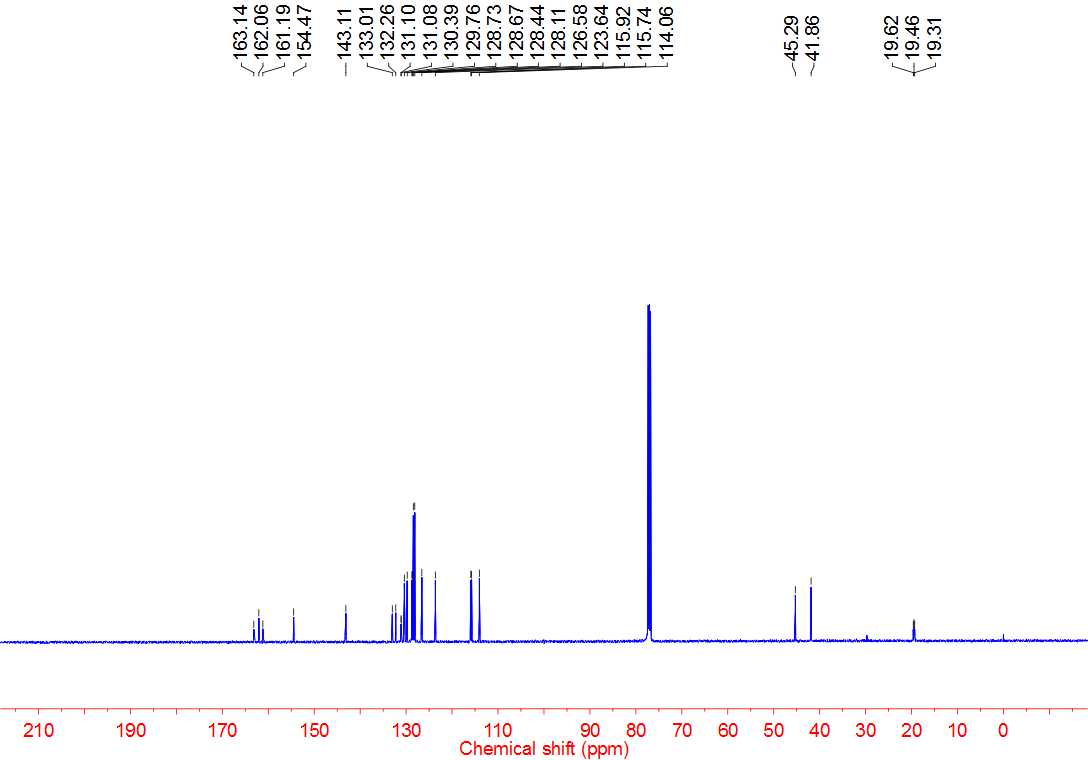
**

**4h ^19^F NMR**

**
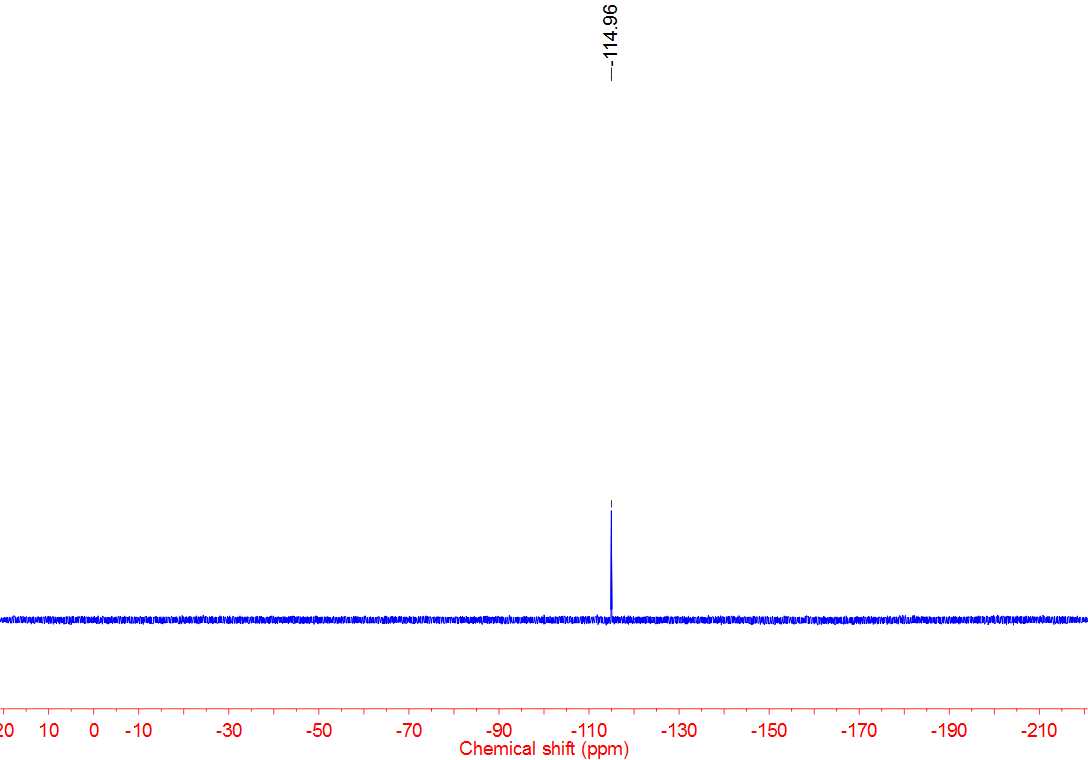
**

**4i ^1^H NMR**

**
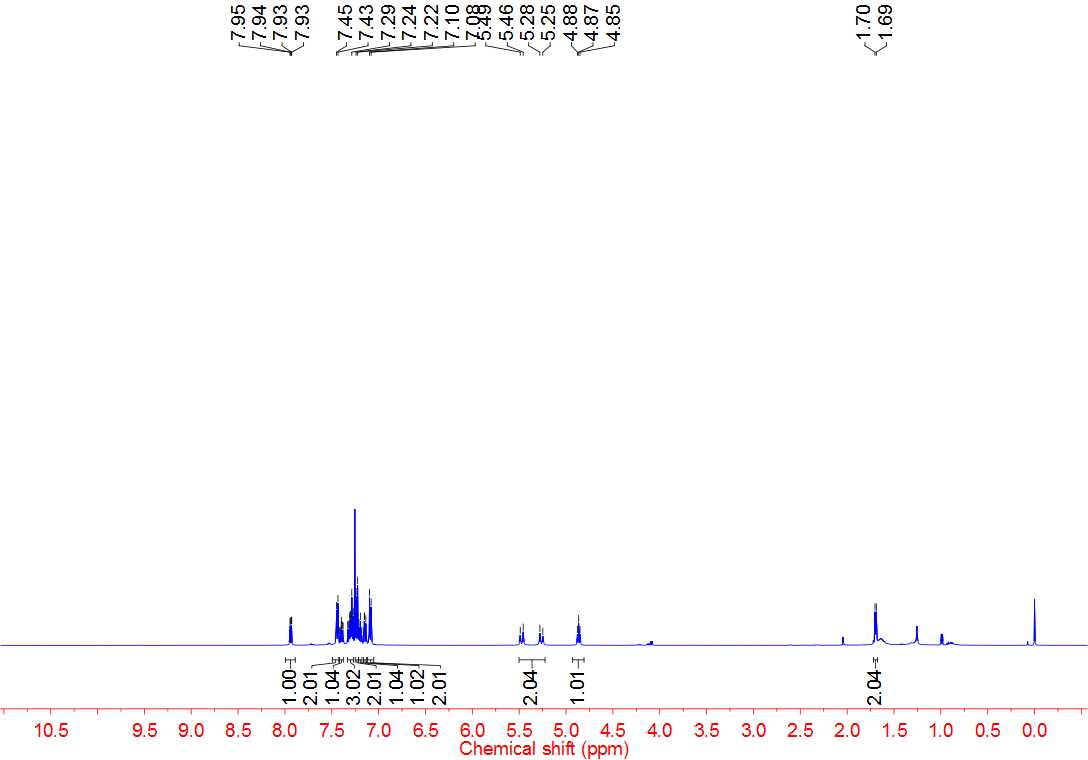
**

**4i ^13^C NMR**

**
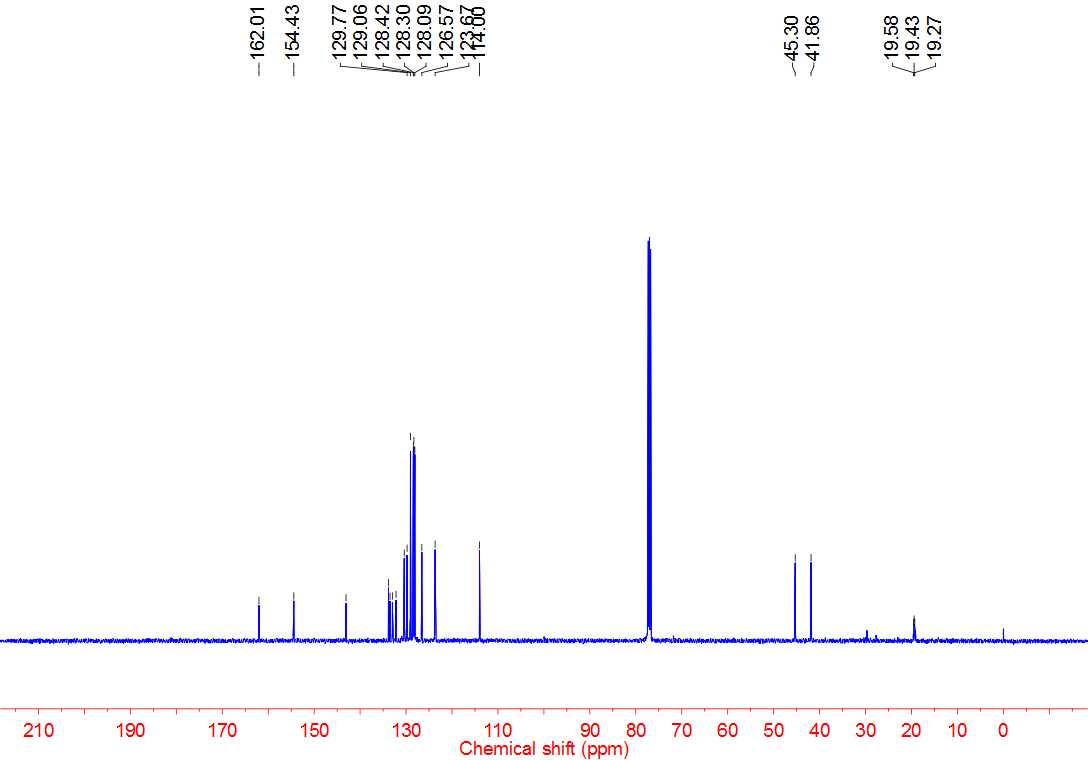
**

**4j ^1^H NMR**

**
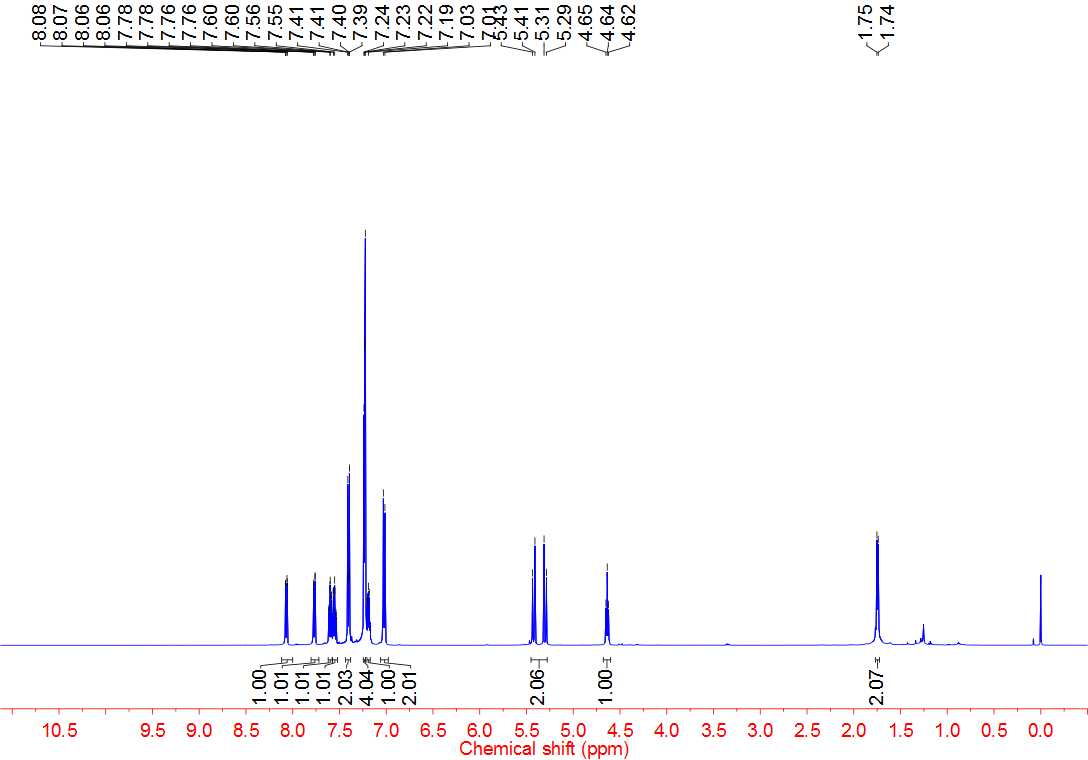
**

**4j ^13^C NMR**

**
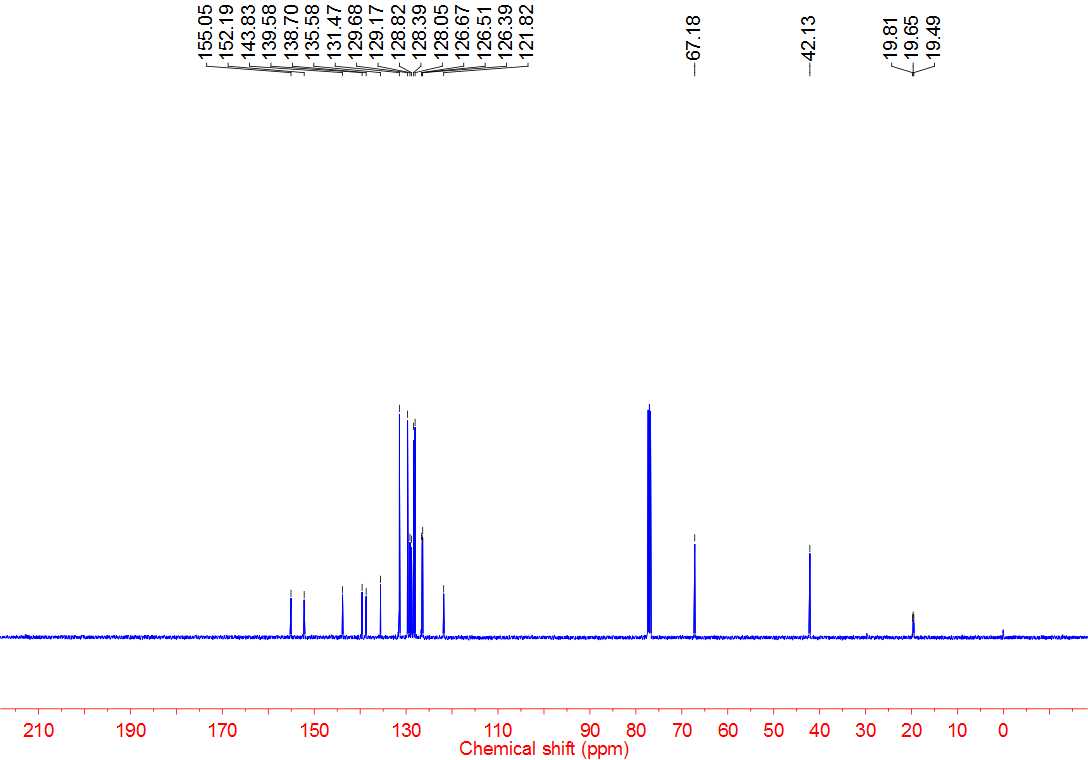
**

**4k ^1^H NMR**

**
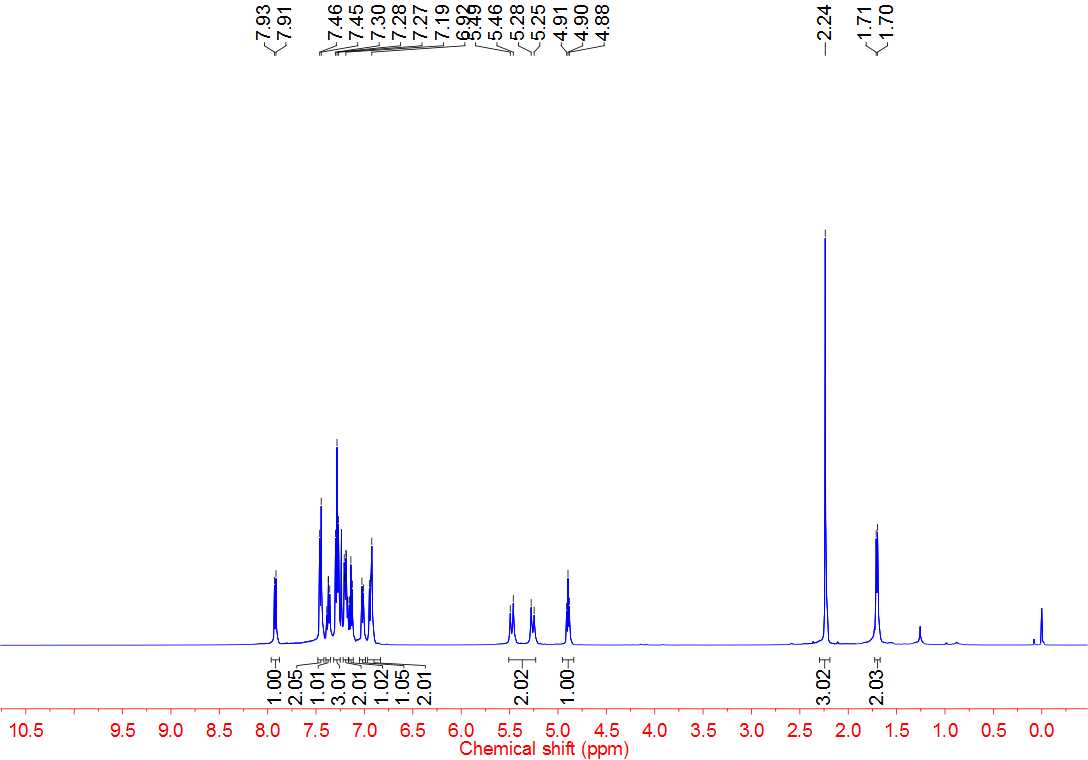
**

**4k ^13^C NMR**

**
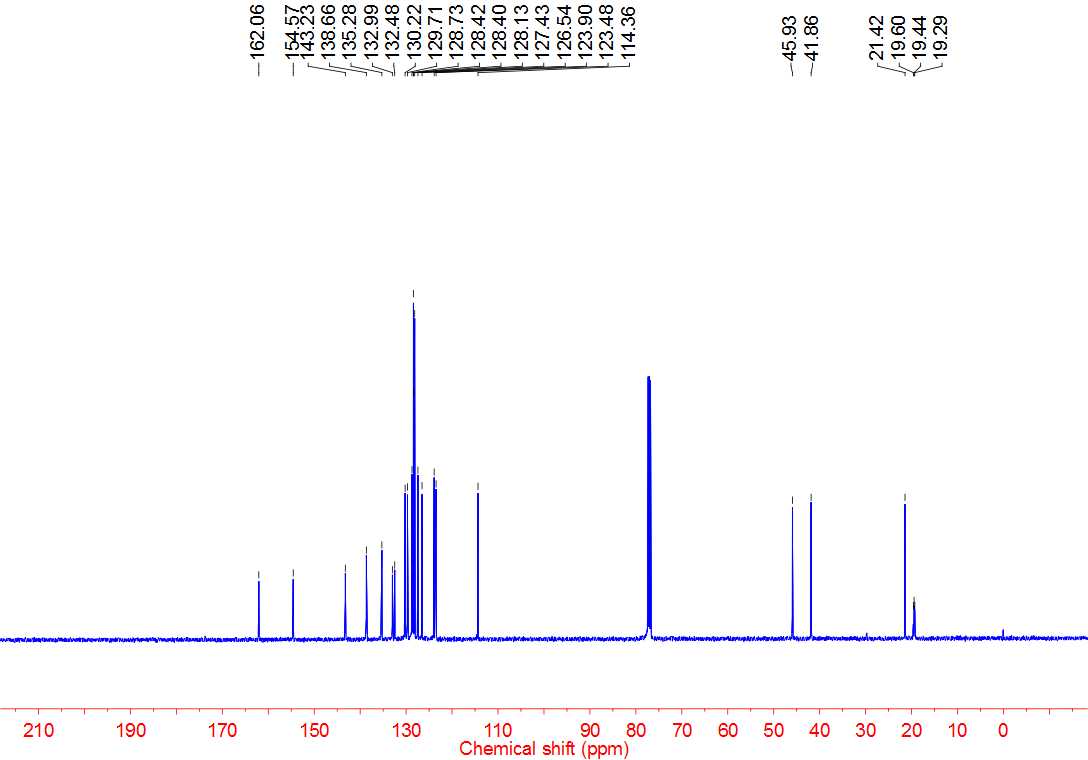
**

**4l ^1^H NMR**

**
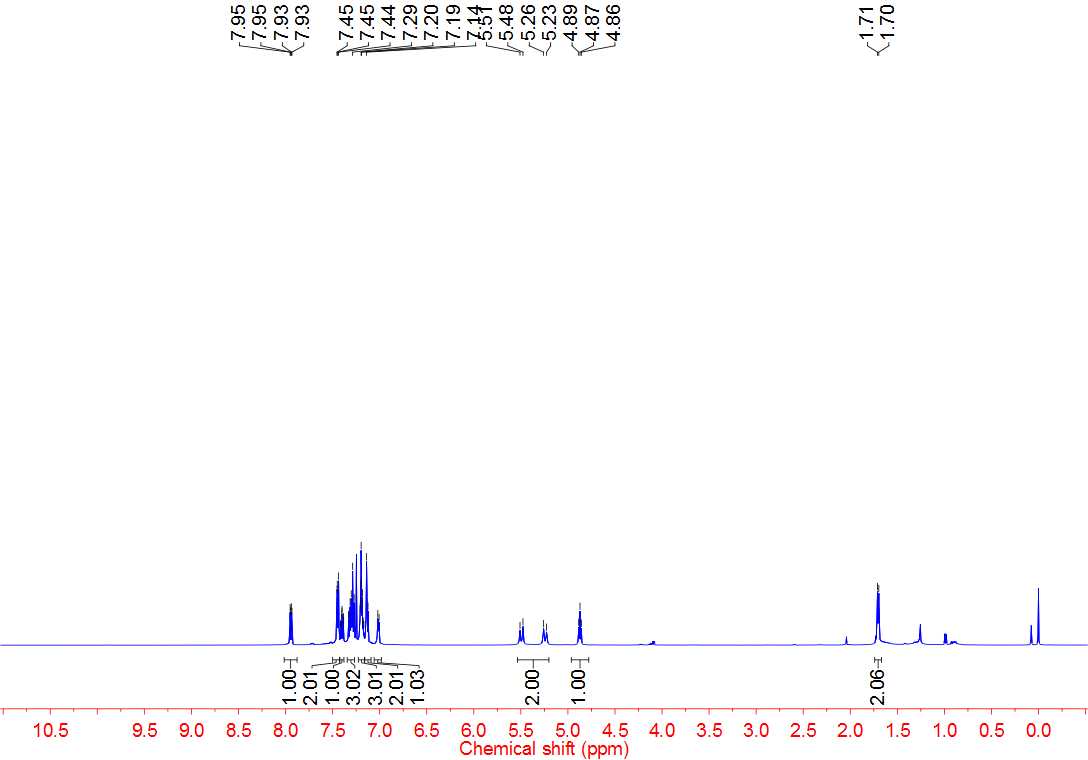
**

**4l ^13^C NMR**

**
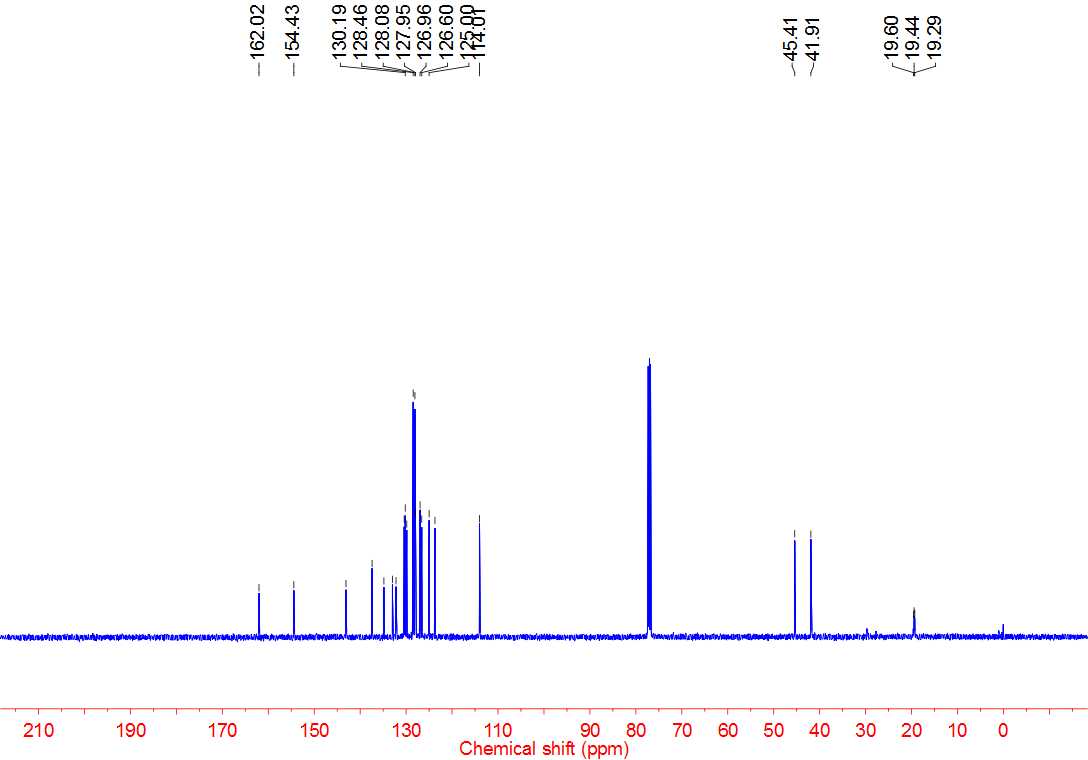
**

**4m ^1^H NMR**

**
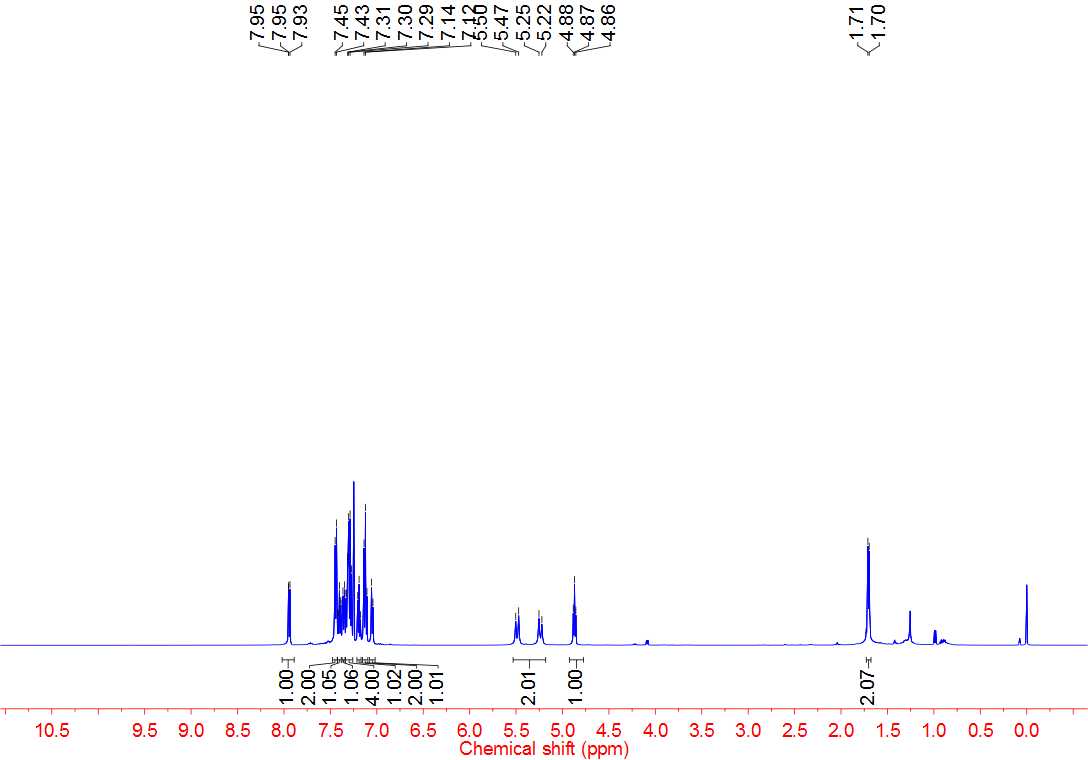
**

**4m ^13^C NMR**

**
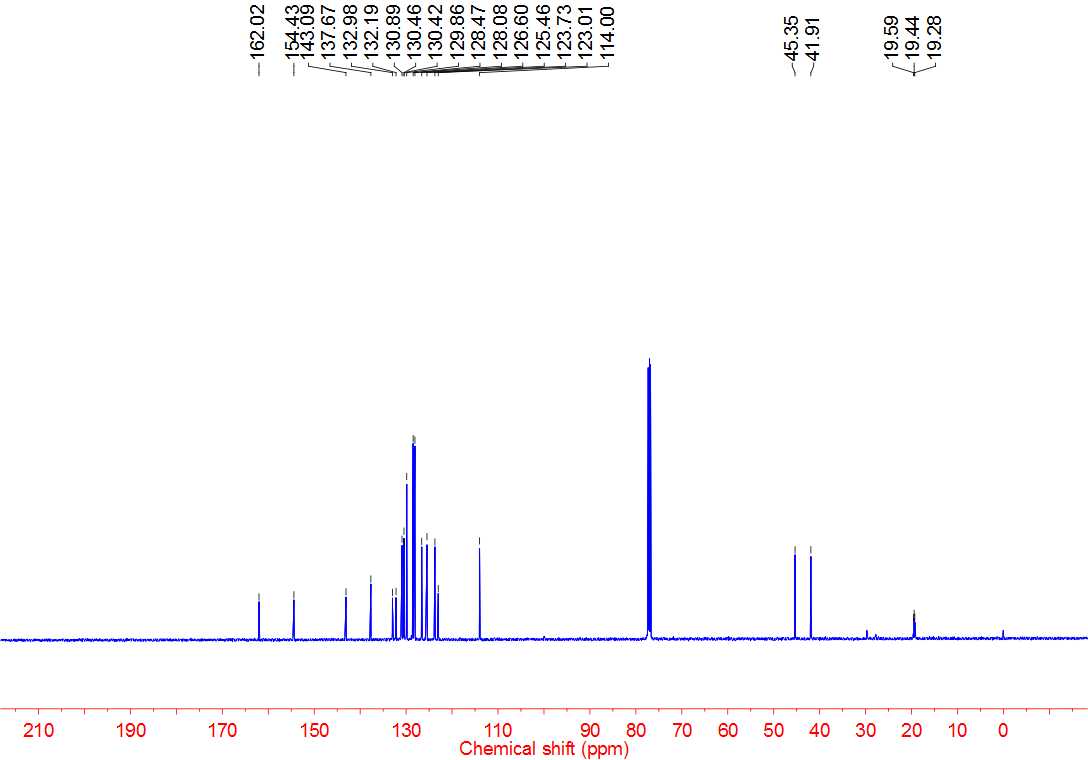
**

**4n ^1^H NMR**


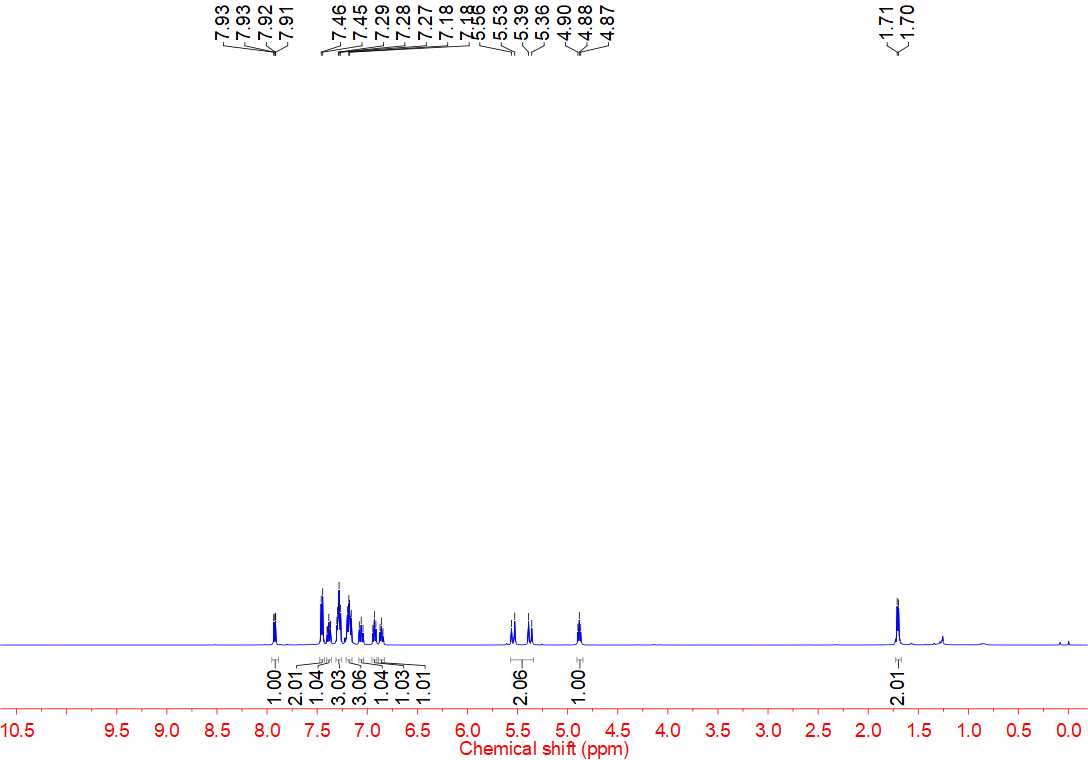

**4n ^13^C NMR**

**
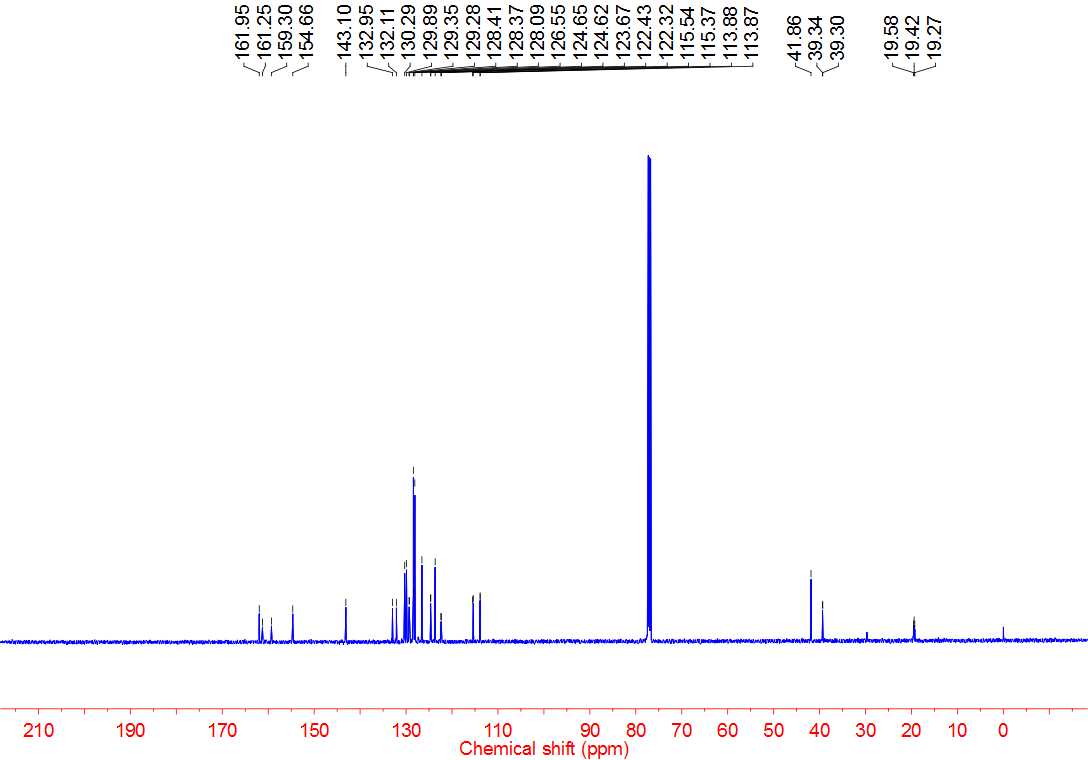
**

**4n ^19^F NMR**

**
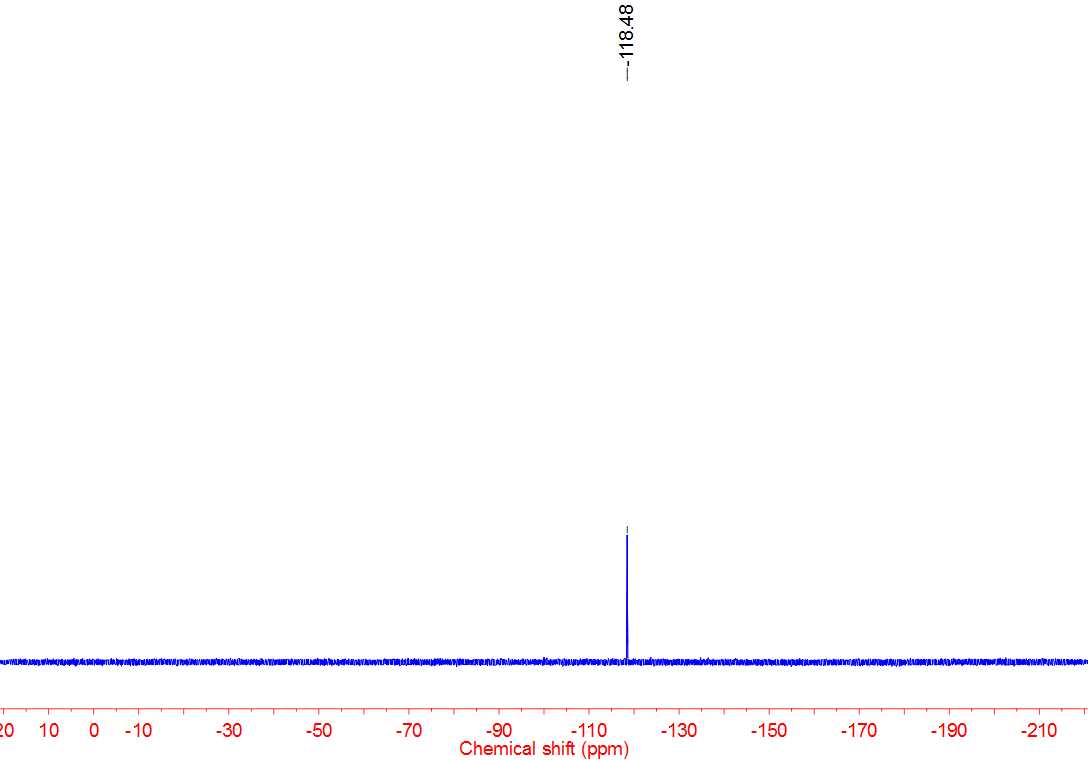
**

**4o ^1^H NMR**

**
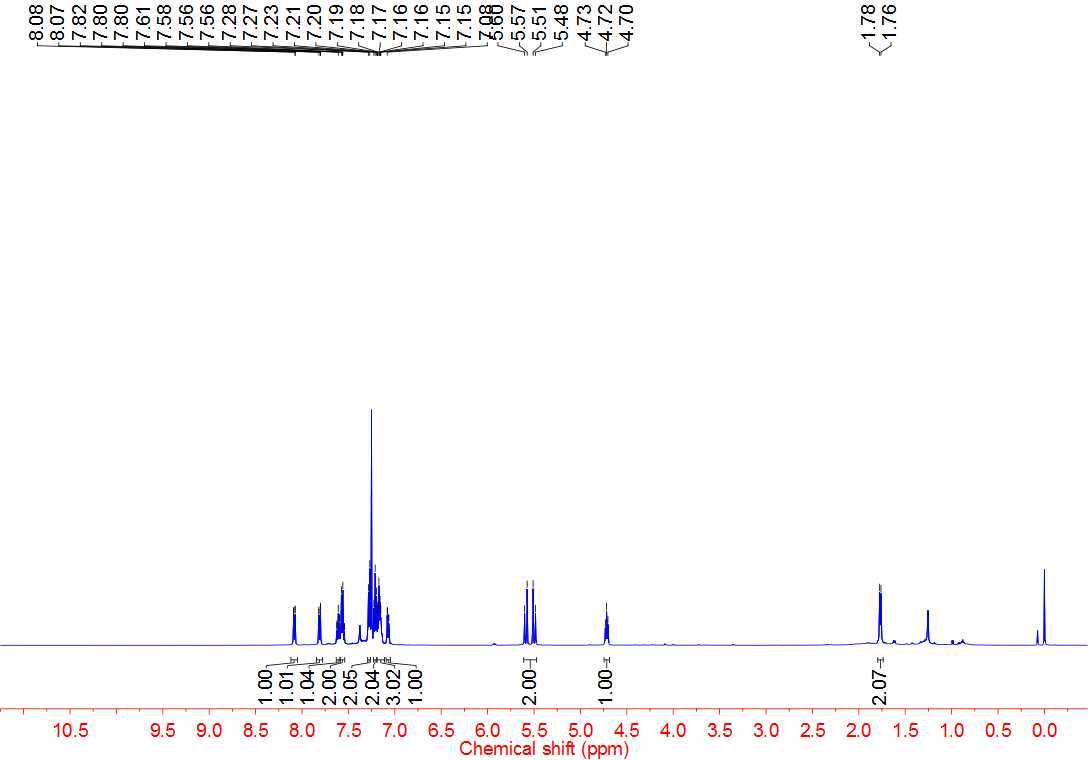
**

**4o ^13^C NMR**


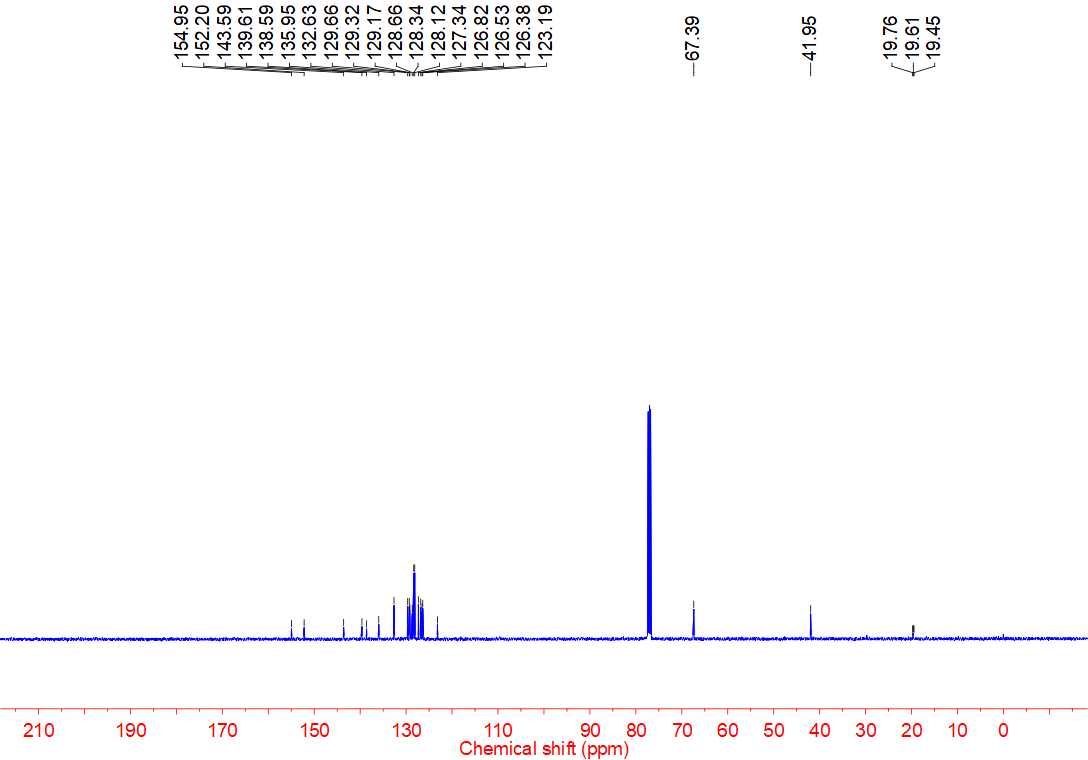

**4p ^1^H NMR**


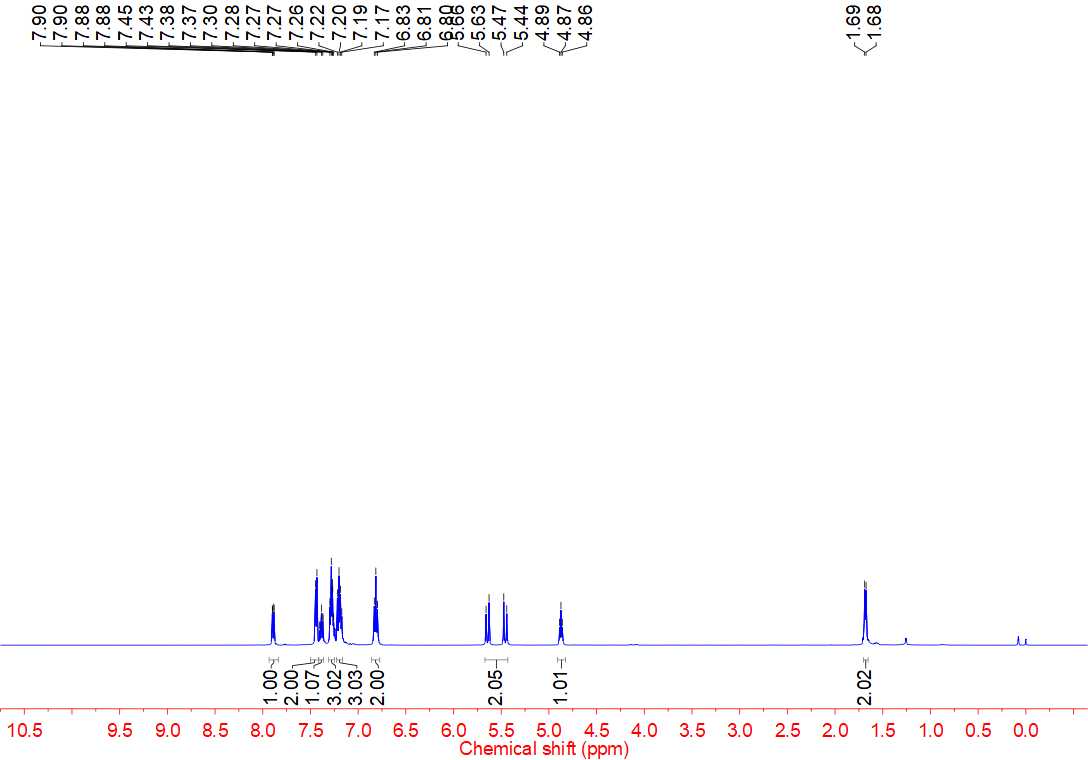

**4p ^13^C NMR**

**
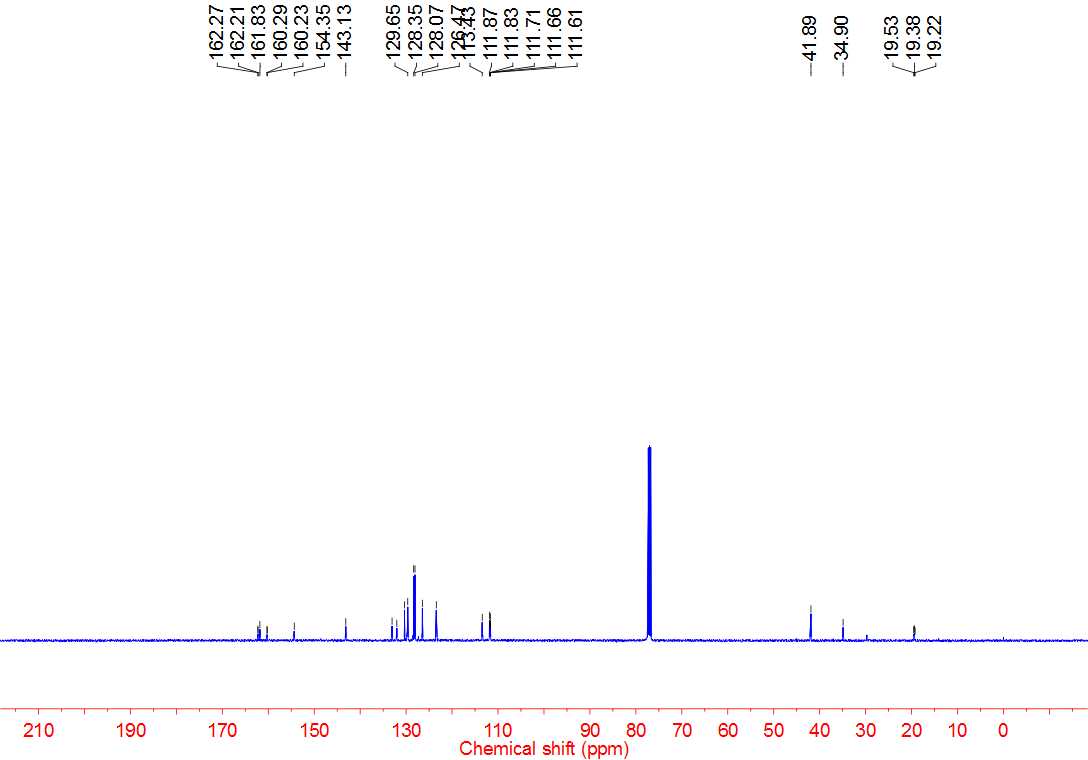
**

**4p ^19^F NMR**

**
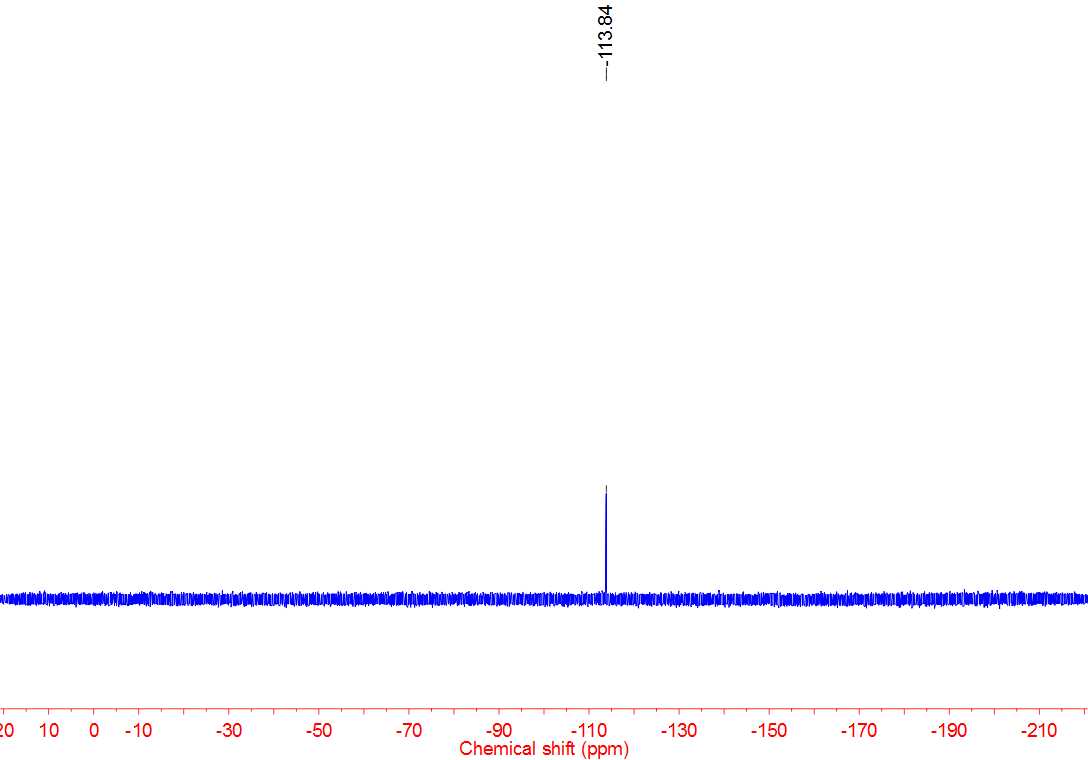
**

**4q ^1^H NMR**


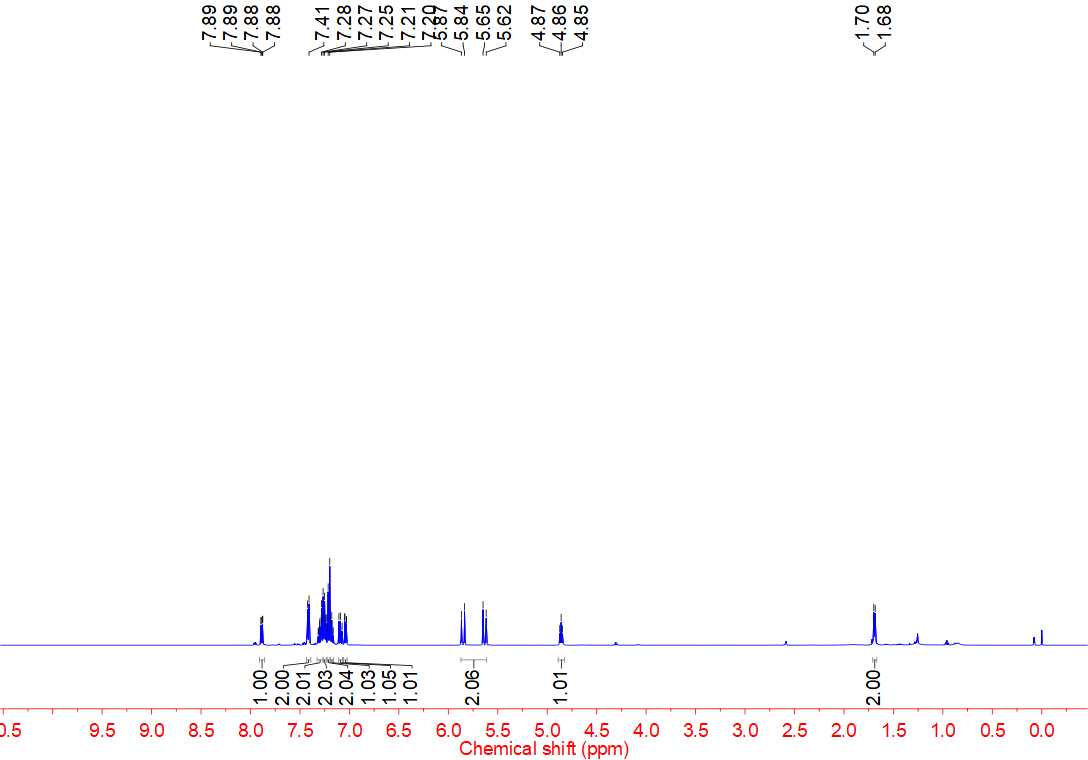

**4q ^13^C NMR**


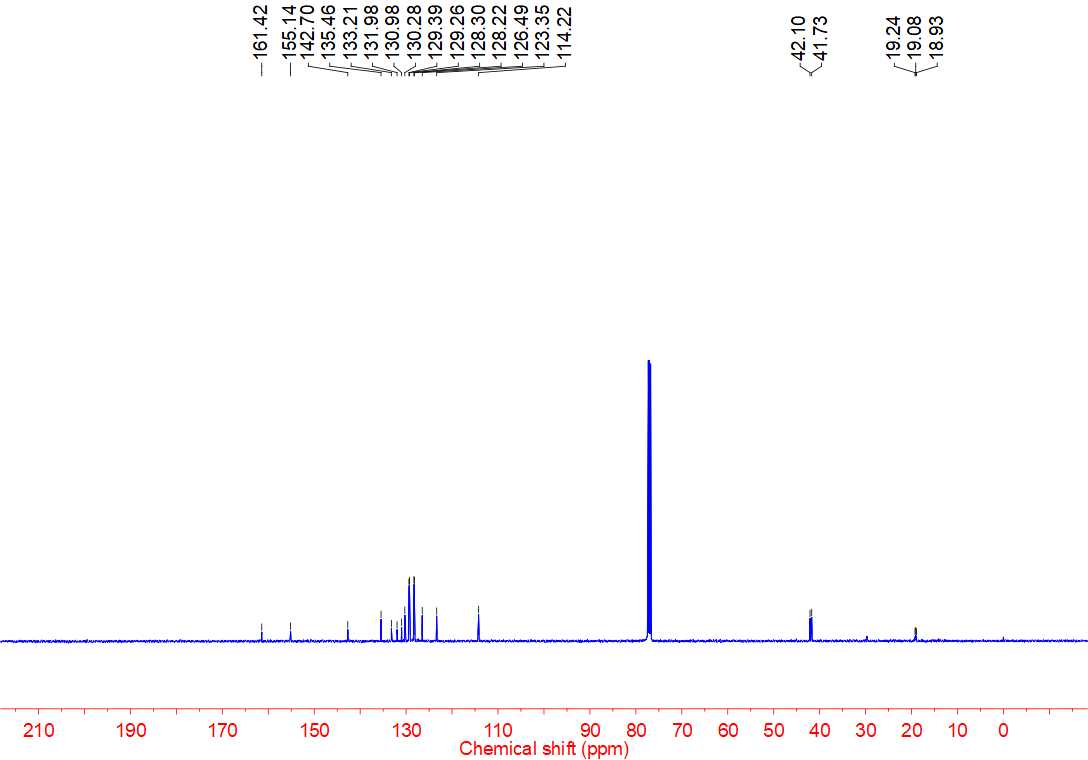

**4r ^1^H NMR**

**
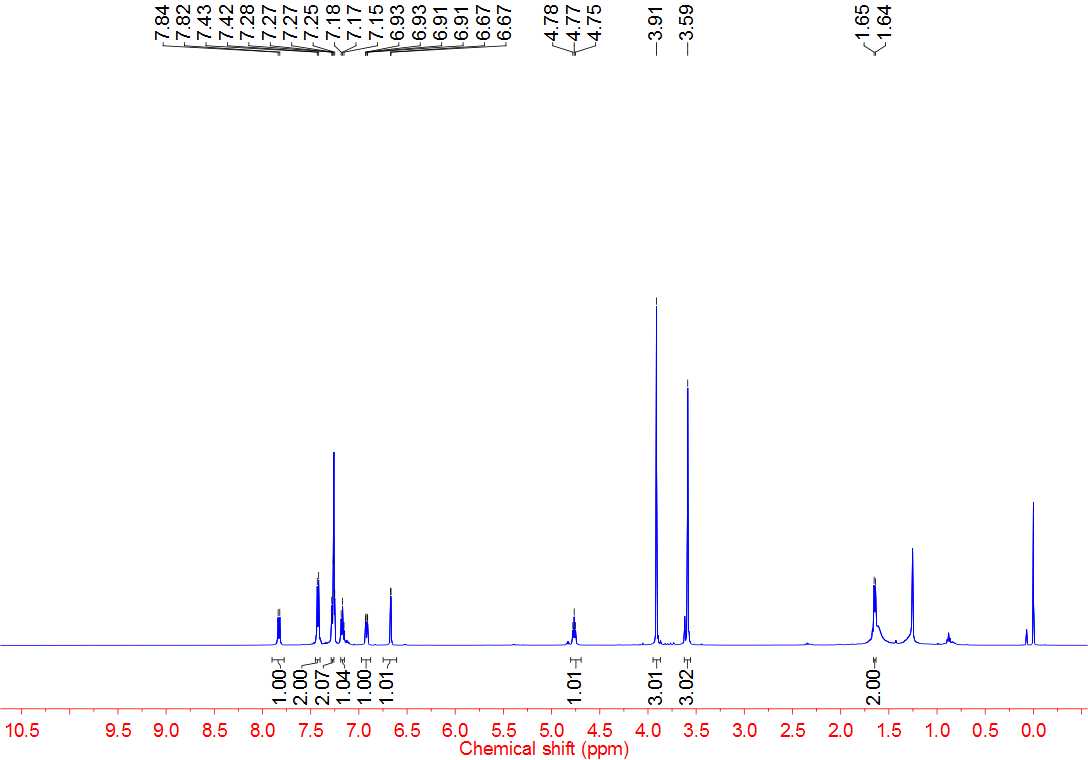
**

**4r ^13^C NMR**


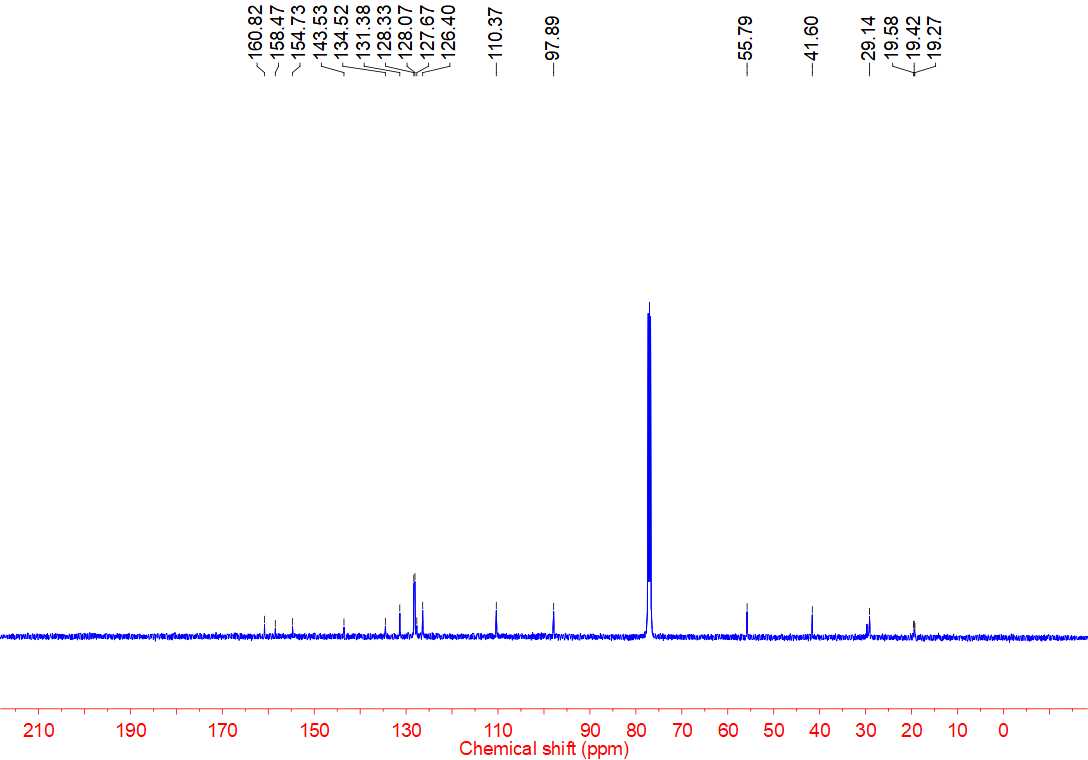

**4s ^1^H NMR**

**
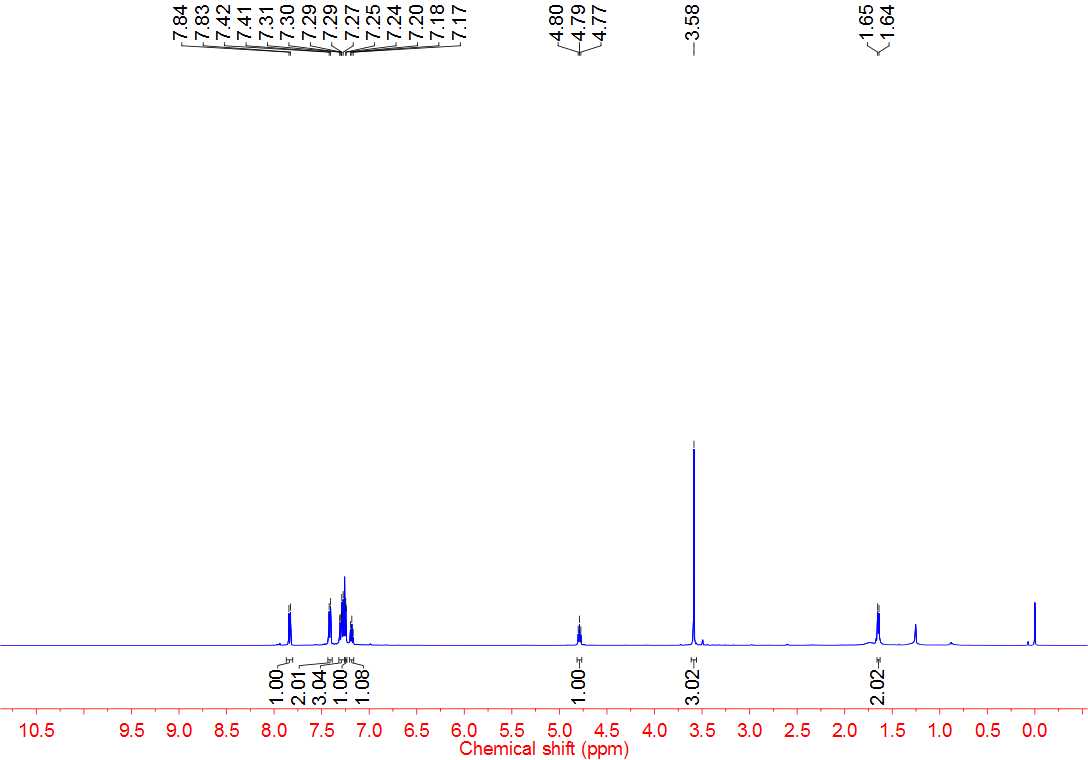
**

**4s ^13^C NMR**

**
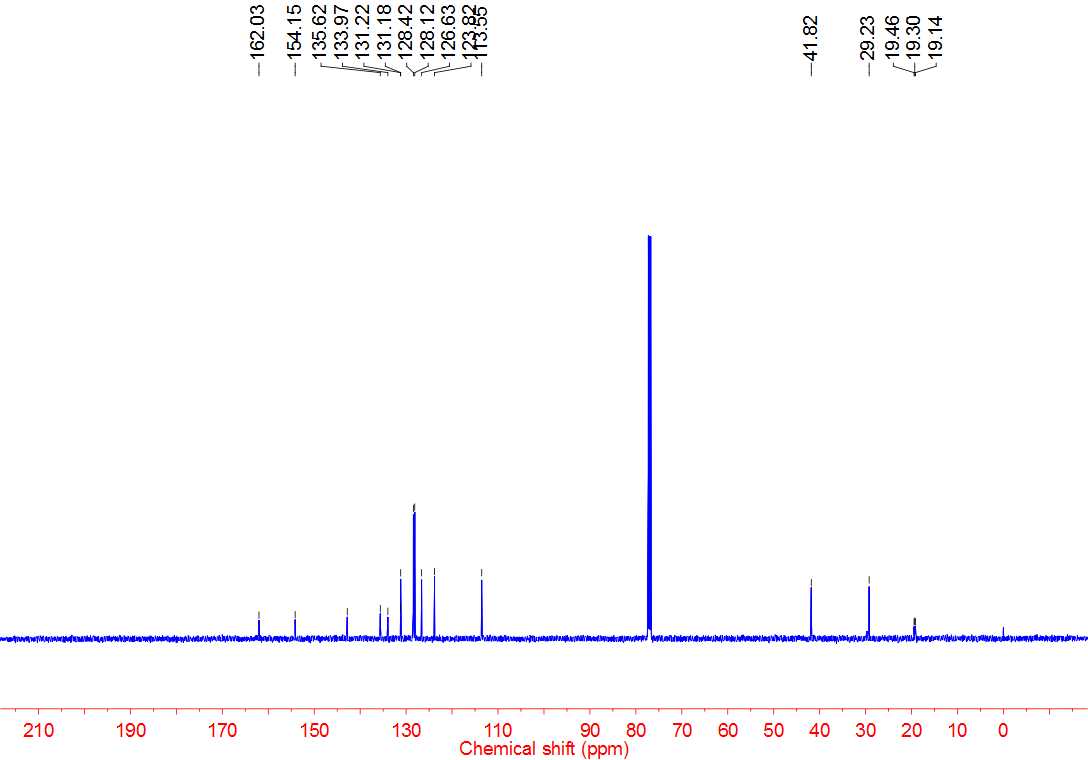
**

**4t ^1^H NMR**

**
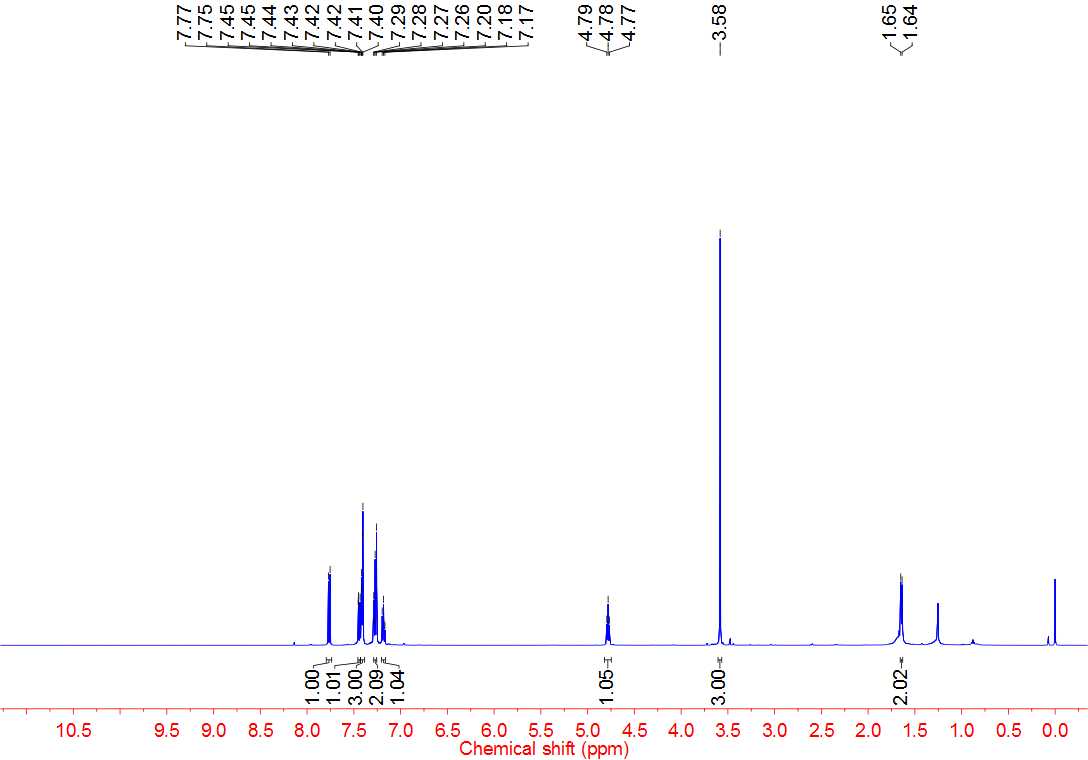
**

**4t ^13^C NMR**

**
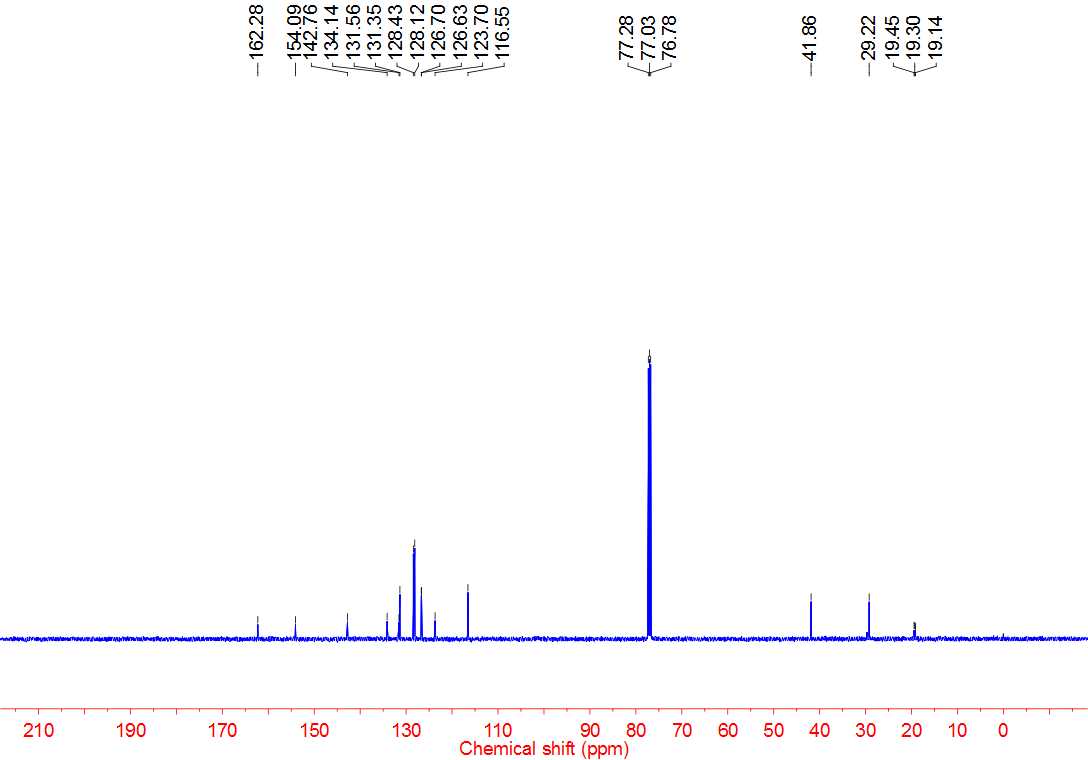
**

**4u ^1^H NMR**


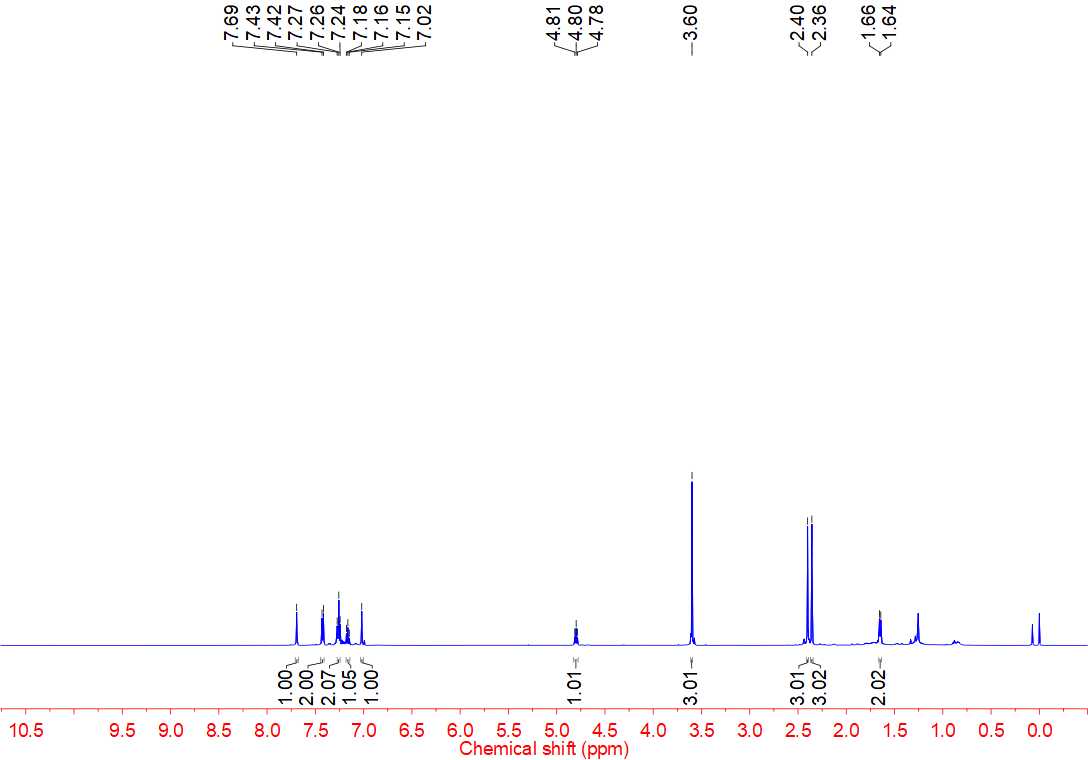

**4u ^13^C NMR**

**
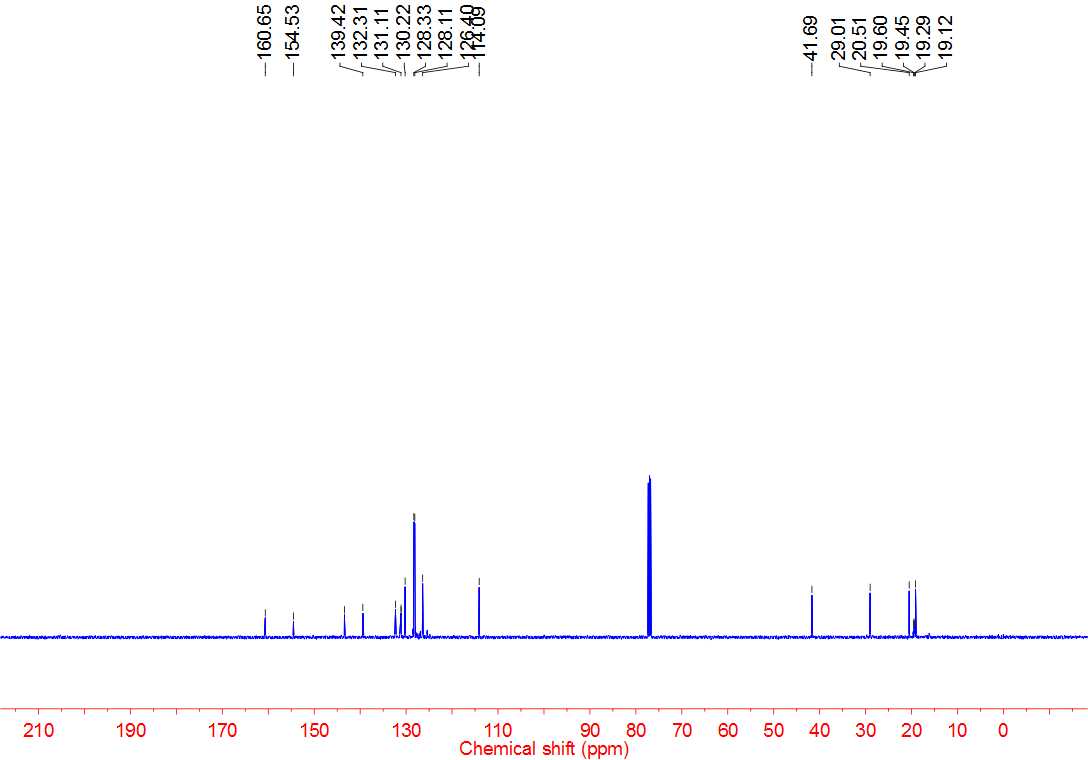
**

**4v ^1^H NMR**

**
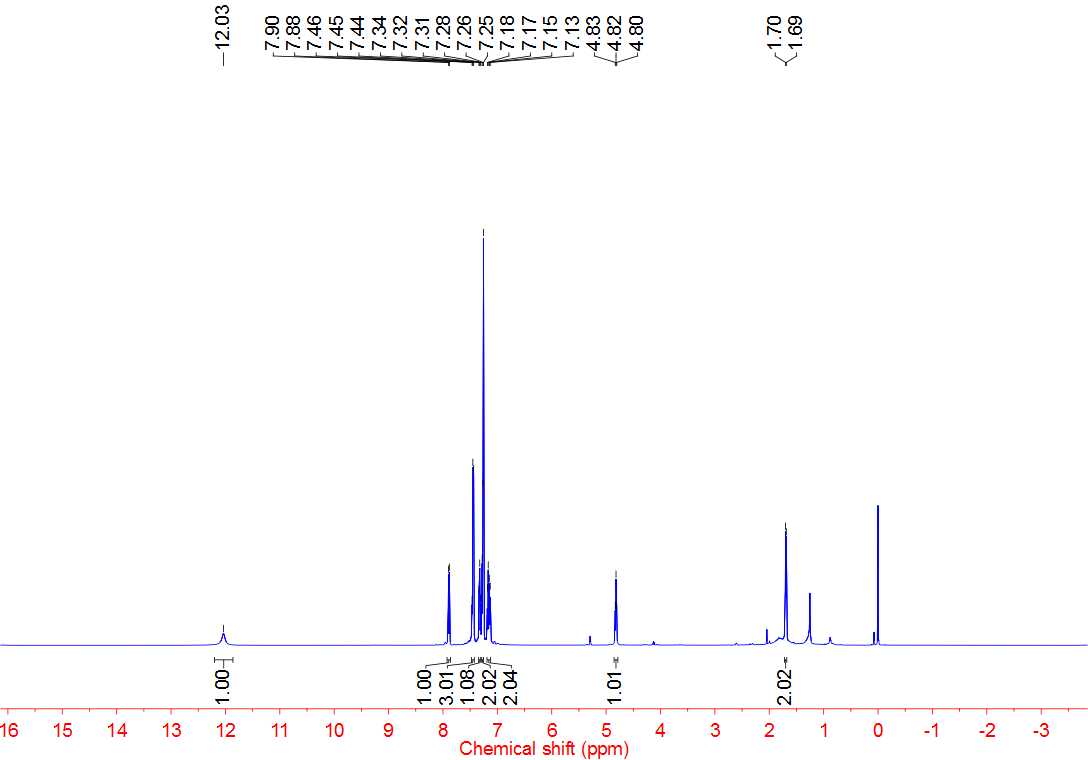
**

**4v ^13^C NMR**

**
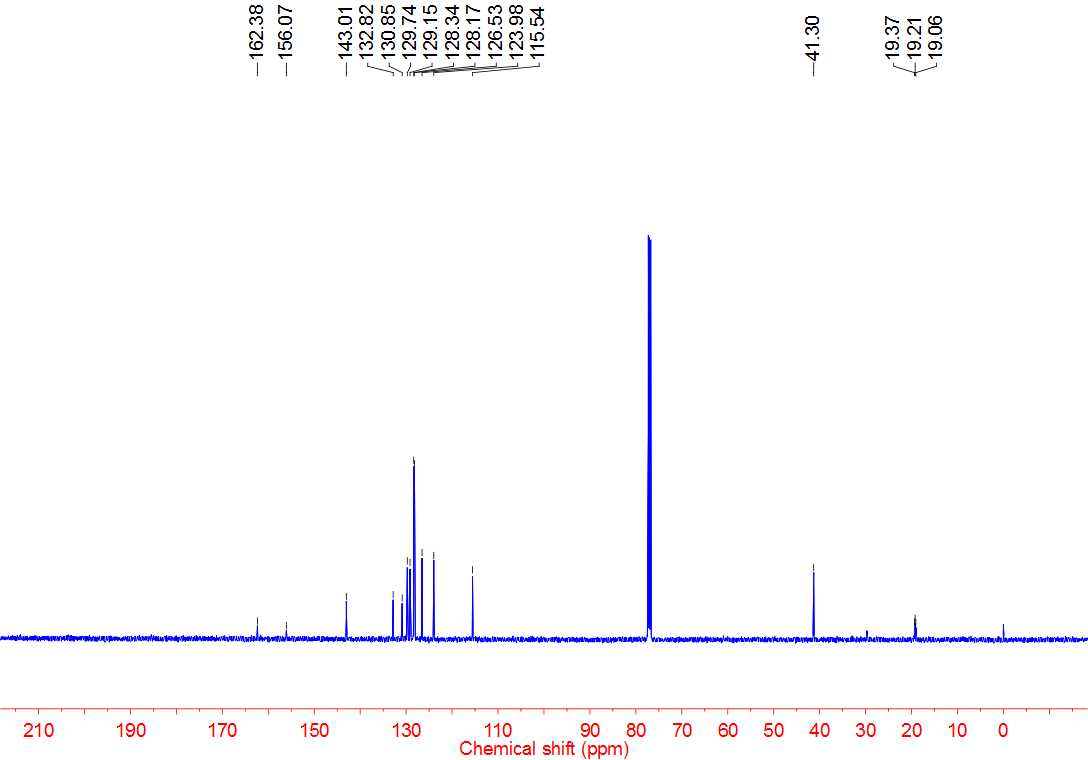
**

**4aa ^1^H NMR**

**
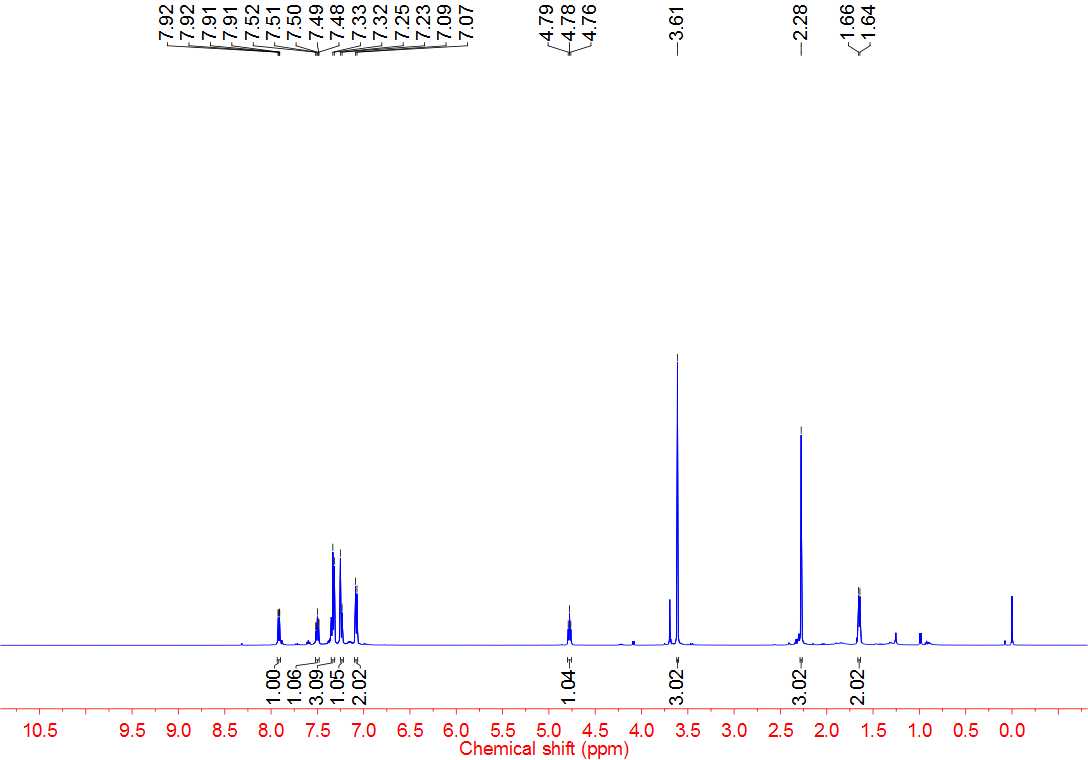
**

**4aa ^13^C NMR**

**
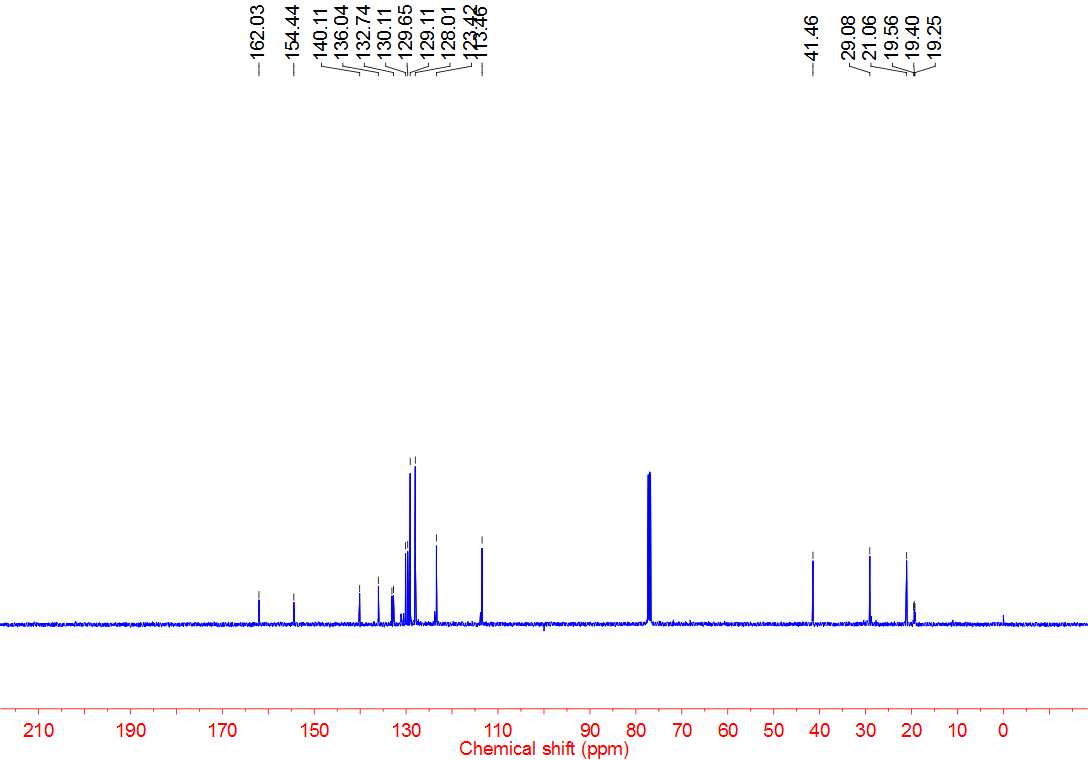
**

**4ab ^1^H NMR**


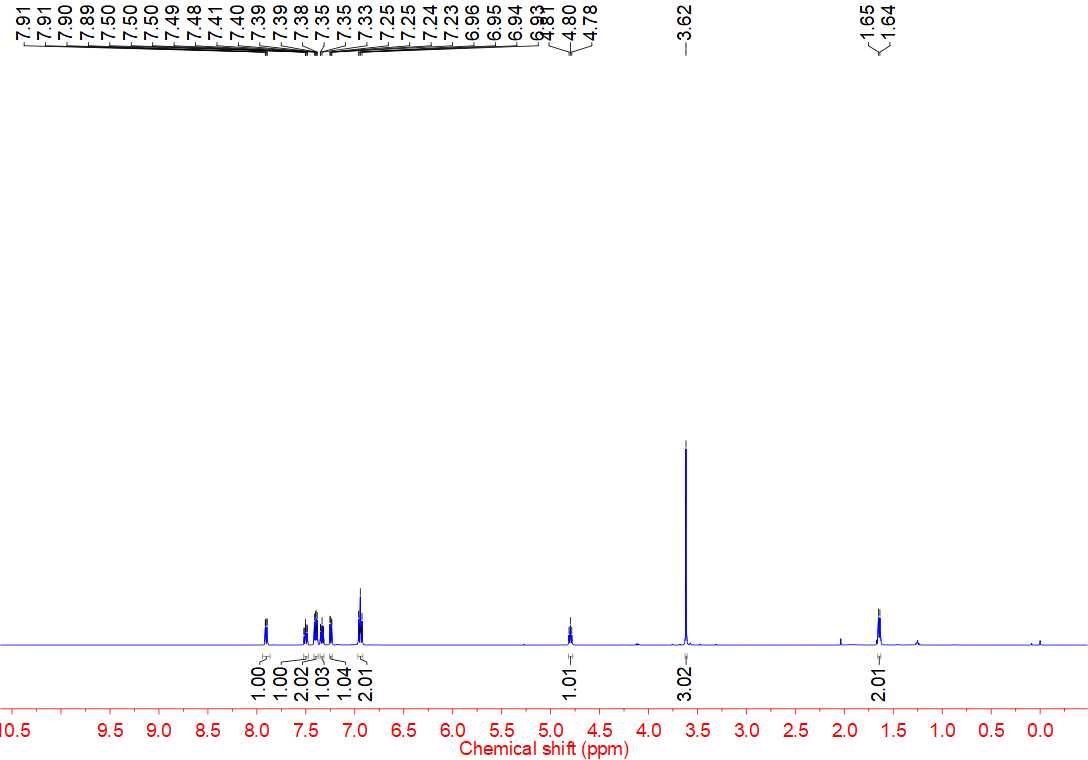

**4ab ^13^C NMR**

**
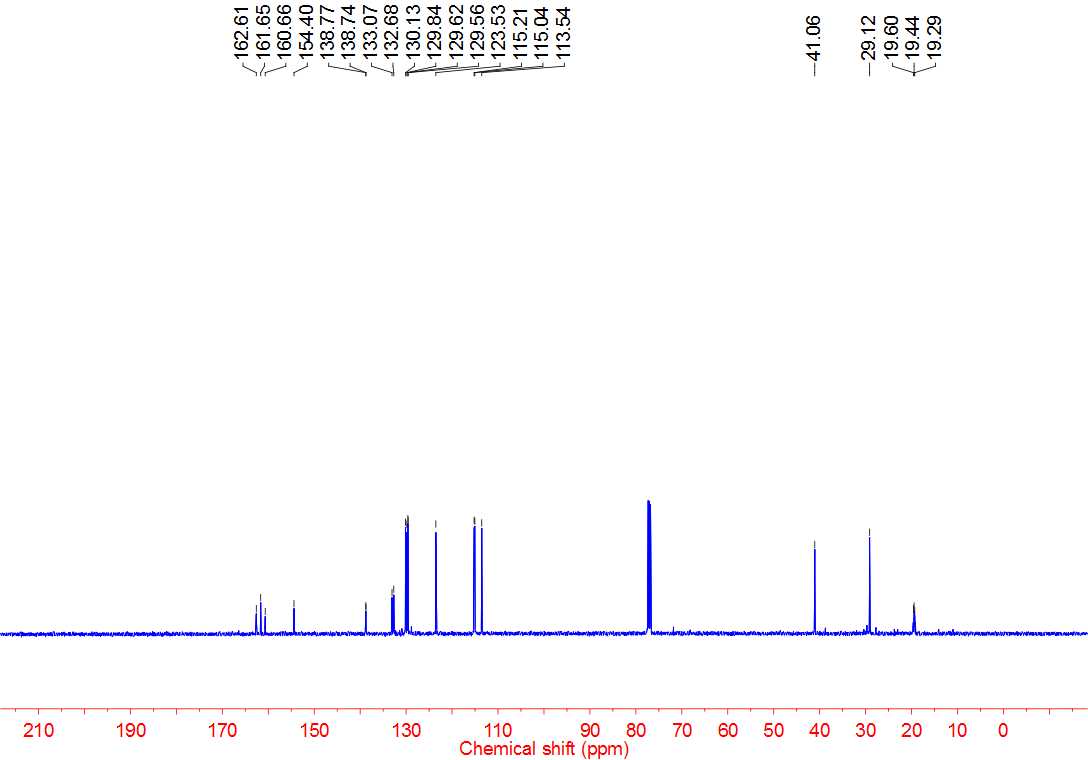
**

**4ab ^19^F NMR**

**
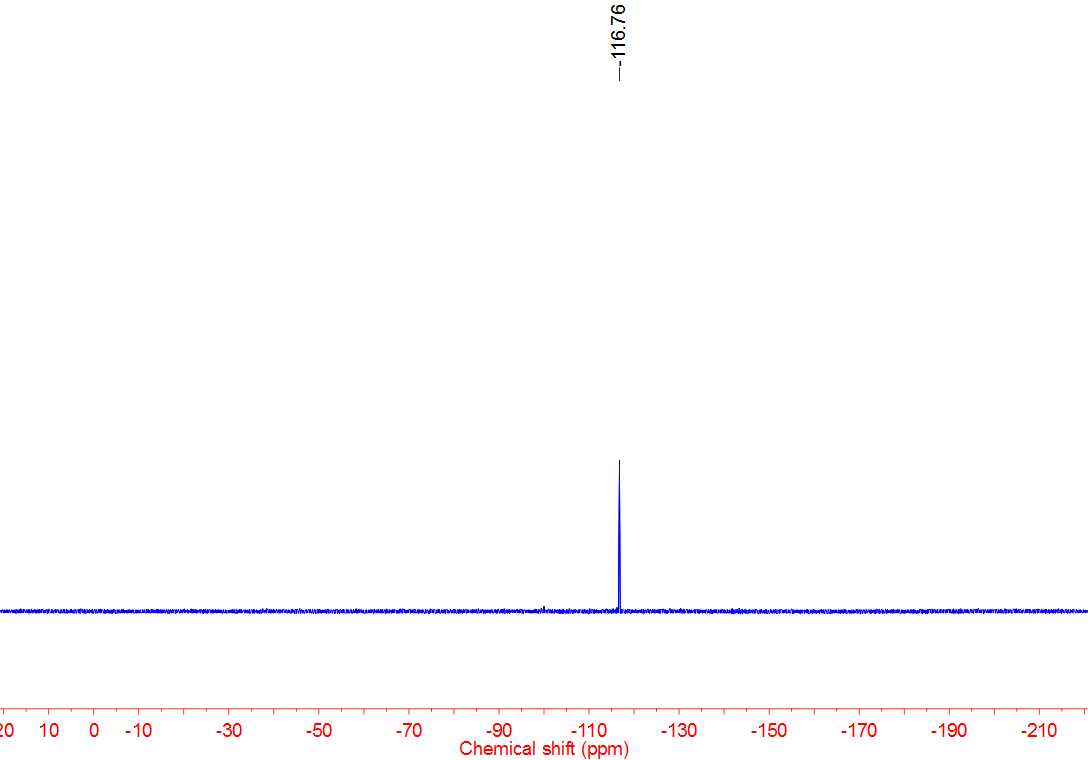
**

**4ac ^1^H NMR**

**
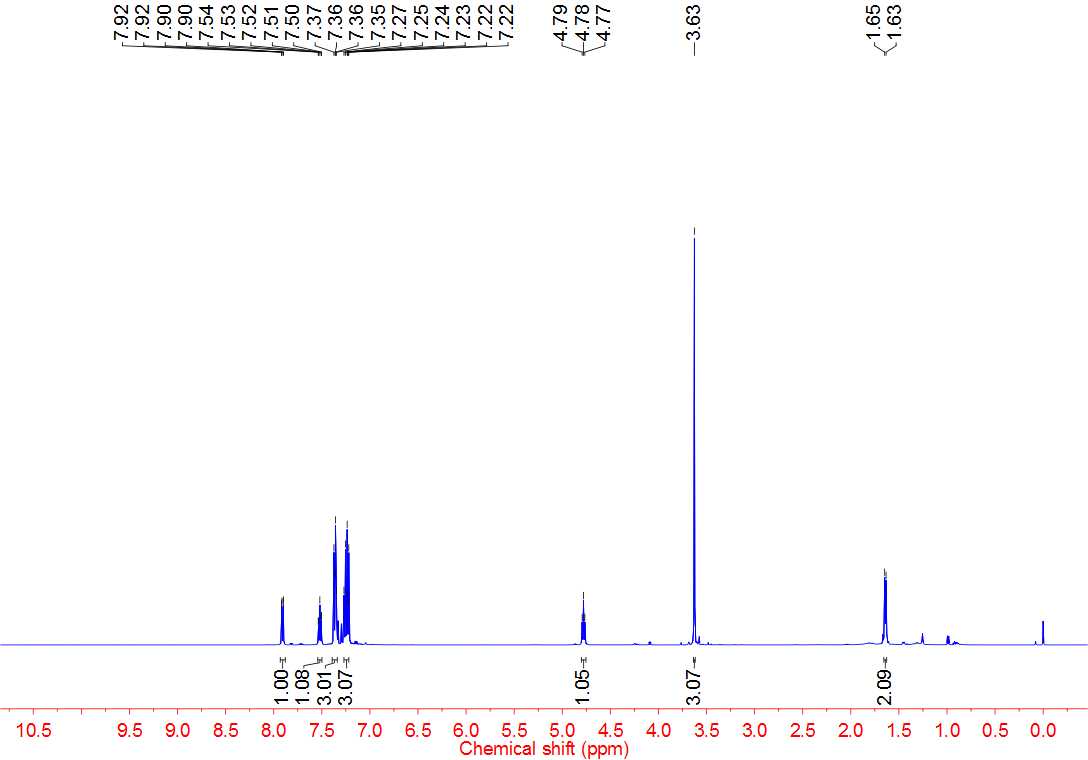
**

**4ac ^13^C NMR**

**
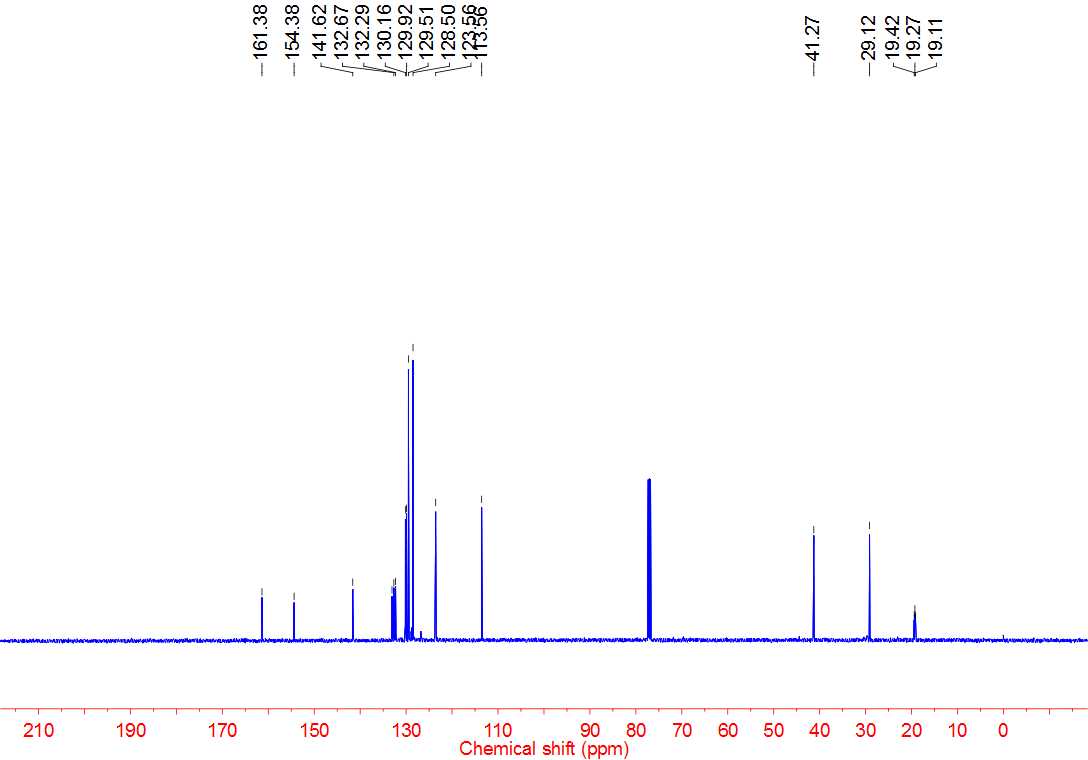
**

**4ad ^1^H NMR**

**
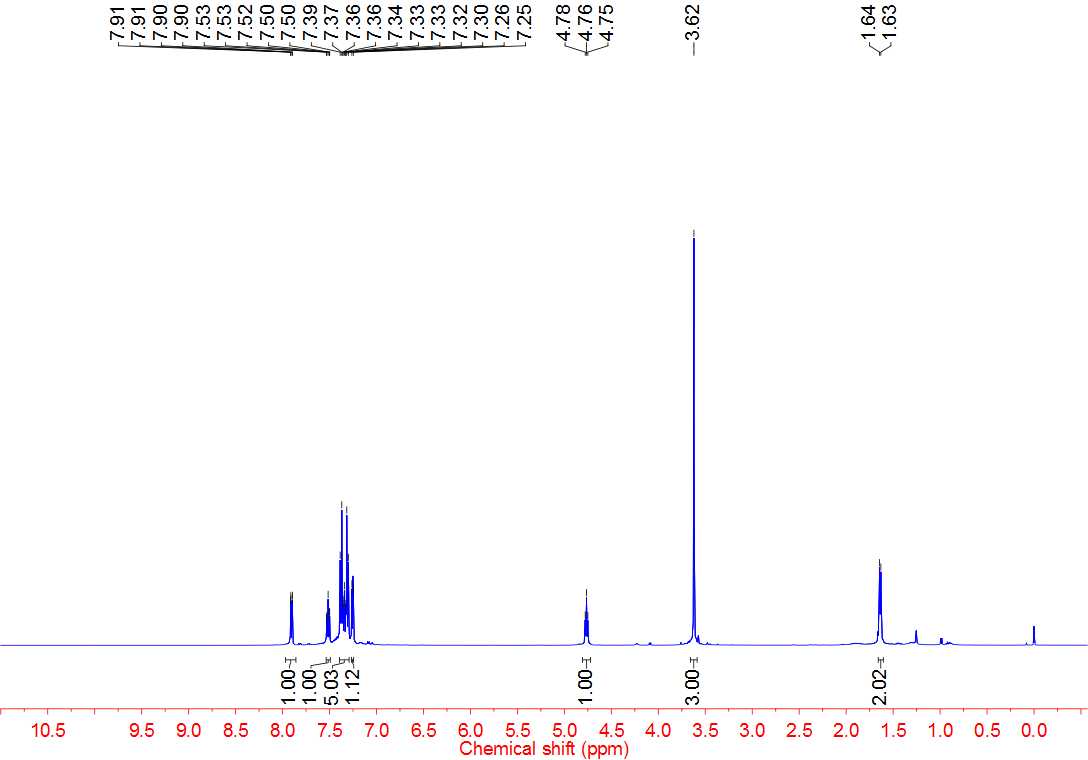
**

**4ad ^13^C NMR**

**
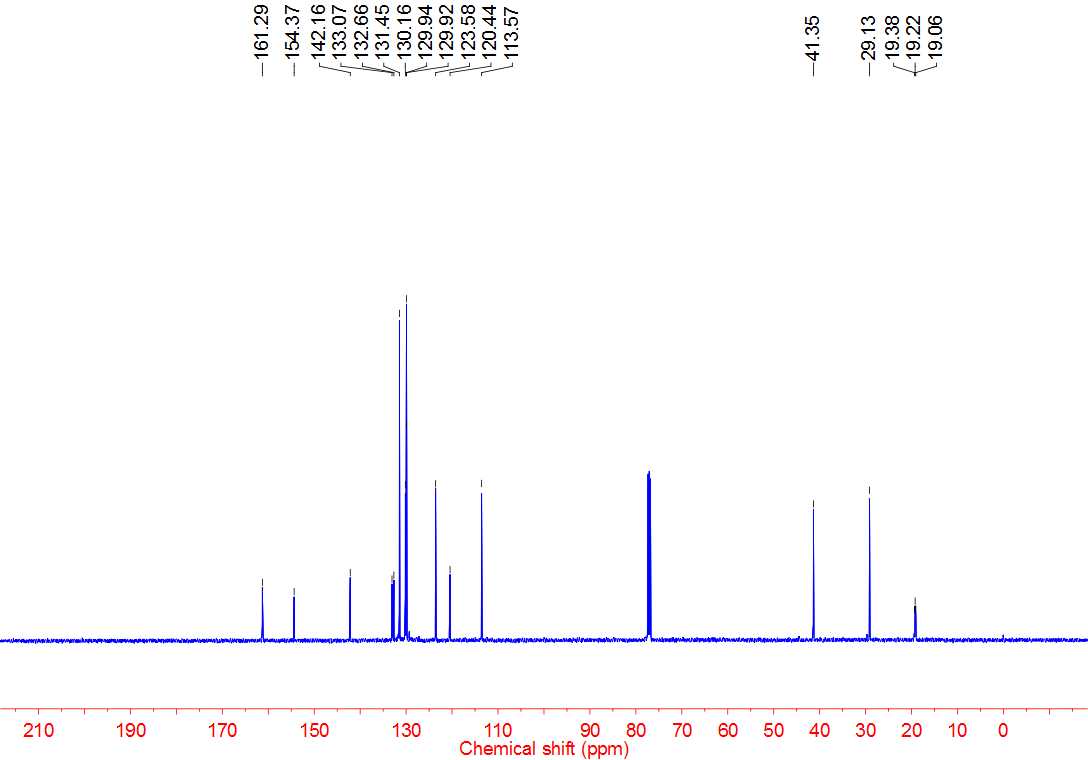
**

**4ae ^1^H NMR**

**
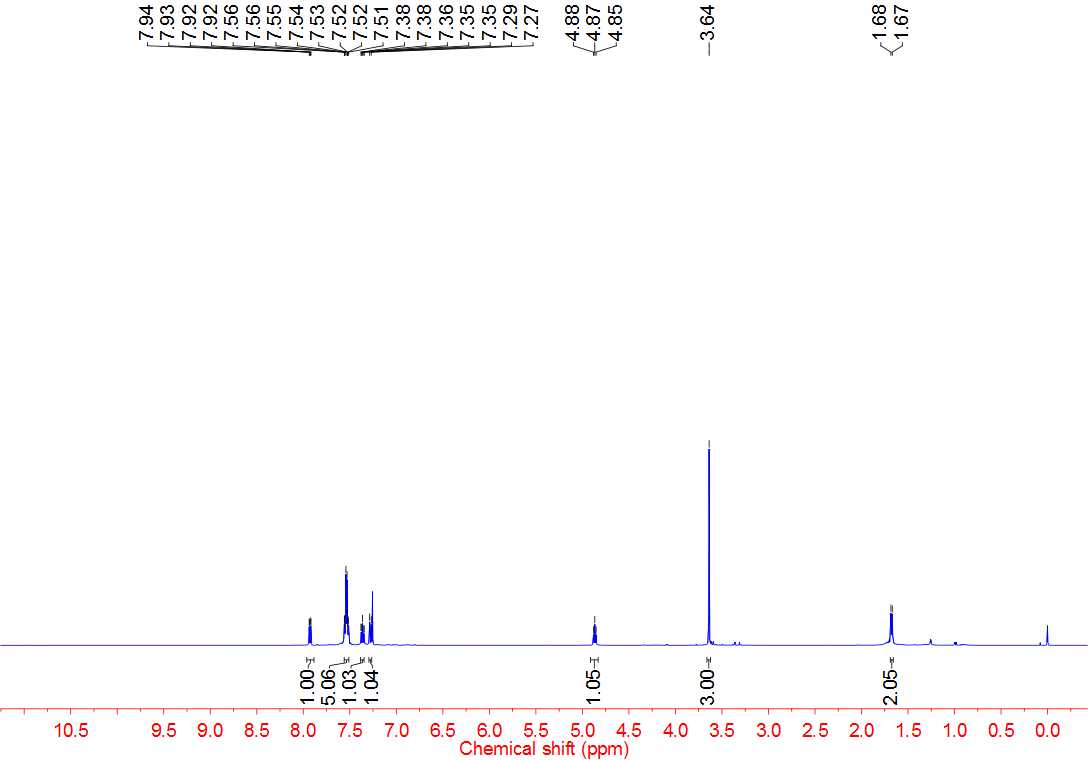
**

**4ae ^13^C NMR**

**
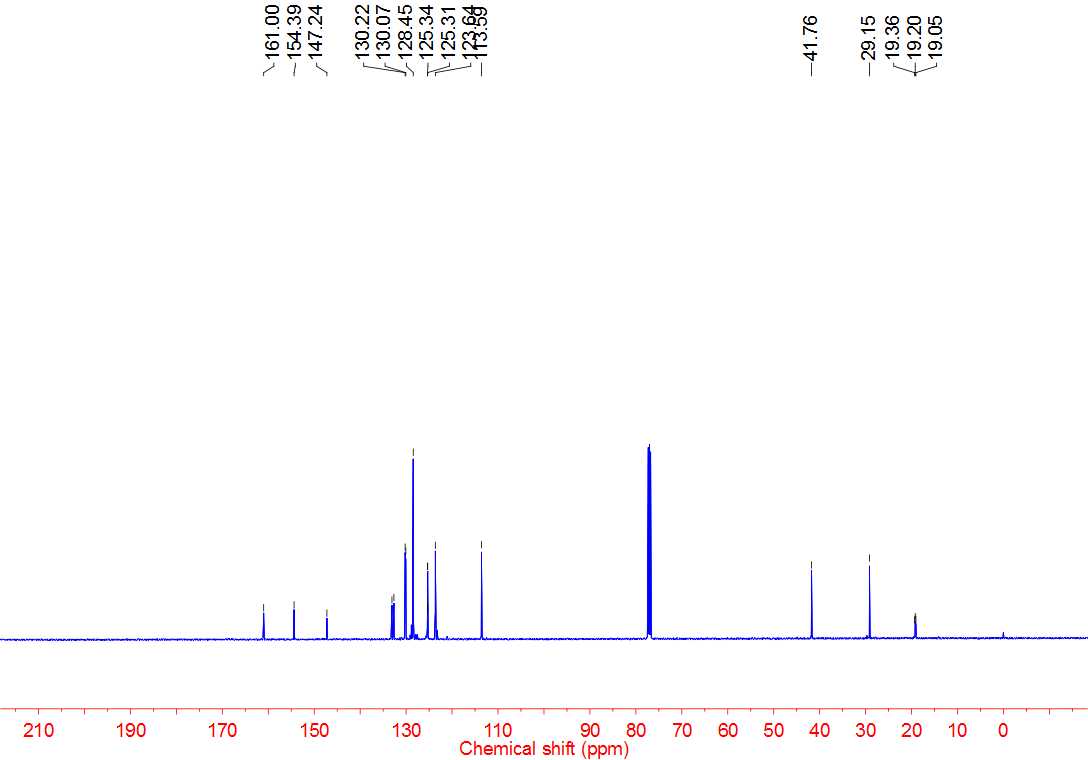
**

**4ae ^19^F NMR**

**
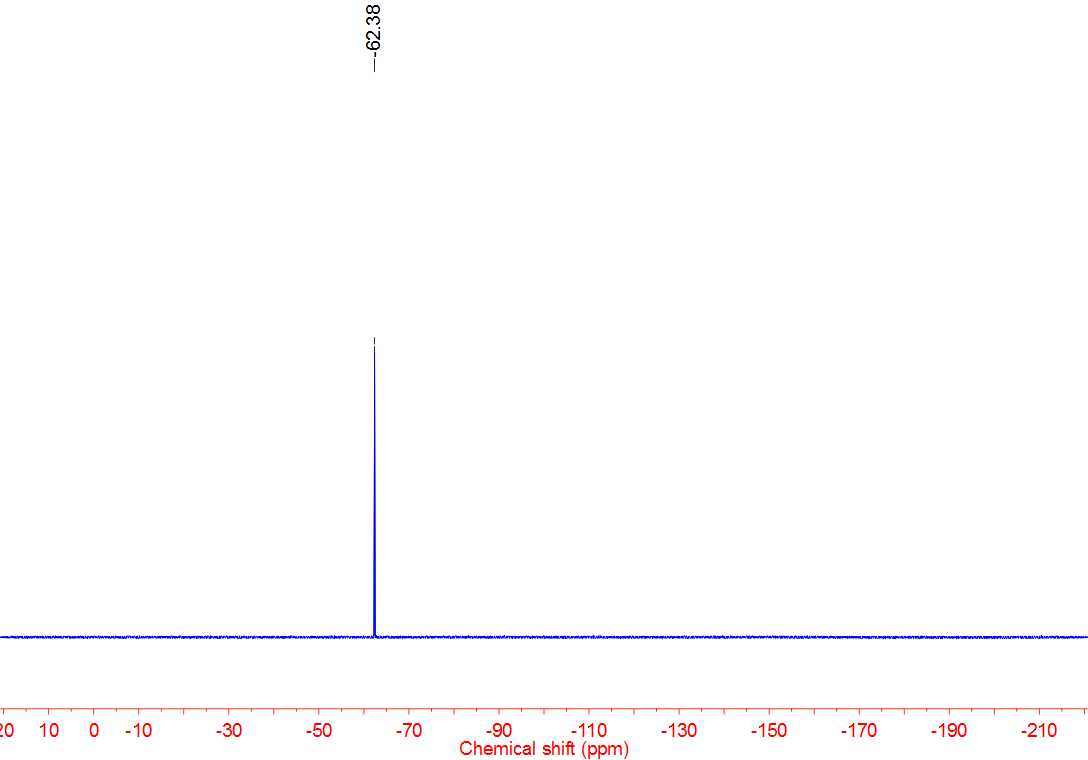
**

**4af ^1^H NMR**


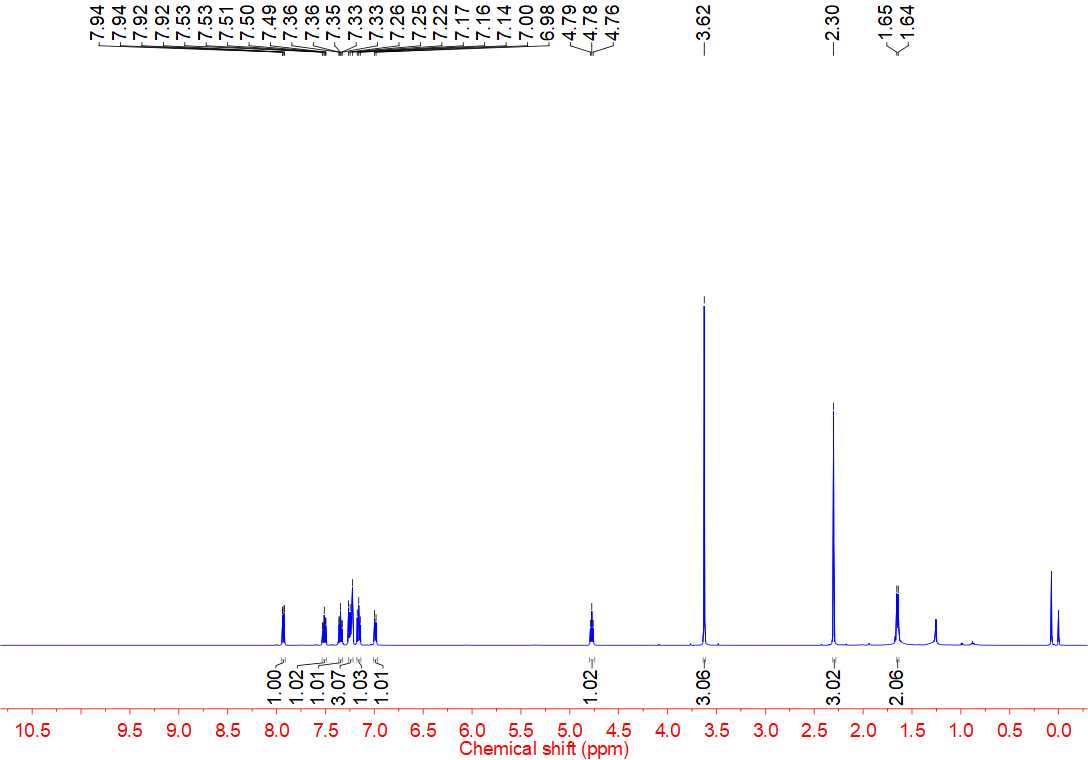


**4af ^13^C NMR**


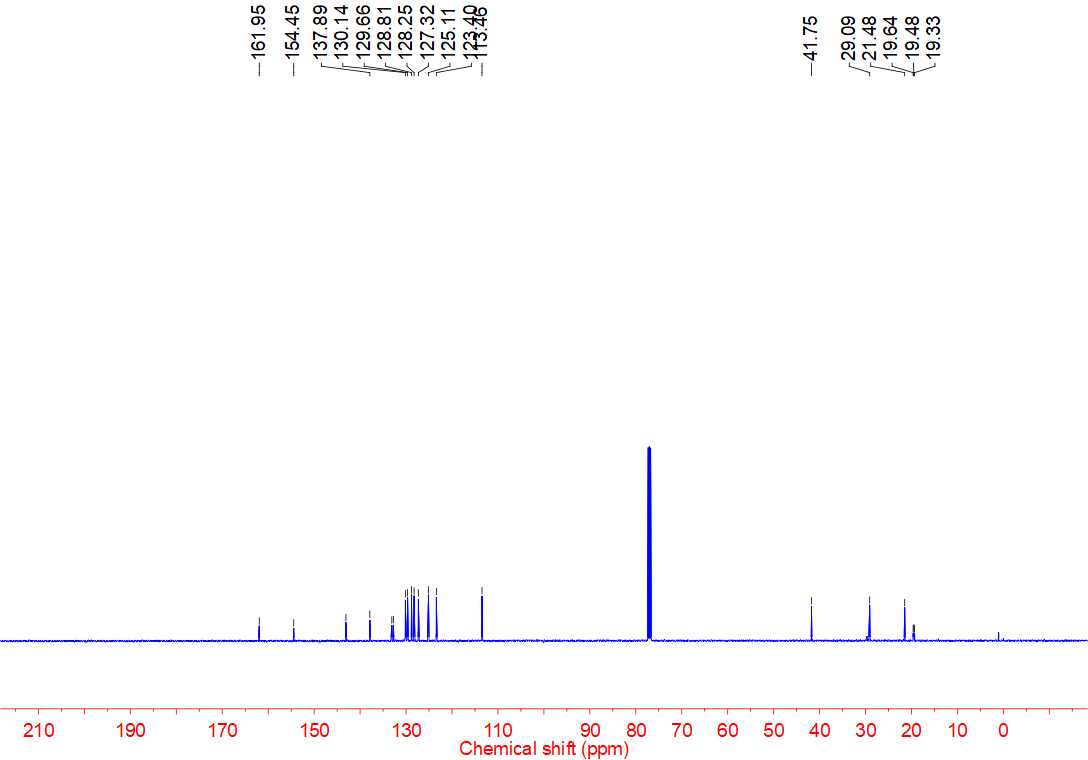


**4ag ^1^H NMR**

**
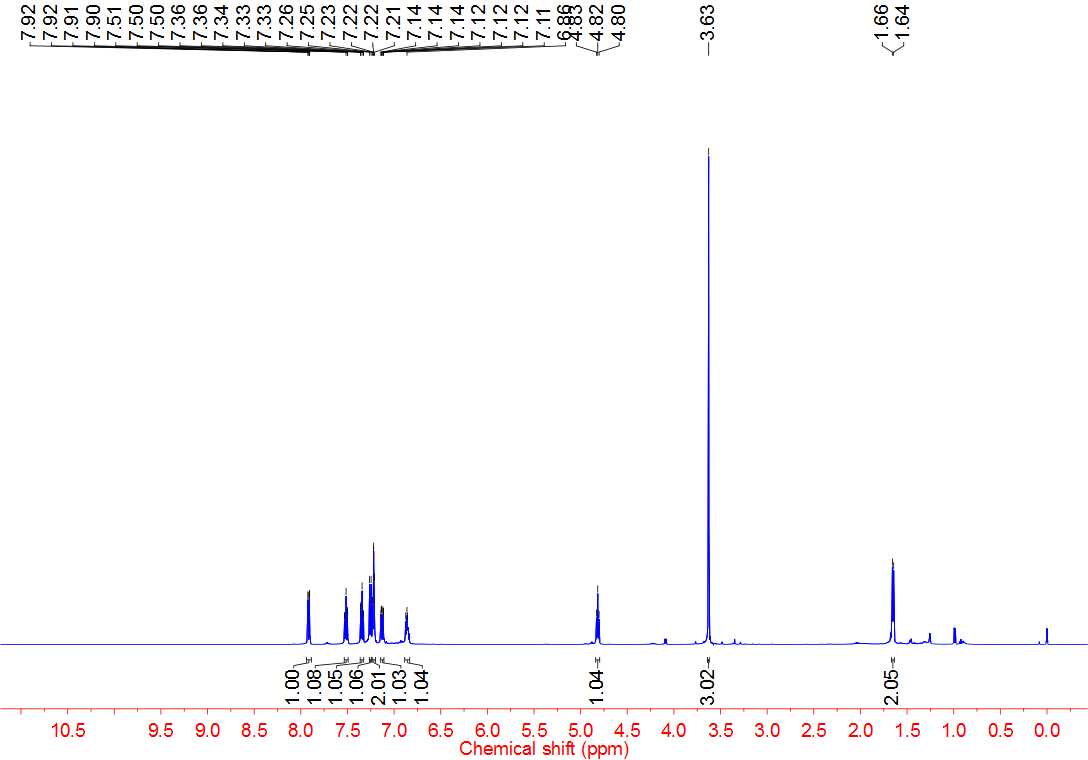
**

**4ag ^13^C NMR**

**
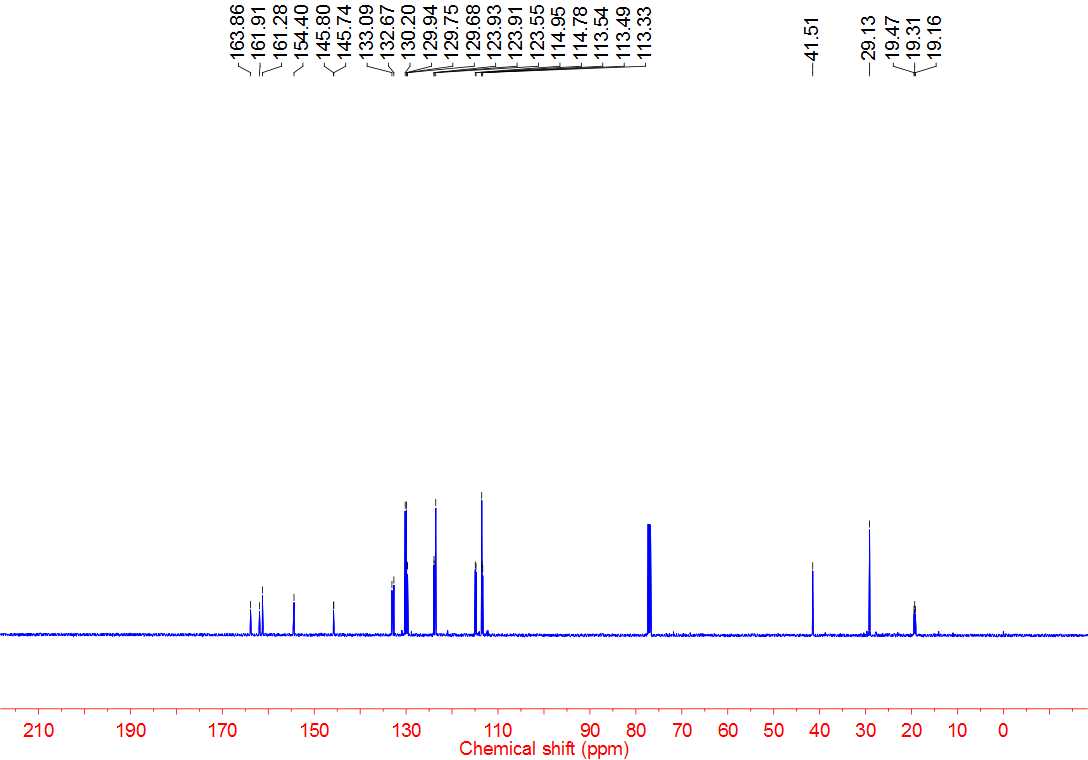
**

**4ag ^19^F NMR**

**
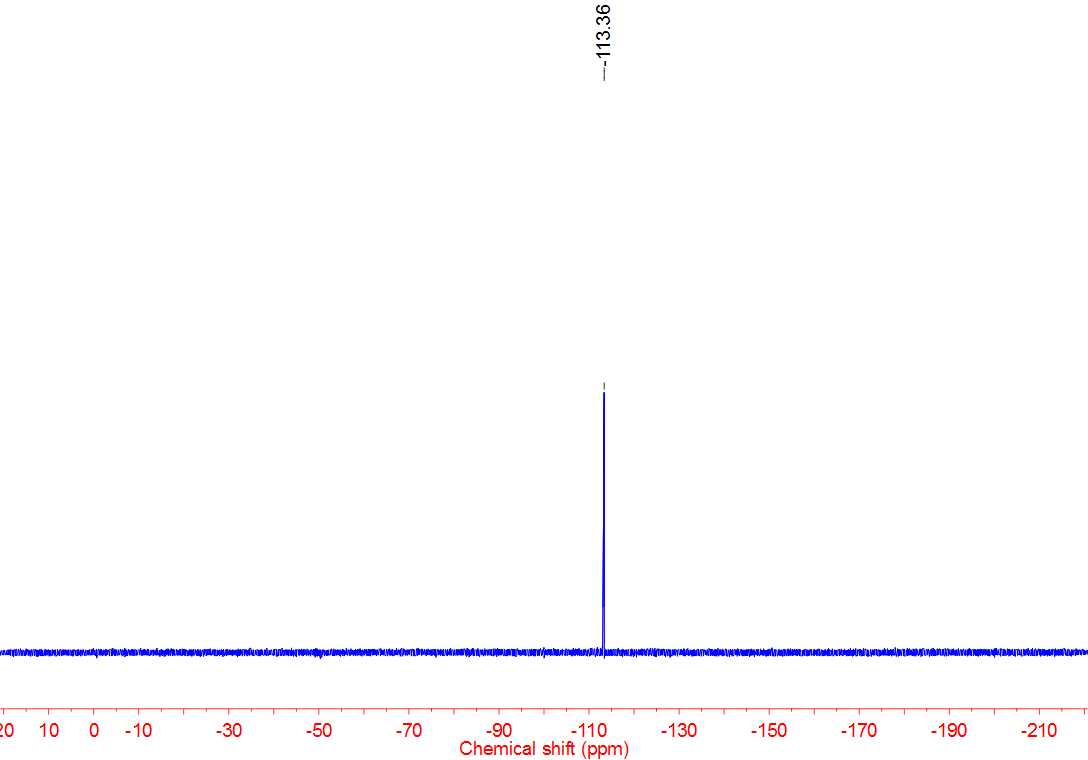
**

**4ah ^1^H NMR**

**
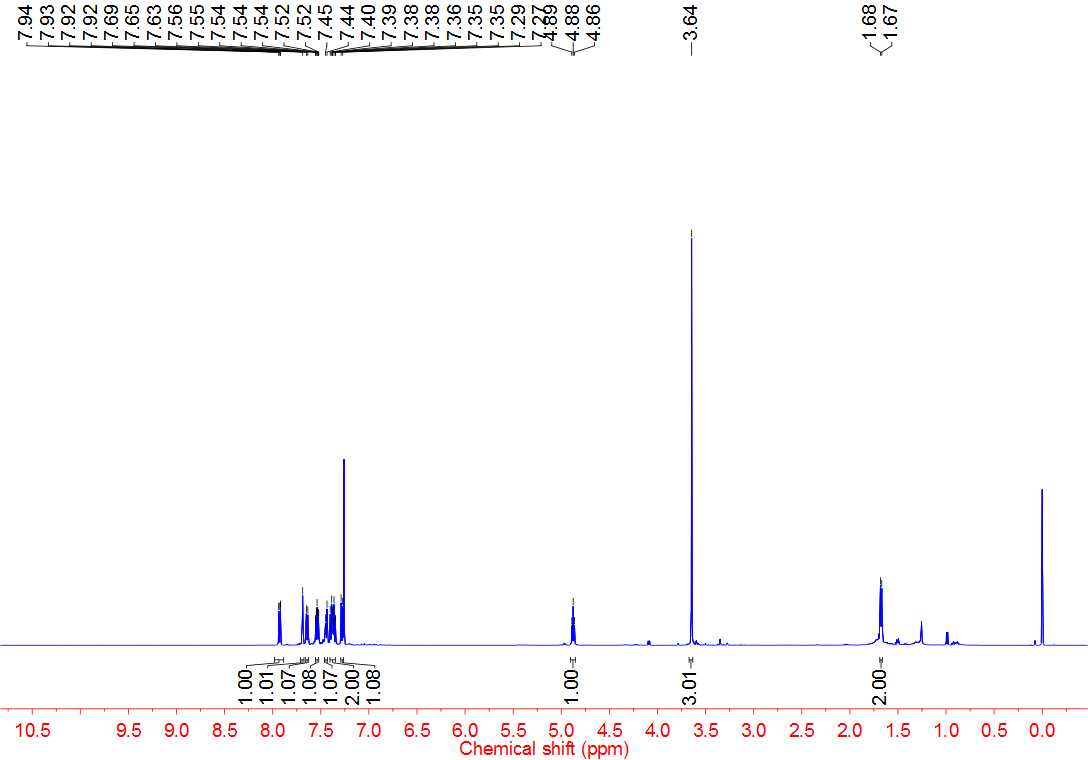
**

**4ah ^13^C NMR**

**
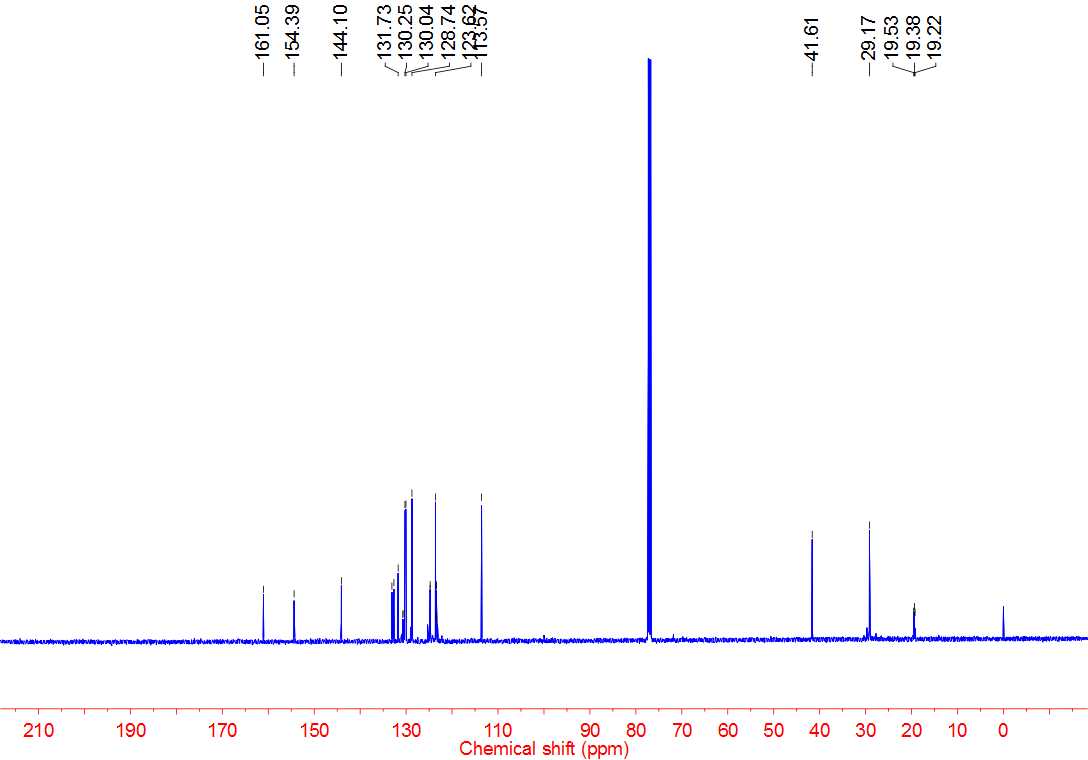
**

**4ah ^19^F NMR**

**
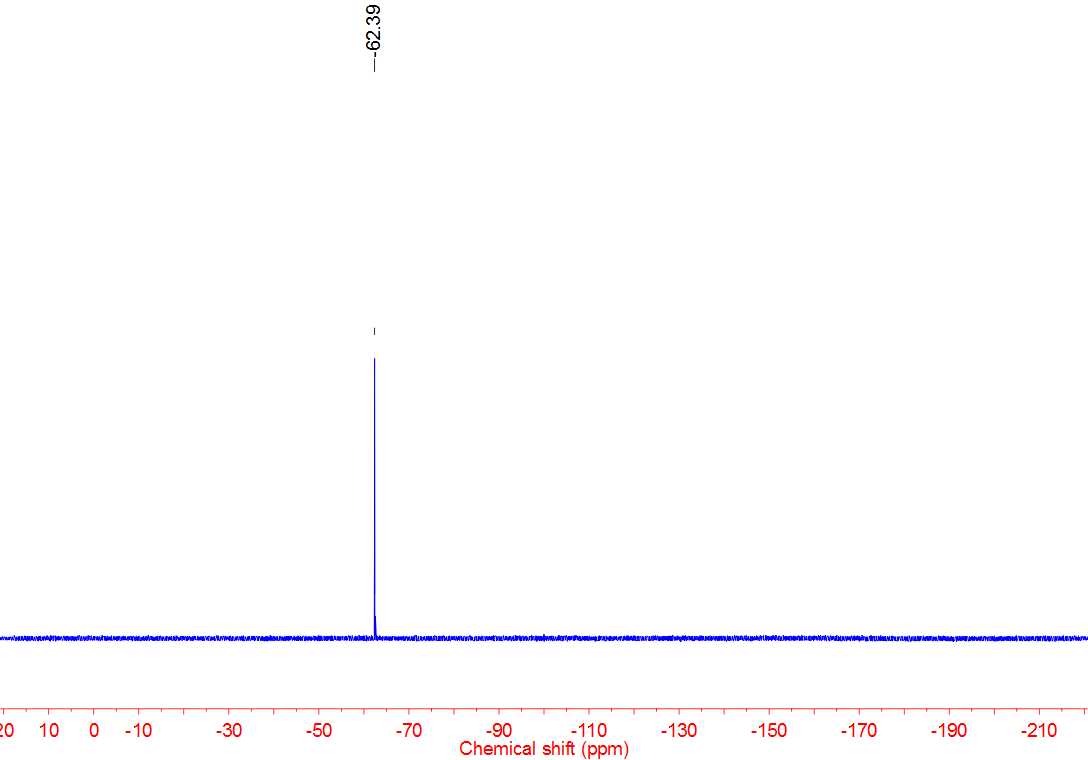
**

**4ai ^1^H NMR**

**
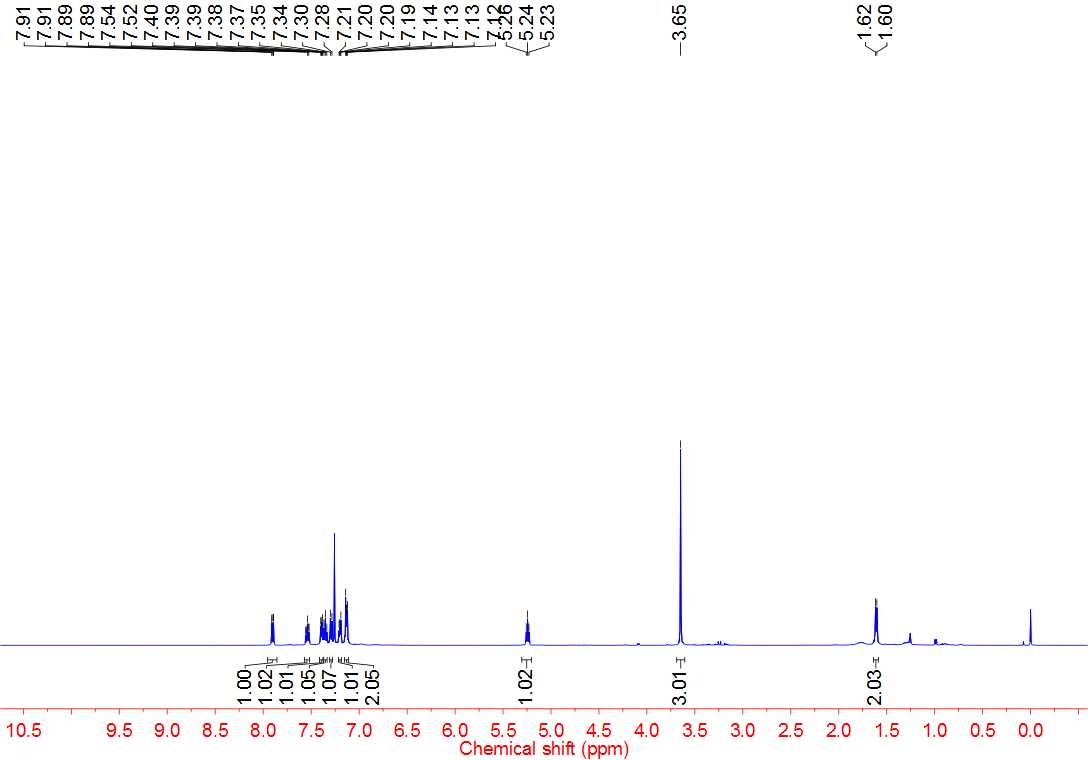
**

**4ai ^13^C NMR**

**
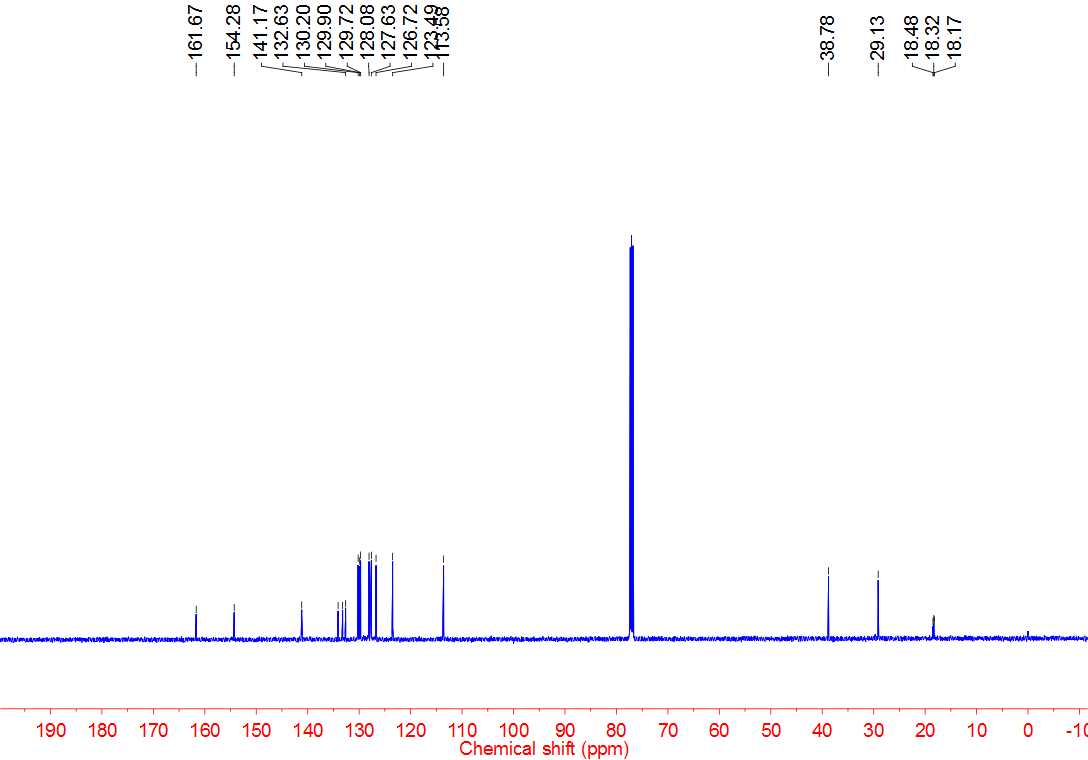
**

**4aj ^1^H NMR**

**
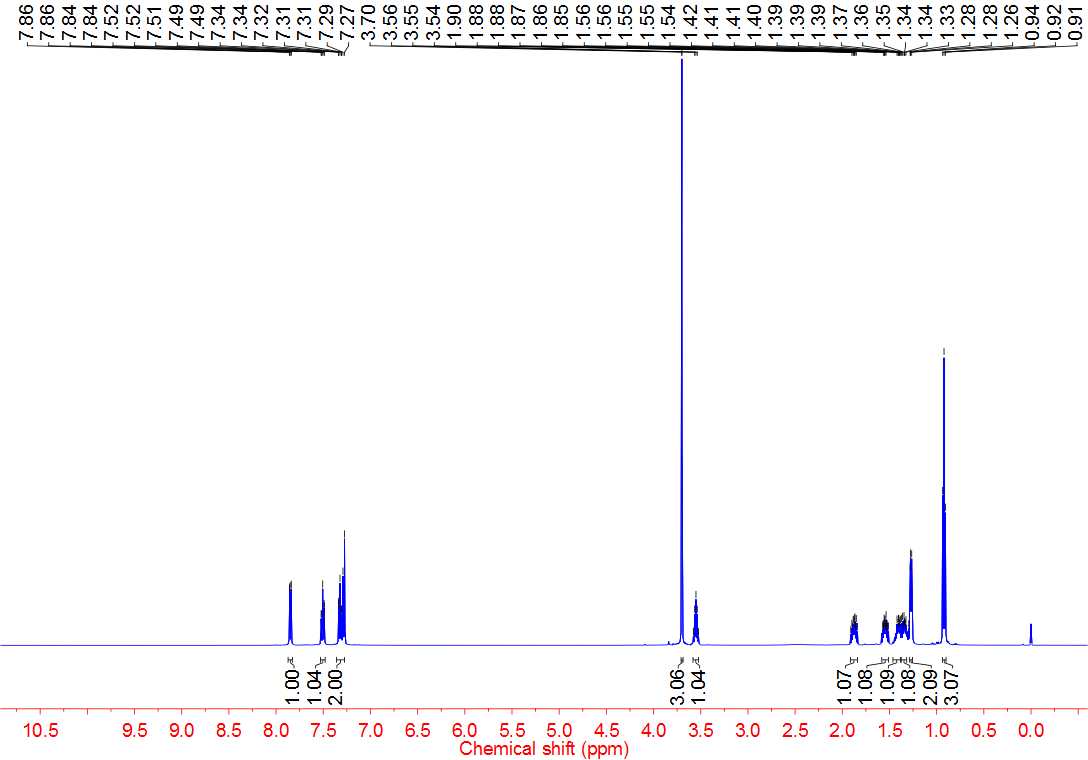
**

**4aj ^13^C NMR**

**
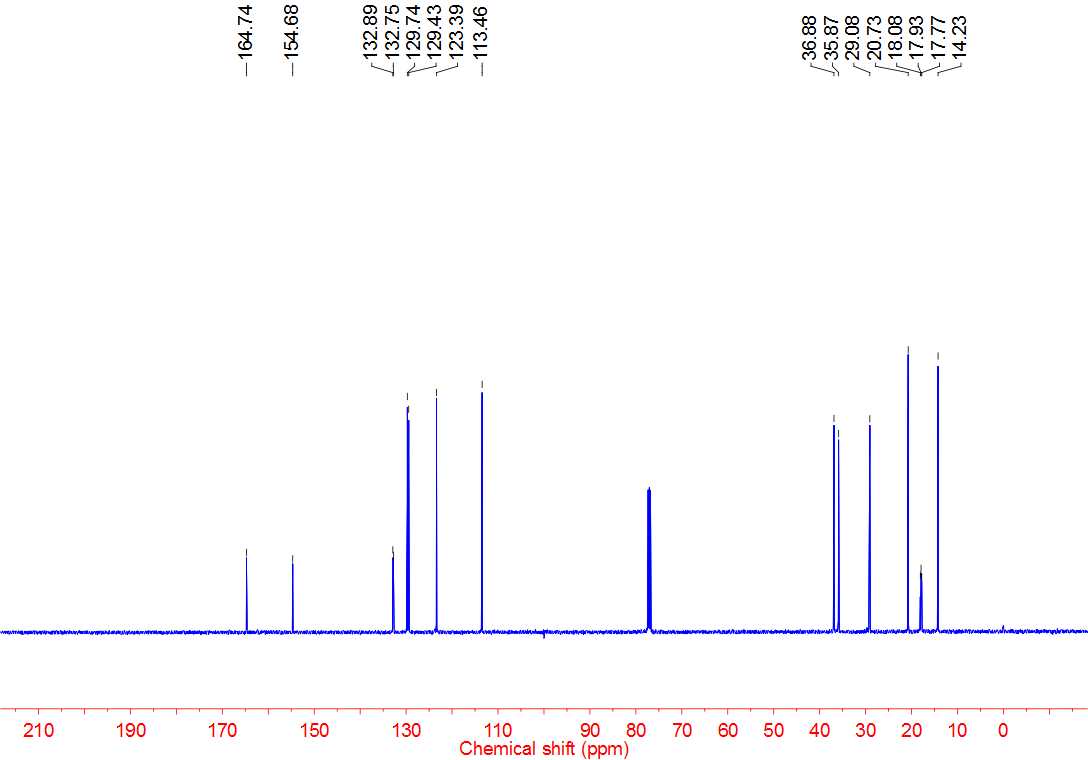
**

**4ak ^1^H NMR**

**
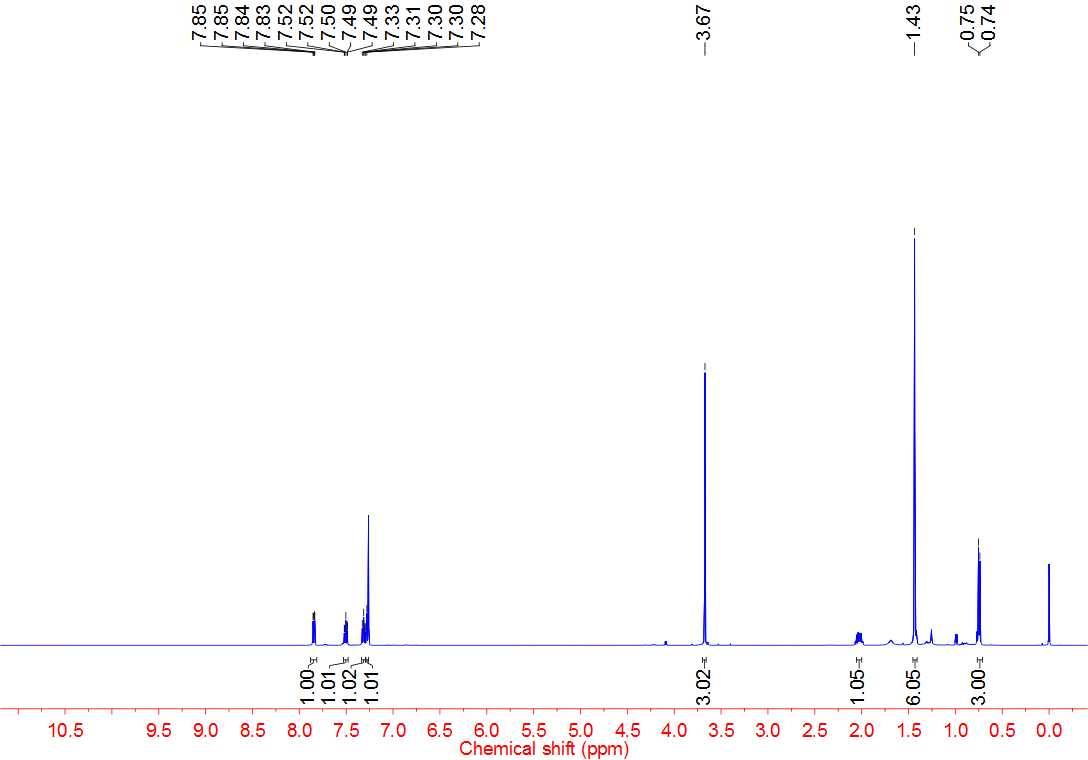
**

**4ak ^13^C NMR**

**
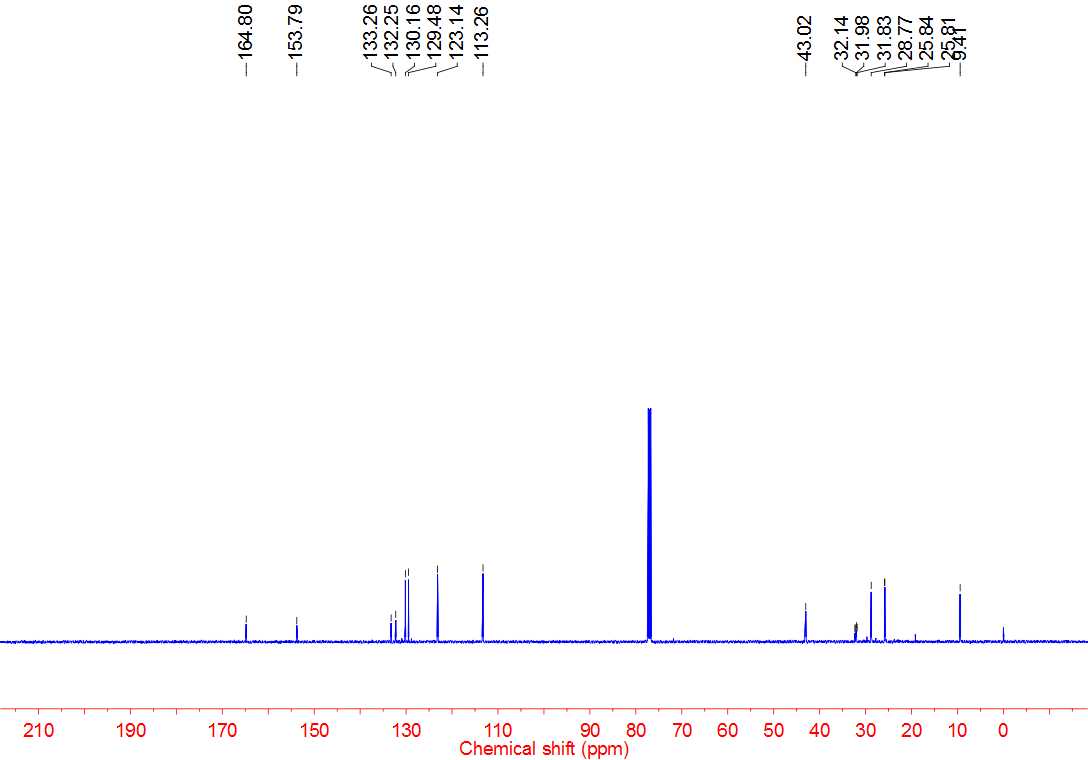
**

**4al ^1^H NMR**

**
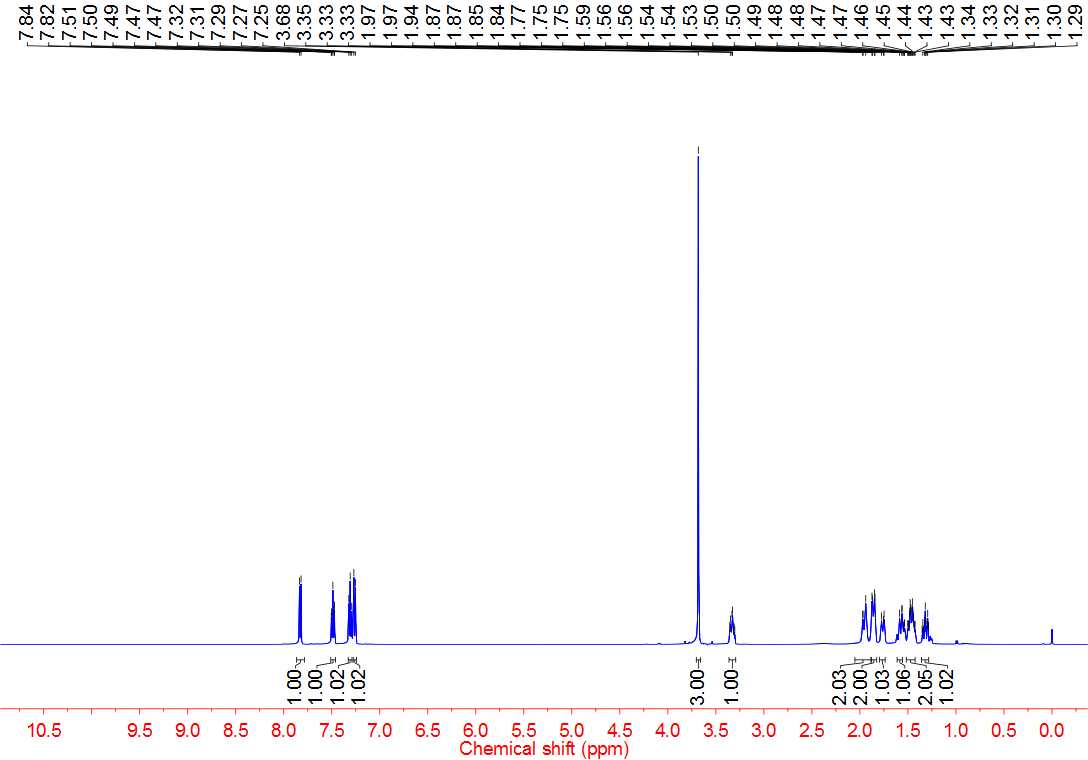
**

**4al ^13^C NMR**

**
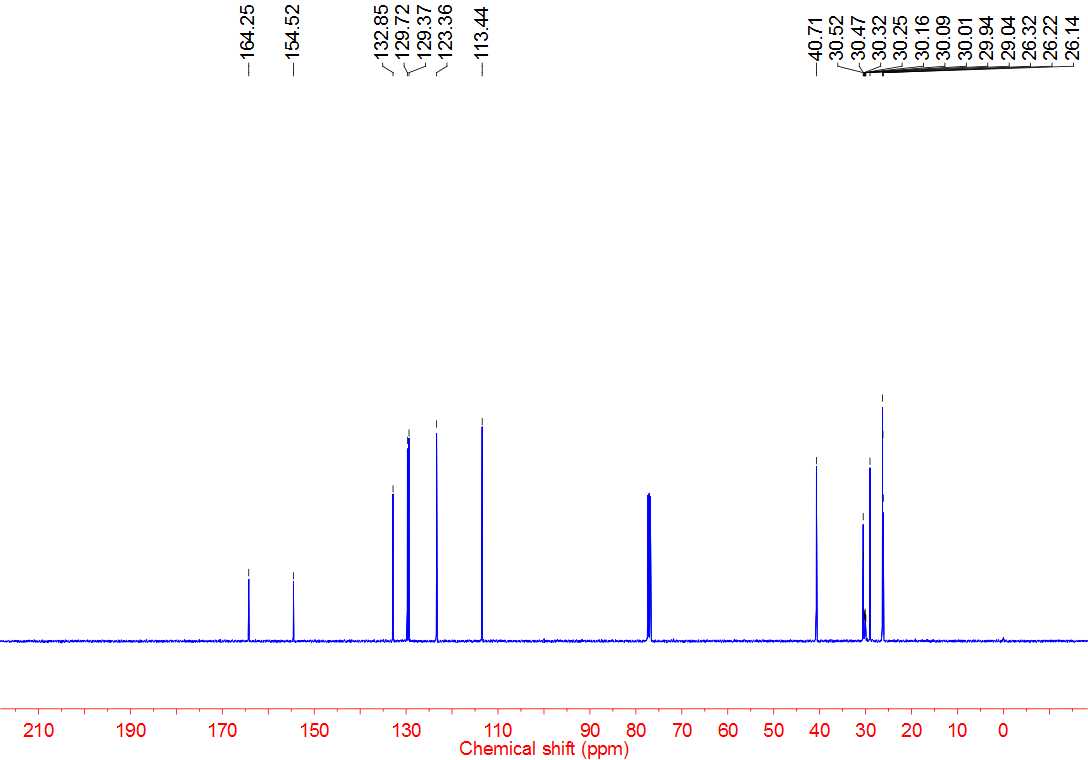
**

**4am ^1^H NMR**

**
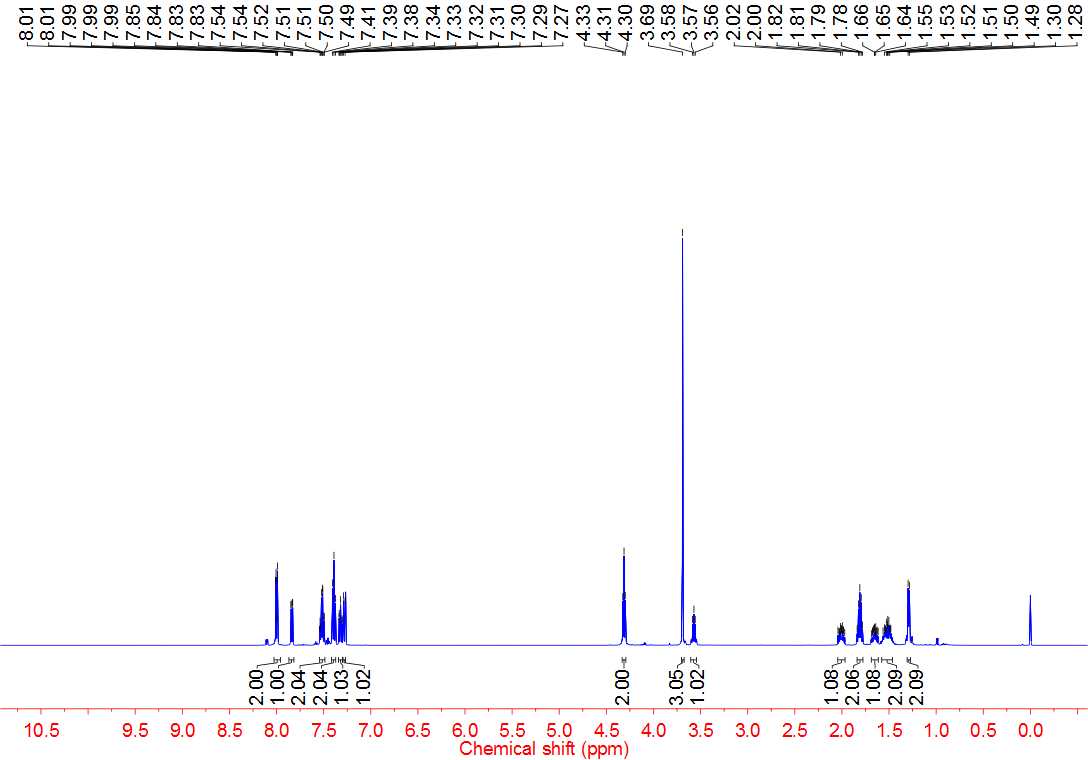
**

**4am ^13^C NMR**

**
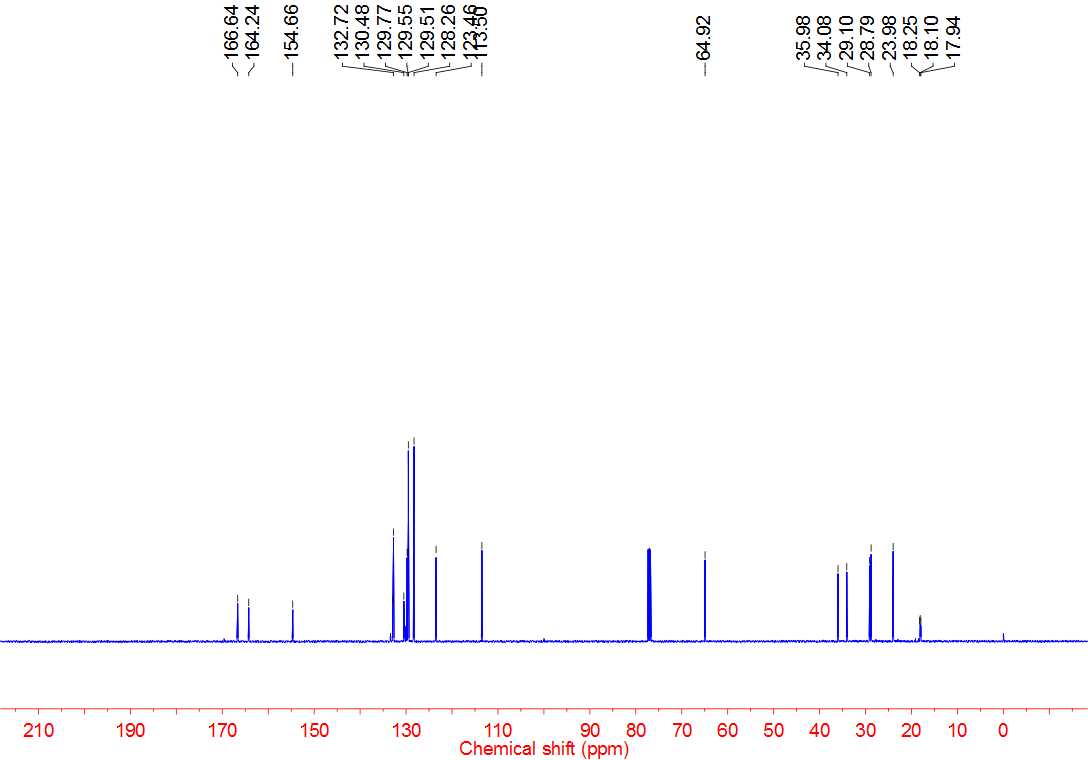
**

**4an ^1^H NMR**

**
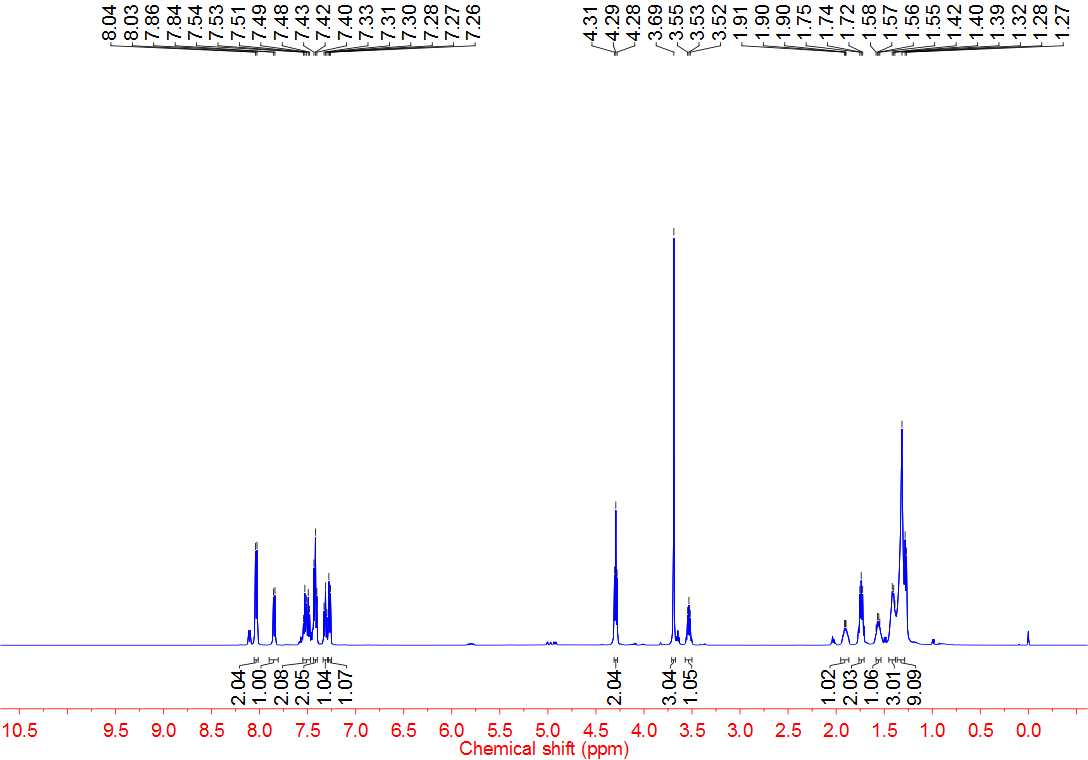
**

**4an ^13^C NMR**

**
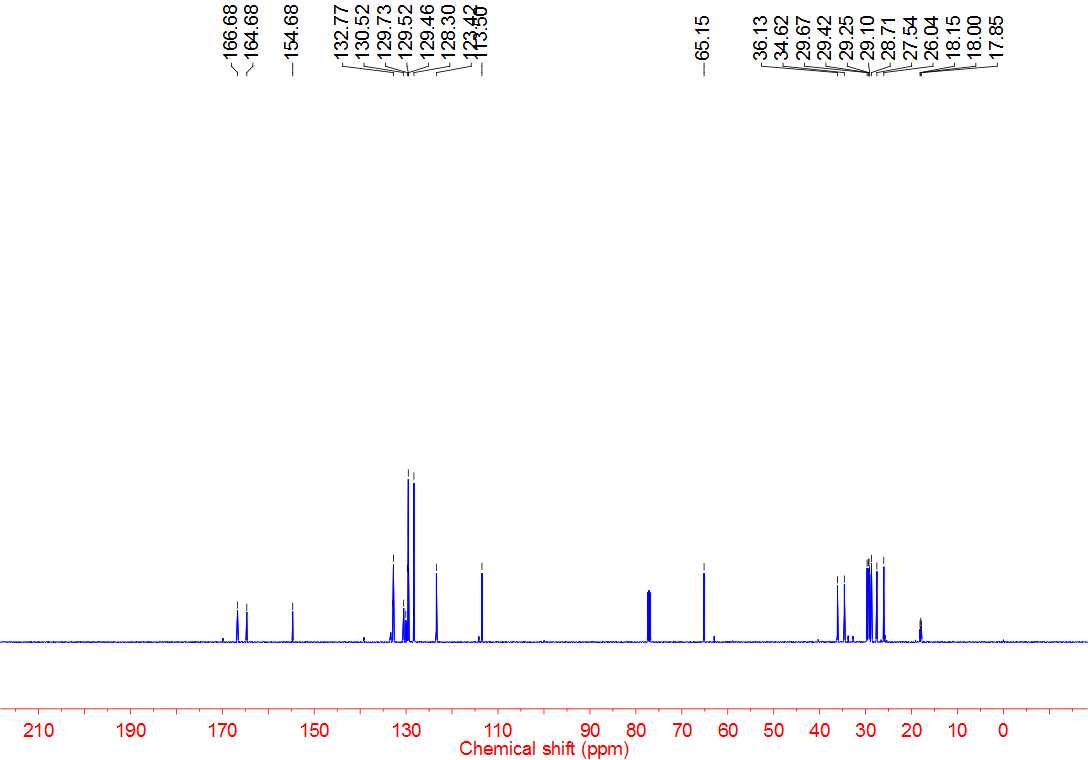
**

**4ao ^1^H NMR**

**
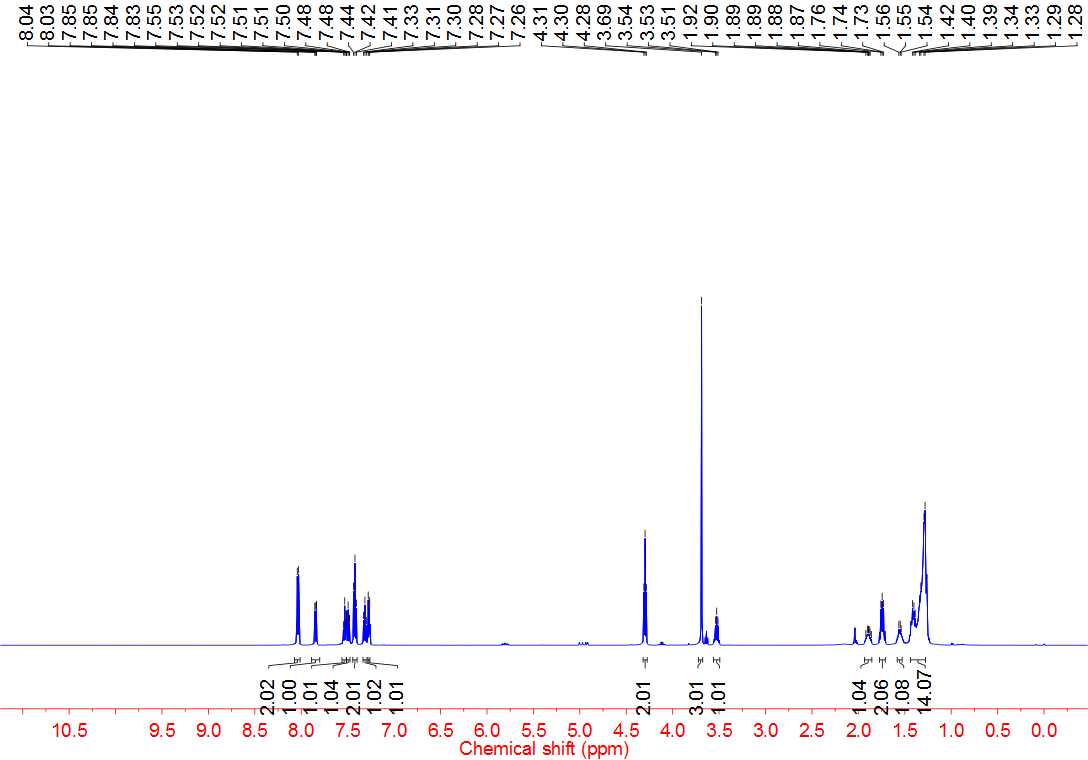
**

**4ao ^13^C NMR**

**
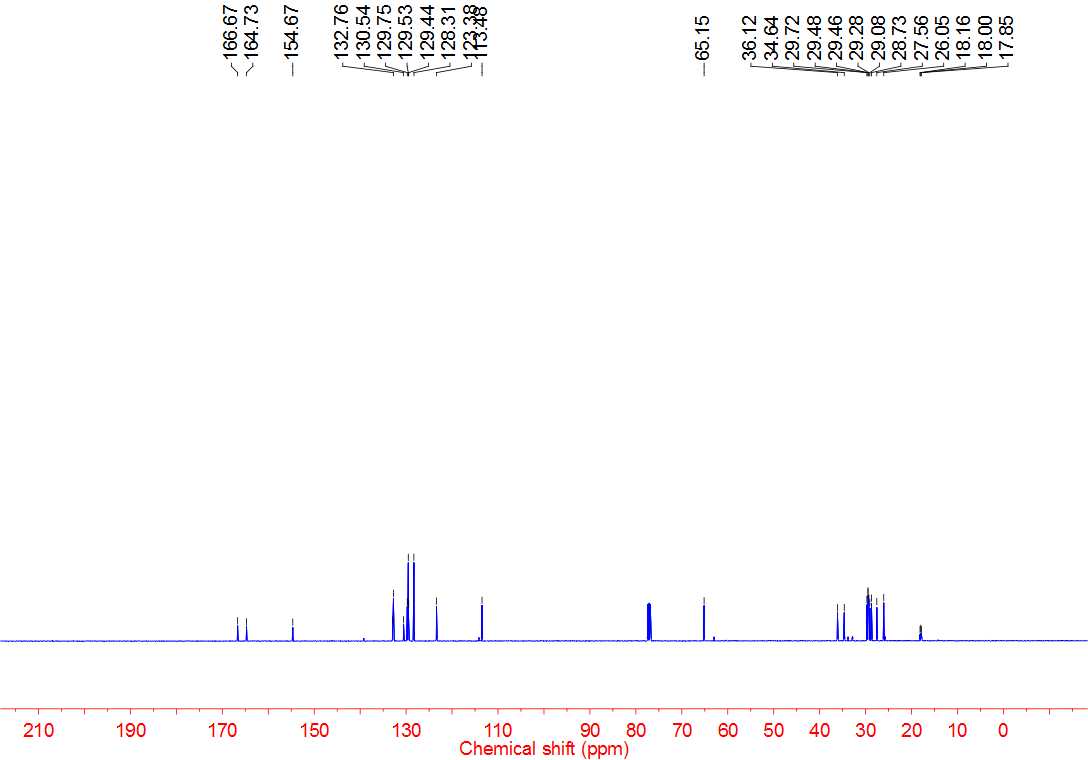
**

**4ap ^1^H NMR**

**
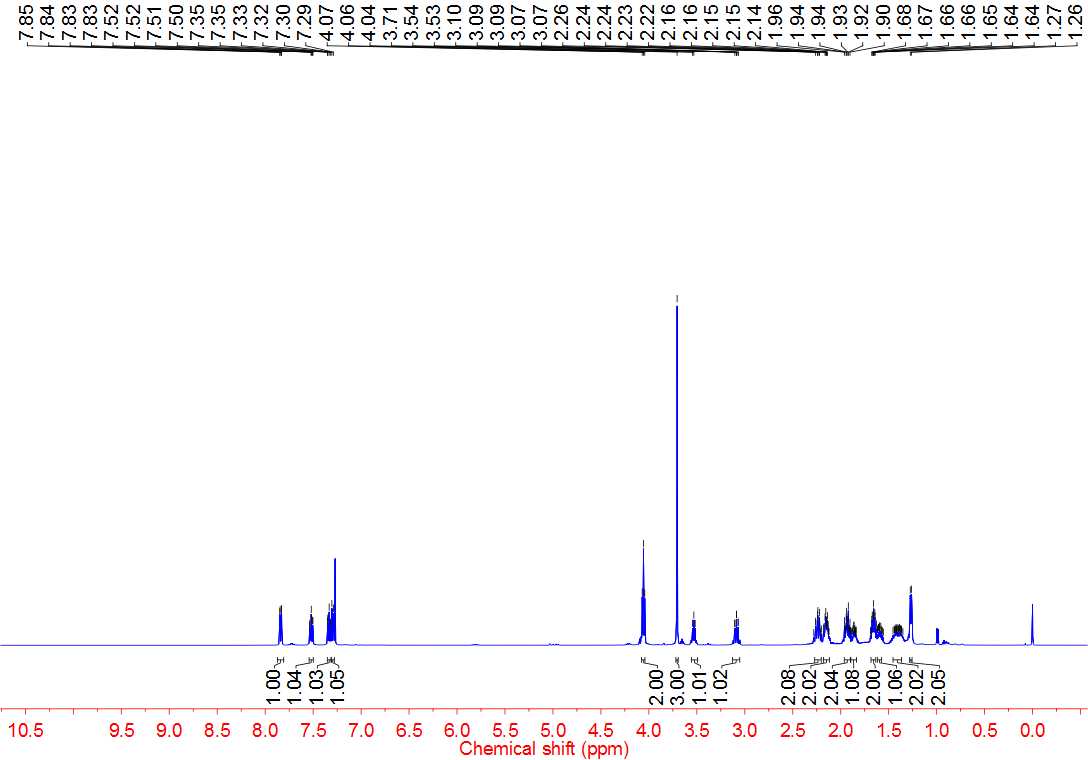
**

**4ap ^13^C NMR**

**
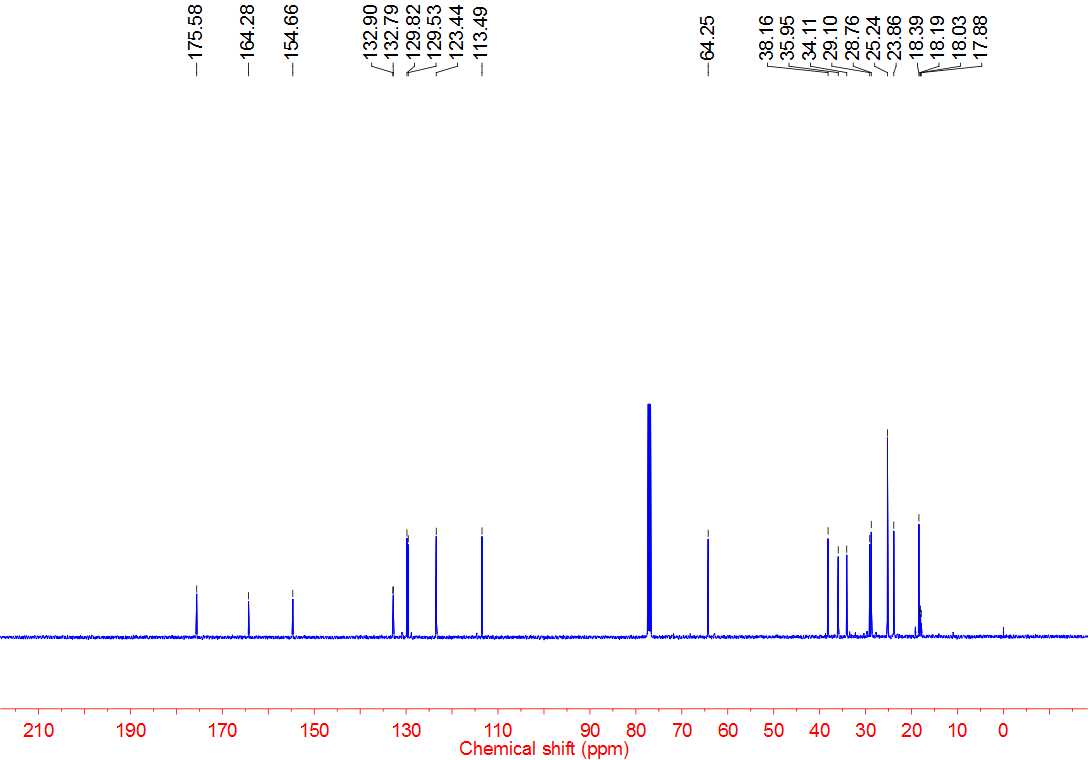
**

**4aq ^1^H NMR**

**
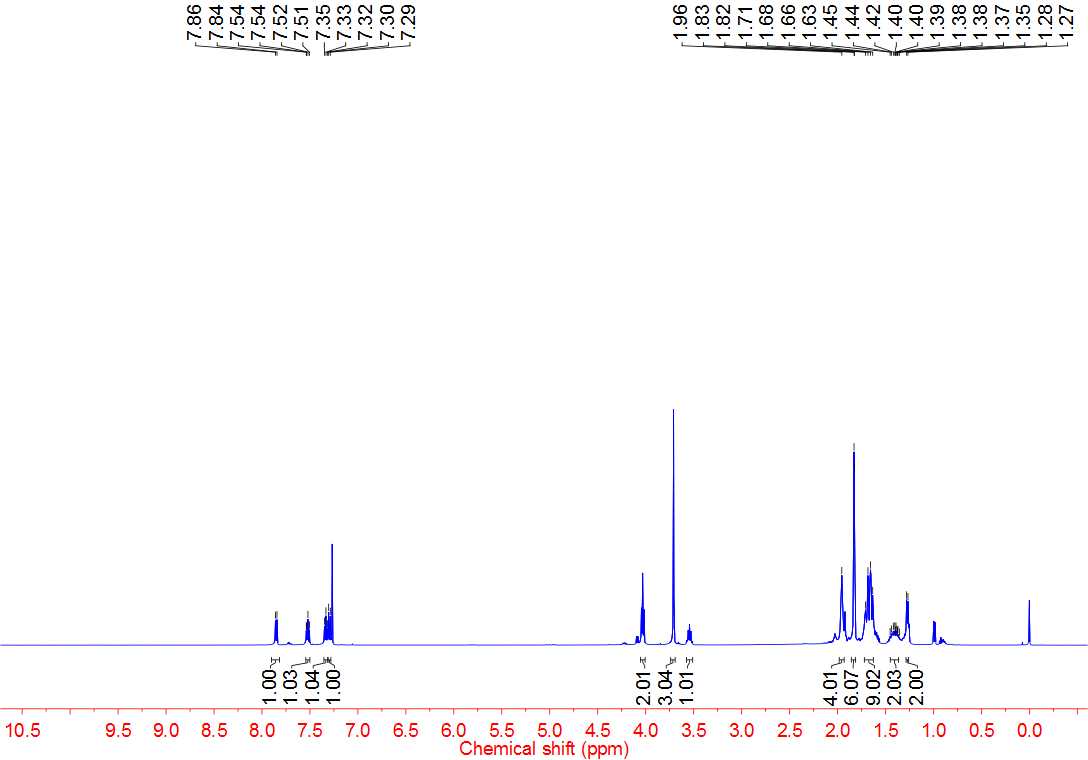
**

**4aq ^13^C NMR**

**
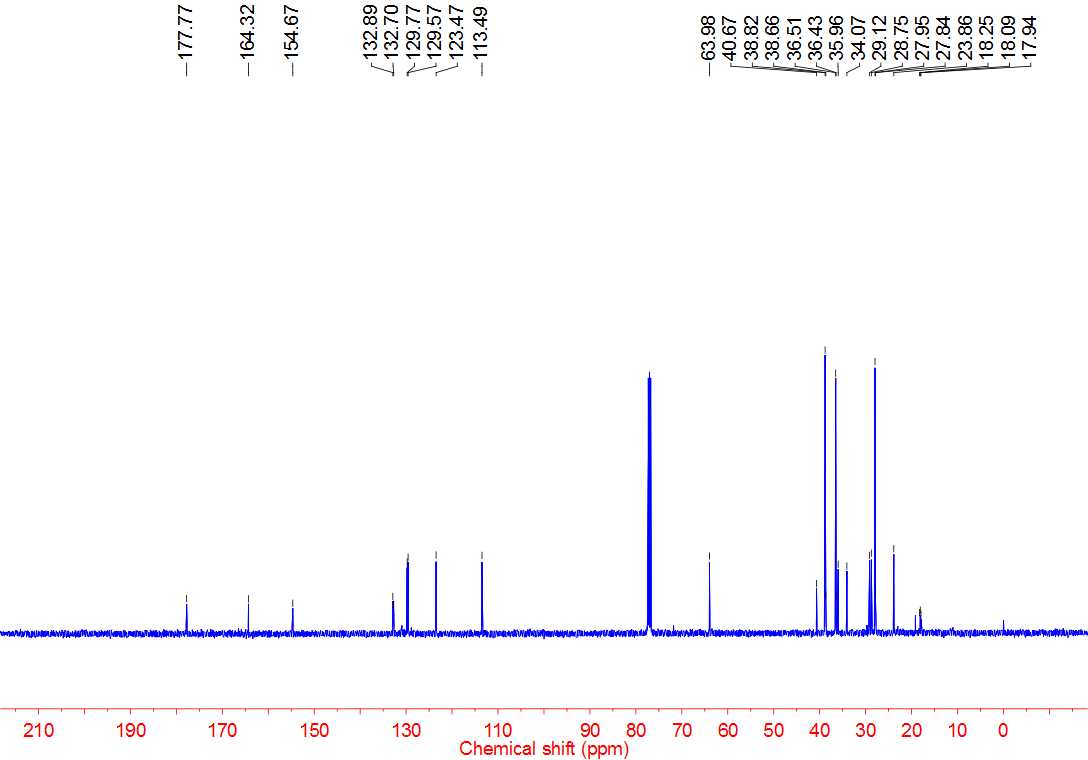
**

**4ar ^1^H NMR**

**
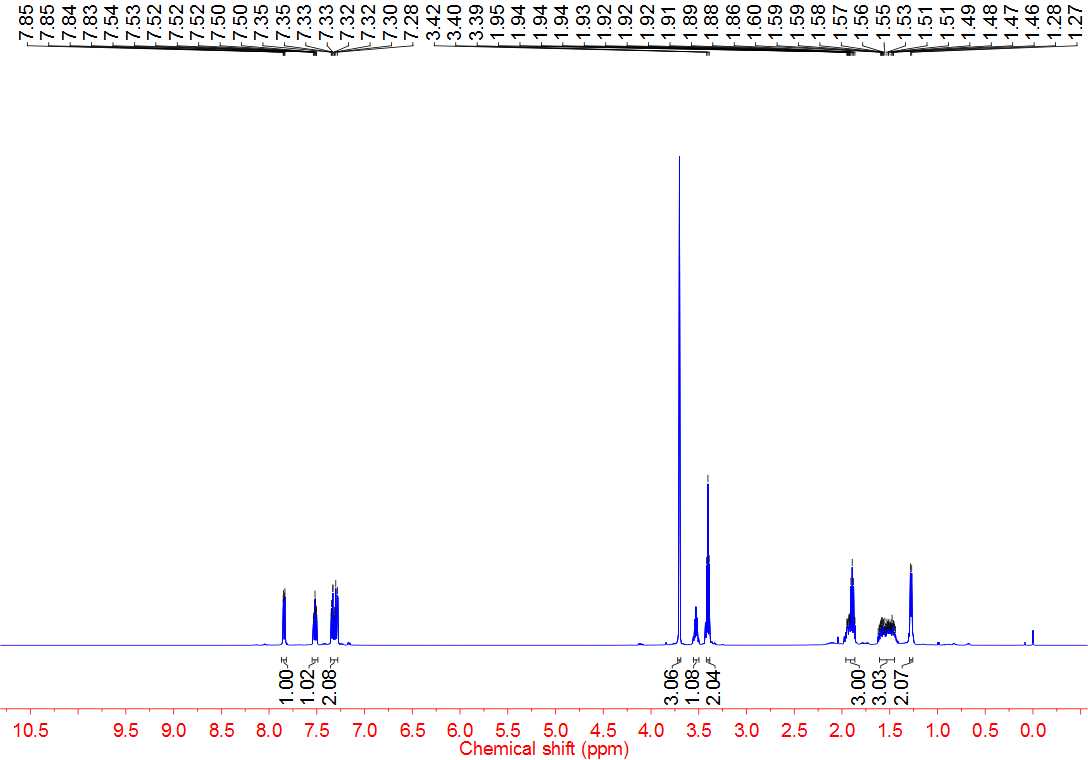
**

**4ar ^13^C NMR**

**
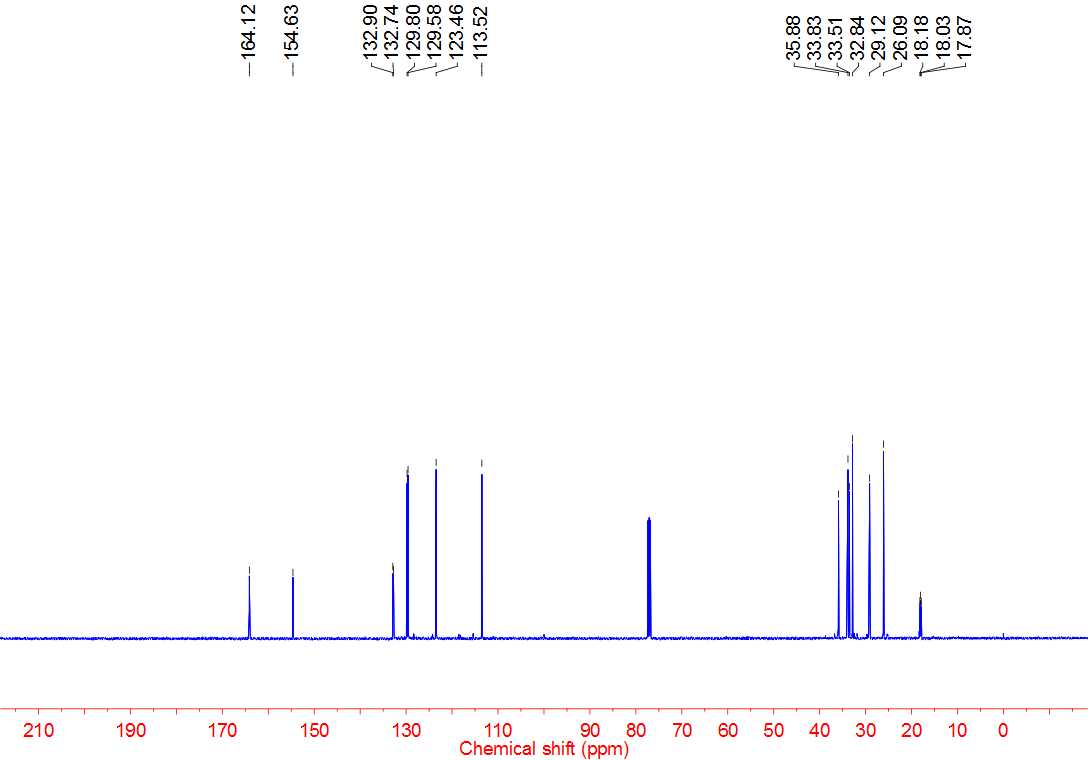
**

**4as ^1^H NMR**

**
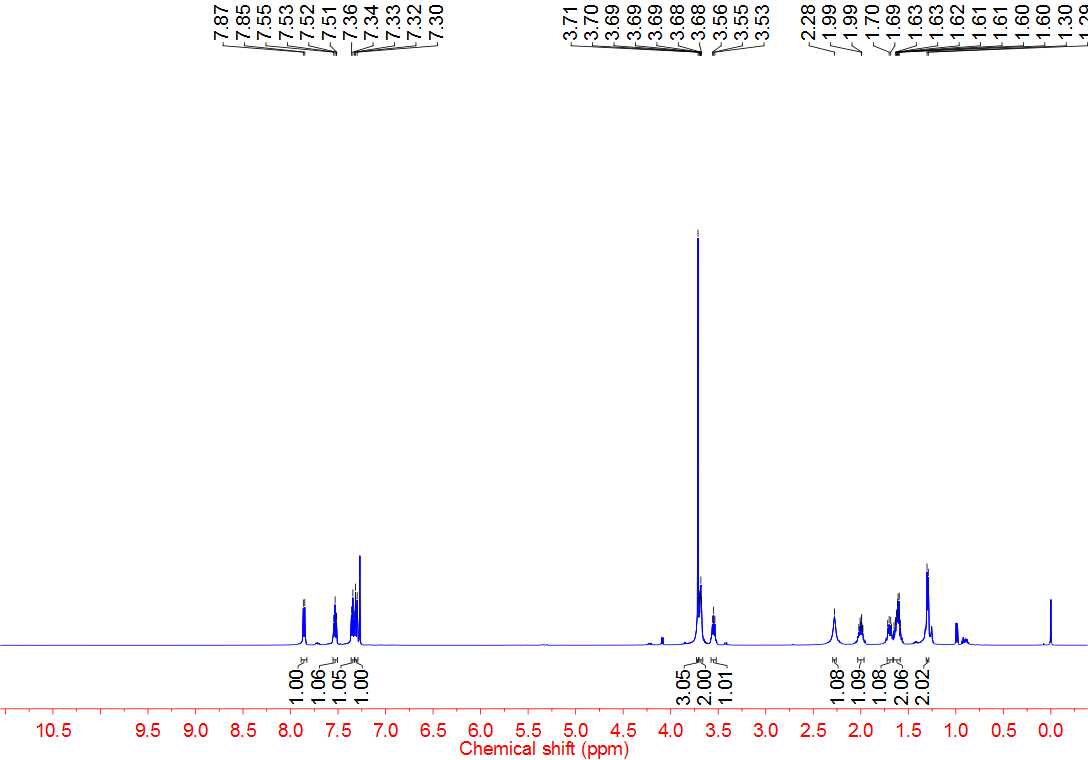
**

**4as ^13^C NMR**

**
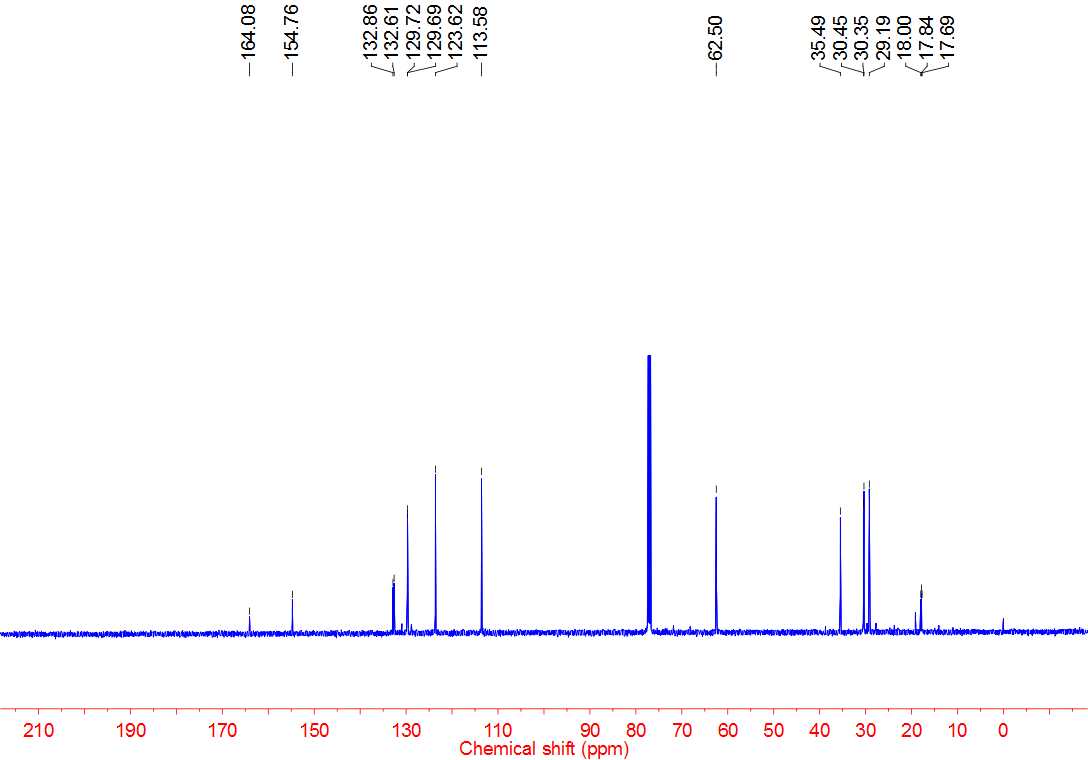
**

**4at ^1^H NMR**

**
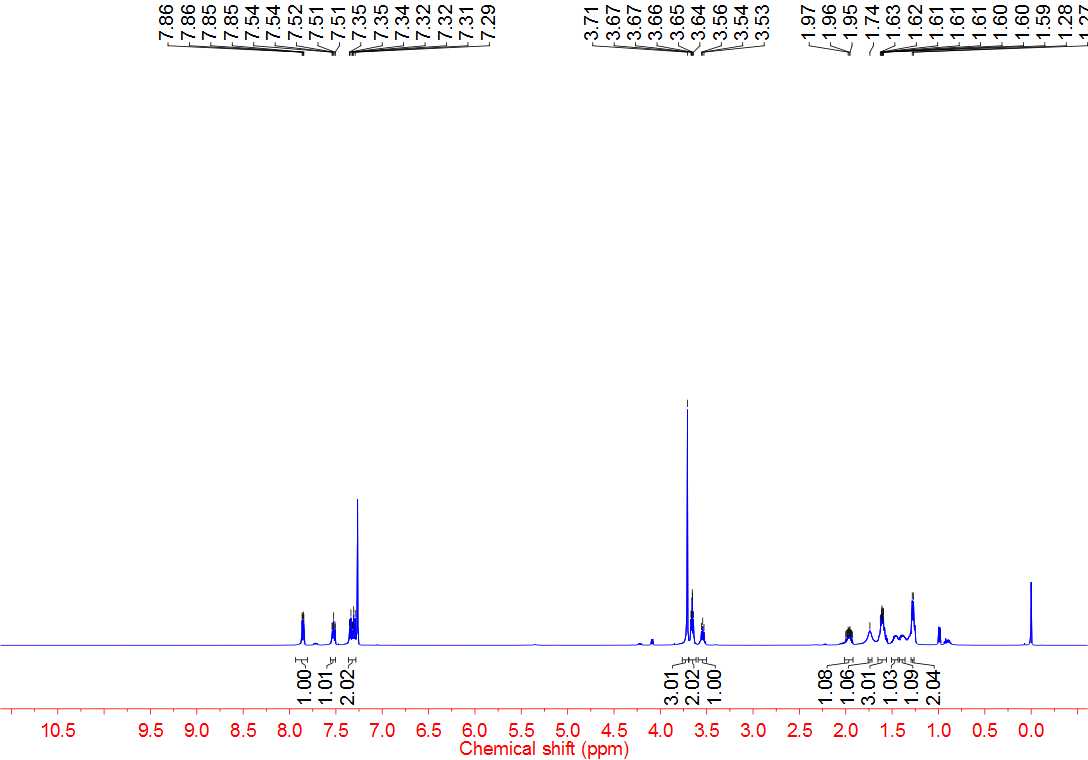
**

**4at ^13^C NMR**

**
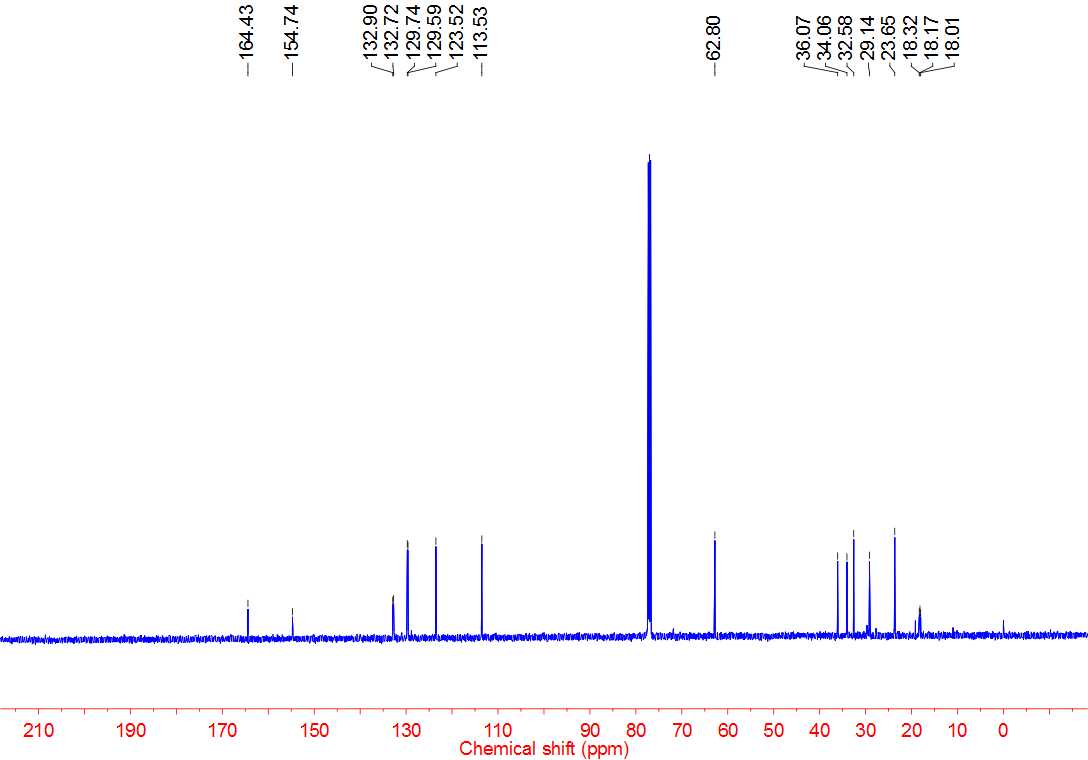
**

**4au ^1^H NMR**

**
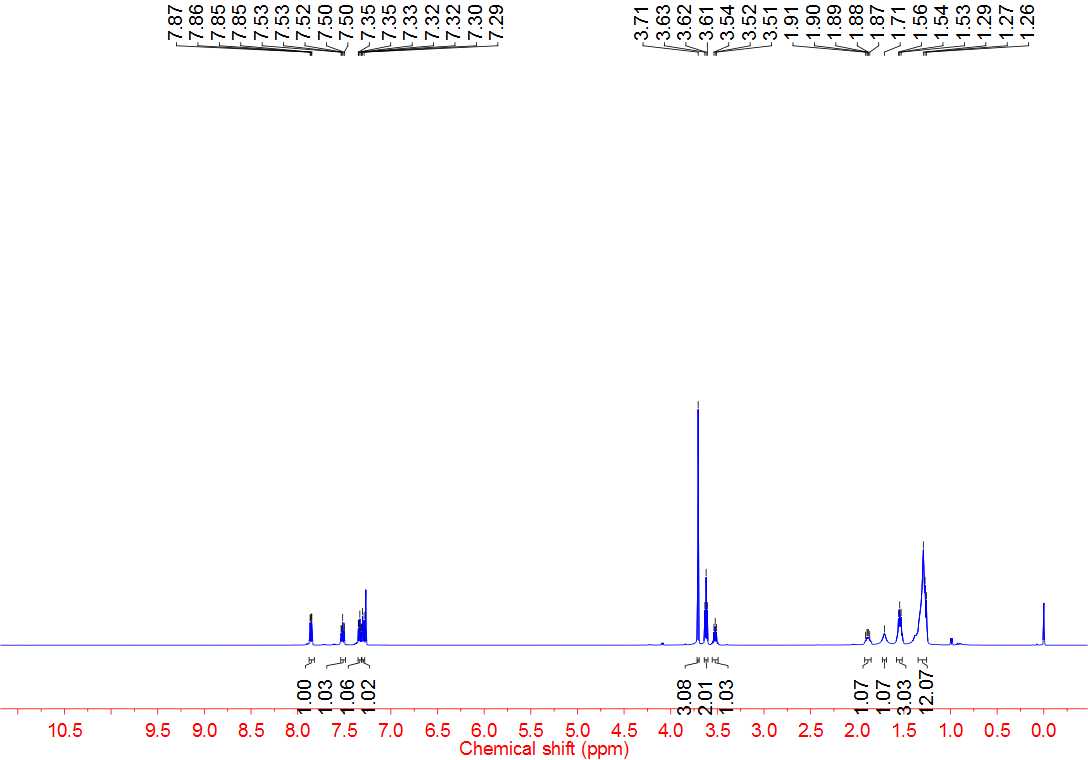
**

**4au ^13^C NMR**

**
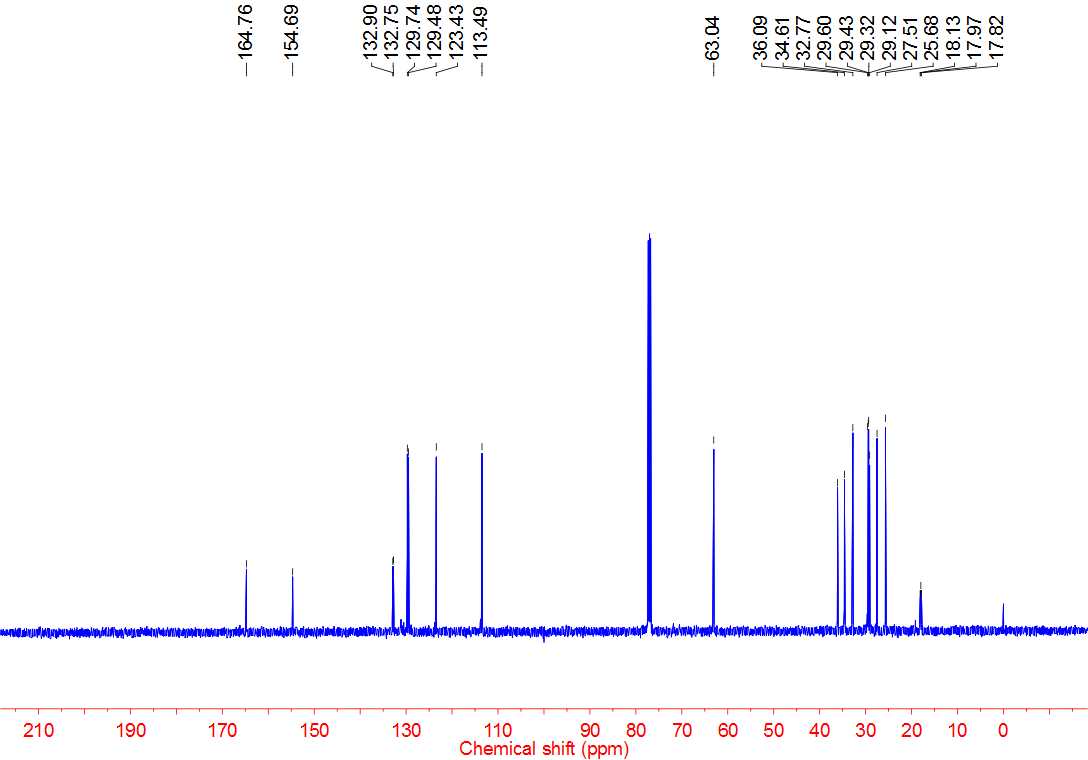
**

**4av ^1^H NMR**

**
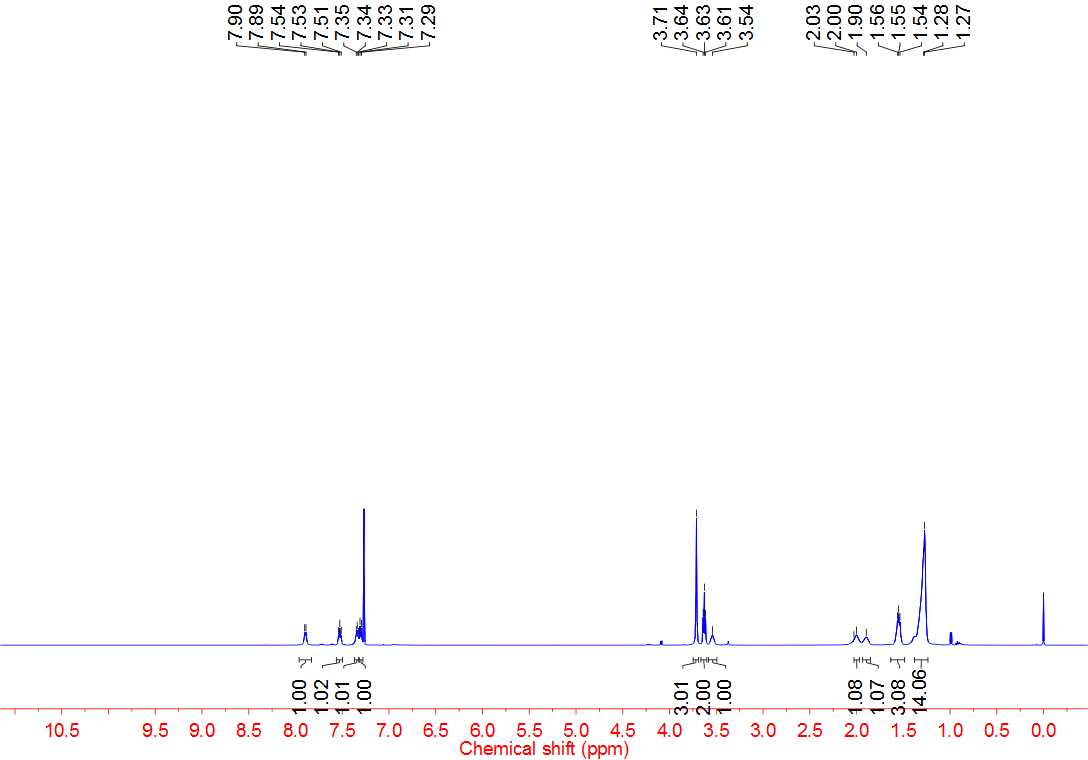
**

**4av ^13^C NMR**

**
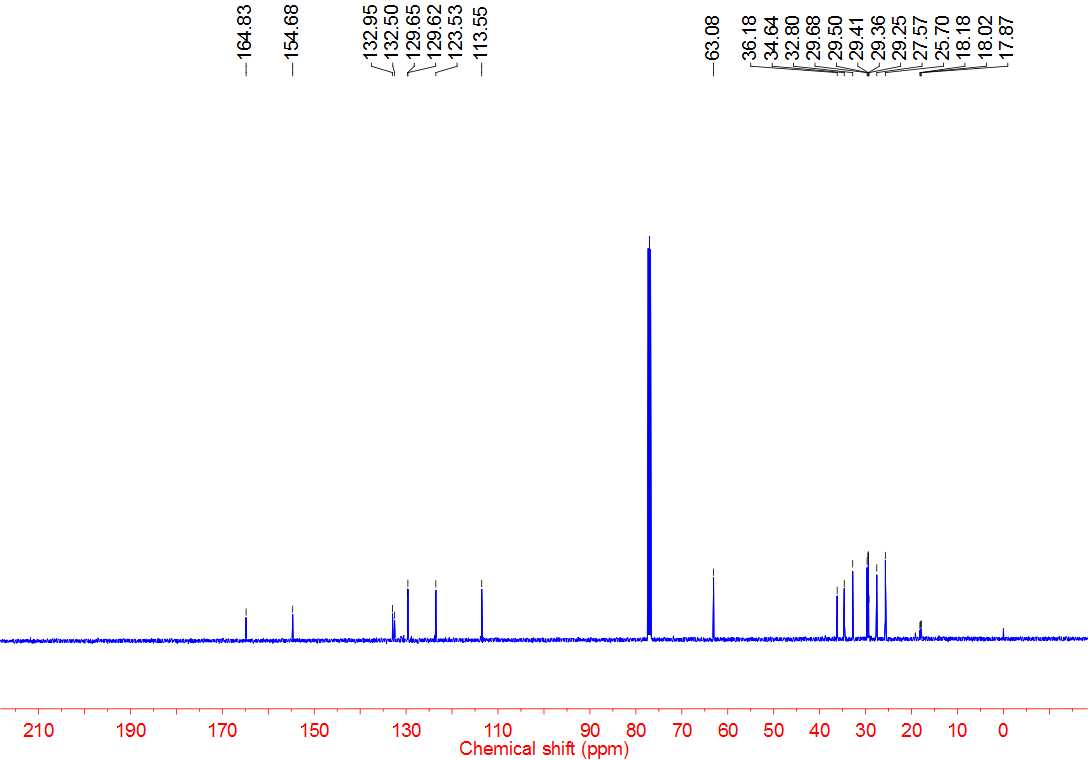
**

**4bb ^1^H NMR**

**
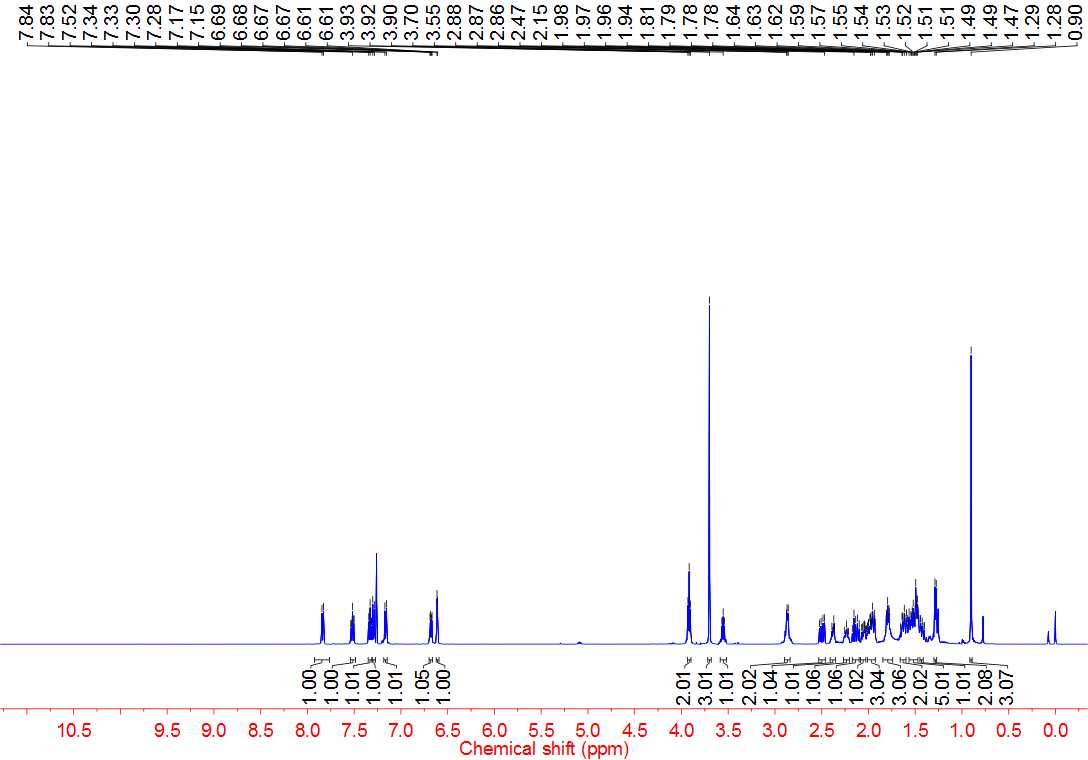
**

**4bb ^13^C NMR**

**
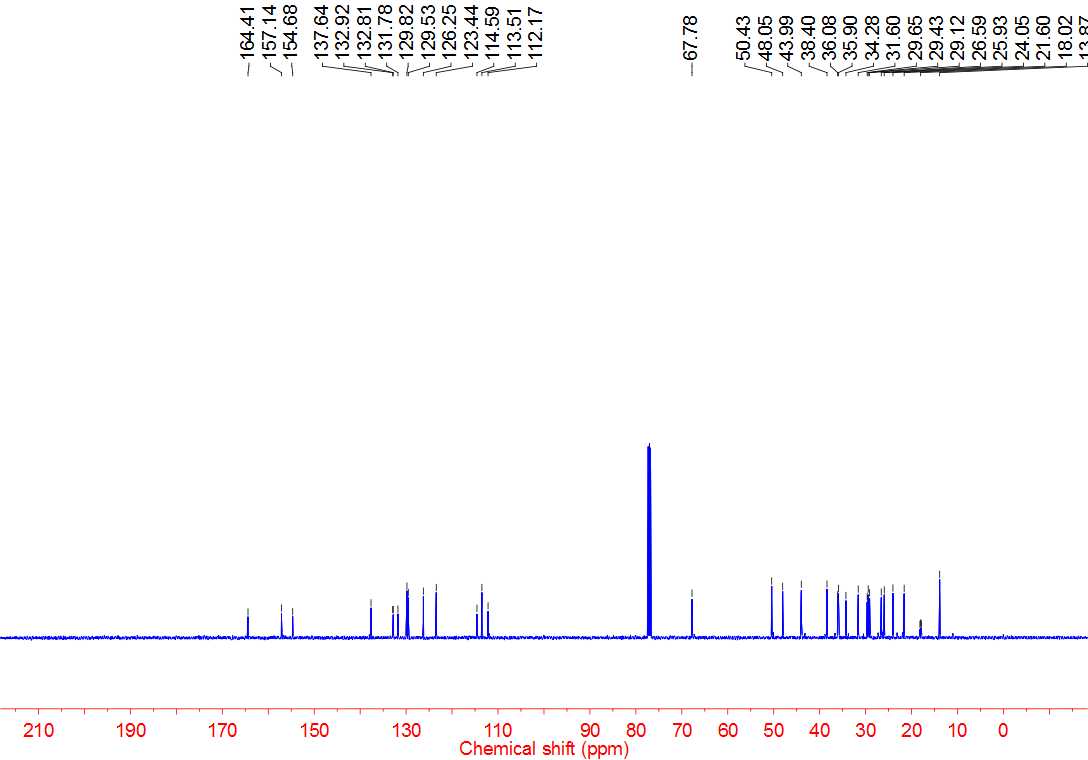
**

**6a ^1^H NMR**


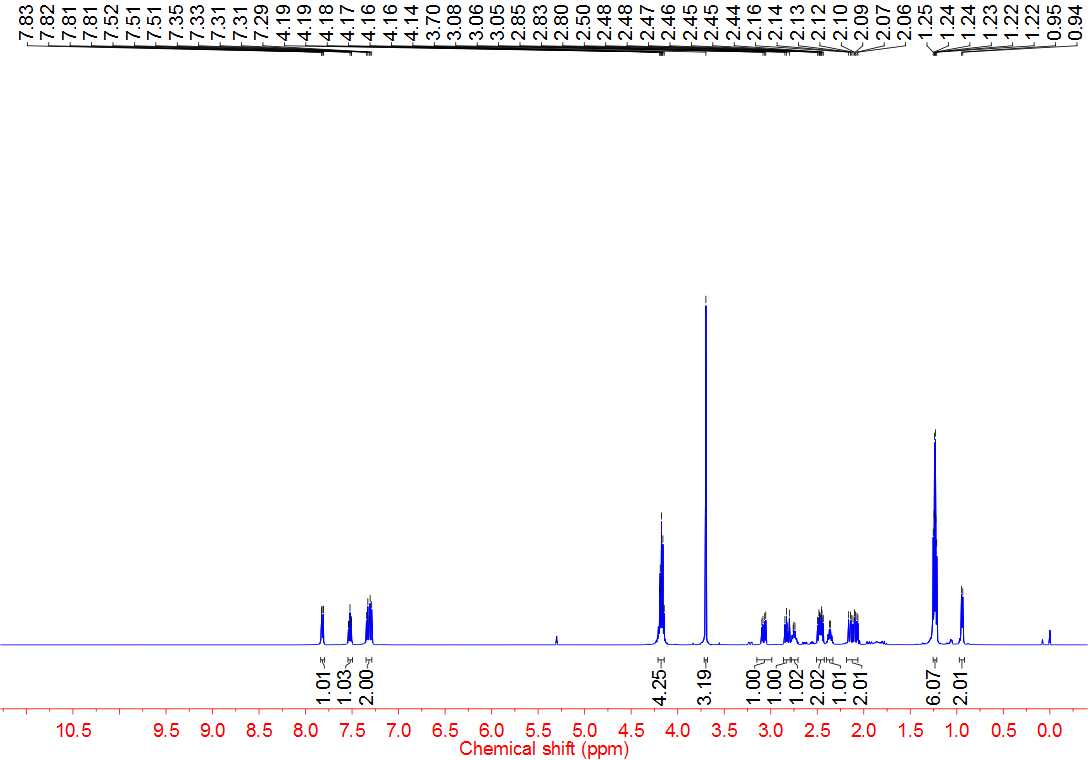


**6a ^13^C NMR**


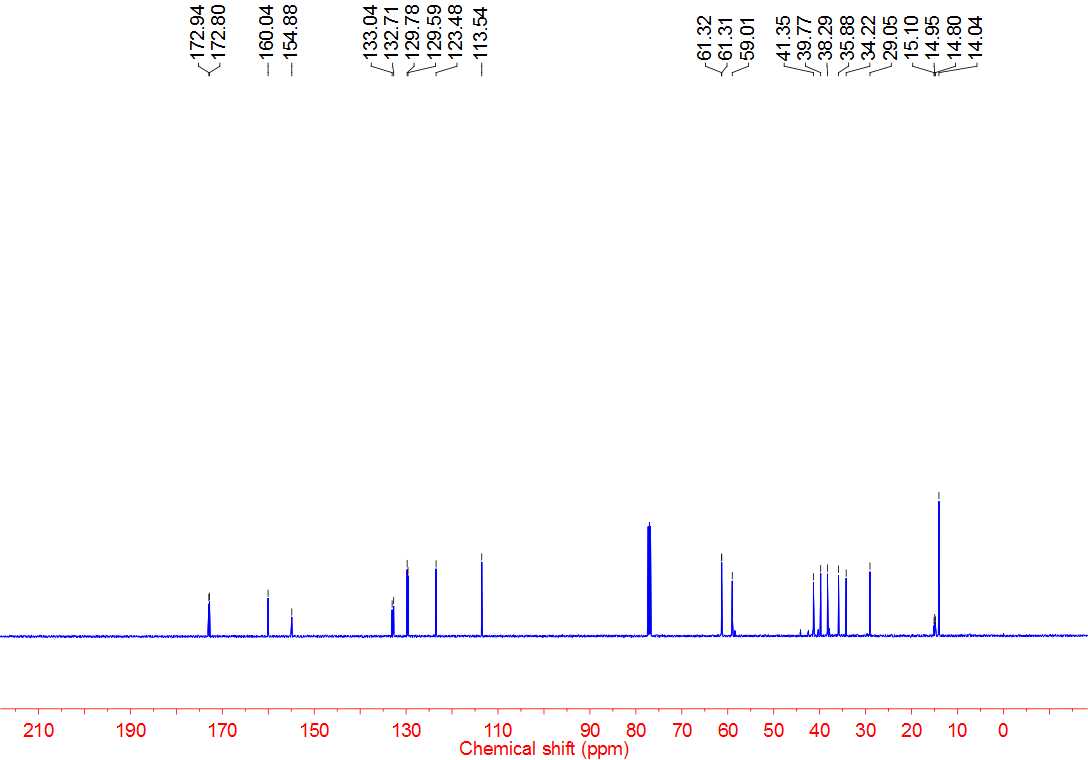

Supplement: Supplementary file 1 [file Data_Sheet_1.docx]
